# Evaluating Performance of Different RNA Secondary Structure Prediction Programs Using Self-cleaving Ribozymes

**DOI:** 10.1093/gpbjnl/qzae043

**Published:** 2024-06-08

**Authors:** Fei Qi, Junjie Chen, Yue Chen, Jianfeng Sun, Yiting Lin, Zipeng Chen, Philipp Kapranov

**Affiliations:** State Key Laboratory of Cellular Stress Biology, School of Life Sciences, Faculty of Medicine and Life Sciences, Xiamen University, Xiamen 361102, China; Institute of Genomics, School of Medicine, Huaqiao University, Xiamen 361021, China; Institute of Genomics, School of Medicine, Huaqiao University, Xiamen 361021, China; Institute of Genomics, School of Medicine, Huaqiao University, Xiamen 361021, China; Botnar Research Centre, University of Oxford, Oxford, OX3 7LD, United Kingdom; Institute of Genomics, School of Medicine, Huaqiao University, Xiamen 361021, China; Institute of Genomics, School of Medicine, Huaqiao University, Xiamen 361021, China; State Key Laboratory of Cellular Stress Biology, School of Life Sciences, Faculty of Medicine and Life Sciences, Xiamen University, Xiamen 361102, China

**Keywords:** RNA secondary structure, RNA secondary structure prediction, Ribozyme, Deep learning, Pseudoknot

## Abstract

Accurate identification of the correct, biologically relevant RNA structures is critical to understanding various aspects of RNA biology since proper folding represents the key to the functionality of all types of RNA molecules and plays pivotal roles in many essential biological processes. Thus, a plethora of approaches have been developed to predict, identify, or solve RNA structures based on various computational, molecular, genetic, chemical, or physicochemical strategies. Purely computational approaches hold distinct advantages over all other strategies in terms of the ease of implementation, time, speed, cost, and throughput, but they strongly underperform in terms of accuracy that significantly limits their broader application. Nonetheless, the advantages of these methods led to a steady development of multiple *in silico* RNA secondary structure prediction approaches including recent deep learning-based programs. Here, we compared the accuracy of predictions of biologically relevant secondary structures of dozens of self-cleaving ribozyme sequences using seven *in silico* RNA folding prediction tools with tasks of varying complexity. We found that while many programs performed well in relatively simple tasks, their performance varied significantly in more complex RNA folding problems. However, in general, a modern deep learning method outperformed the other programs in the complex tasks in predicting the RNA secondary structures, at least based on the specific class of sequences tested, suggesting that it may represent the future of RNA structure prediction algorithms.

## Introduction

RNA molecules can fold into complex secondary and tertiary structures that are often critical for virtually all aspects of metabolism, processing, and functionality of multiple classes of RNAs and RNA elements [1–4]. For example, RNA structure can impact translation [5–7], splicing [8], and RNA–protein interactions [9]. Proper folding is required for processing of small regulatory RNAs, such as microRNAs [10]. RNA structure is critical for the functionality of sensing RNA domains (such as riboswitches) that regulate longer transcripts in which they are embedded [11]. Long non-coding RNAs (lncRNAs) that represent the majority of transcriptional output of the human genome [12,13] are believed to function at least in part via folding into specific RNA conformations and/or carrying specific RNA structural elements [14–17]. Proper secondary and tertiary structures are required for the catalytic activity of RNA enzymes, known as ribozymes, which are involved in key cellular processes such as translation and epigenetic regulation [18,19].

Developing methods to predict and characterize RNA structures has therefore attracted a considerable amount of interest. A slew of methodologies that could be applied to characterize RNA structures in a high-throughput manner have been developed, and could be subdivided into two classes. The first class relies on purely *in silico* analysis of RNA sequences [20–22], and the second class involves directly probing RNA structures using genetic, chemical [23–26], and physicochemical approaches [27,28]. Relatively easy and quick implementations are the major natural advantages of the *in silico* methods relative to the empirical ones that are laborious, complex, or costly, and may require specialized equipment and skills [22]. However, RNA structures predicted solely by computational methods are often unreliable and may not accurately represent the authentic functional structures [29].

Inclusion of additional data, like evolutionary information from homologous sequences, has been shown to enhance the accuracy of *in silico* RNA structure predictions [30]. For example, Singh et al. achieved a significant improvement of SPOT-RNA [31] in its updated version, SPOT-RNA2, by incorporating evolutionary information [30]. When predicting RNA secondary structures, SPOT-RNA2 performs a fully automatic 2-round homologous search using first BLAST-N and then Infernal against the National Center of Biotechnology Information (NCBI) nucleotide collection (nt) database [30]. The resulting multiple sequence alignment of homologous sequences is used to generate the position specific score matrix (PSSM) and two-dimensional direct coupling analysis (DCA) information which are then used as the input into the deep neural network [30]. However, this program, as indicated by the authors, is not suitable for RNAs with very few functional homologs [30], such as recently evolved functional RNAs exemplified by the self-cleaving hovlinc ribozymes that exist in many species and share over 90% sequence identity within simians [32]. However, since only the sequences from humans, chimpanzee, and bonobo, which share ∼ 99% sequence identity, exhibit the self-cleavage activity [32], even the close homologs from other simians may provide no useful information related to the active structure.

Therefore, a clear need exists for reliable computational *ab initio* RNA structure prediction tools that can work on sequences of RNAs of interest without any additional information. Indeed, a number of such tools have been developed [22]; however, the relative performance of these tools has not been characterized. Furthermore, recent technological progress posed a number of questions. For example, the development of RNA structure prediction methods based on the deep learning approach [33] prompted a natural question of whether such methods are better than the classical folding-based approaches. Also, availability of high-throughput datasets that compare effects of thousands of random sequence changes in the same RNA species on its activity [34,35] provoked a question of whether any of the RNA structure prediction programs are good enough to measure relatively small yet functionally-relevant changes in RNA structures. Furthermore, combining various sensing and catalytic RNA domains to create complex RNA-based functional modules in context of longer sequences regulated by these modules has long been suggested to have strong potential in synthetic biology [36–38]. However, the best way to predict correct folding of RNA domains in a larger sequence context remains unclear.

In this work, we aimed to fill the gap created by these questions by analyzing the performance of different RNA secondary structure prediction programs using the known secondary structures of 32 functional RNA sequences representing self-cleaving ribozymes. Ribozymes have certain key features that make them ideal molecules to test the performance of RNA secondary structure prediction programs. First, secondary, and in some cases tertiary, structures of the ribozymes are well characterized [39–41]. Second, changes to the proper folding pattern of a ribozyme can be fairly accurately reflected by changes in its activity that is relatively easy to measure [34,35,41]. Therefore, the effects of sequence changes on ribozyme activity and, thus, secondary structure can be measured in a high-throughput manner, which can serve as an important resource to gauge the performance of RNA secondary structure prediction programs.

Here, we tested programs that satisfy four conditions. First, the programs must be able to predict pseudoknots that are vital for the activities of most if not all self-cleaving ribozymes and multiple other classes of functional RNAs [41,42], but are very challenging to accurately identify [43] (see below). Second, the programs must be able to take the native RNA sequences as the solo input, *i.e.*, a program should be able to predict the secondary structure of an RNA based on its nucleotide sequence only. Third, the programs must be freely available as standalone applications. Many of the RNA structure prediction programs are provided only in the form of webservers. While useful for low-throughput RNA folding applications that could be performed by users with little bioinformatics expertise, webserver-based solutions are unsuitable for high-throughput RNA structure discovery or characterization, or when programs need to be merged into custom bioinformatics pipelines. Finally, the programs must predict the secondary structure within a reasonable timeframe, especially for long sequences.

Applying the aforementioned criteria, we selected seven RNA secondary structure prediction programs, including five classical folding-based programs ProbKnot [44], RNAPKplex [45], pKiss [46], Knotty [47], and IPknot [48,49] published between 2010 and 2022, and two deep learning-based programs SPOT-RNA [31] and UFold [50] published in 2019 and 2022, respectively. All these seven programs are designed to predict RNA secondary structures including pseudoknots purely based on the RNA sequences, and are available as standalone executable applications. While all programs performed adequately in some comparisons, the deep learning-based method SPOT-RNA either outperformed the other approaches or showed the second-best characteristics in some key comparisons, implying that similar to the recent developments in the protein world, the solution of RNA secondary structure prediction might be brought to us by the deep learning approaches. Additionally, we assessed the benefits of incorporating evolutionary information into the prediction of RNA secondary structures by testing SPOT-RNA2 [30] and comparing its prediction results to the aforementioned seven programs.

## Results

### Performance of various programs for RNA secondary structure prediction

We collected the published nucleotide sequences and the proposed or confirmed RNA secondary structures of 32 self-cleaving ribozyme sequences from the literature [32,51–58] (Table S1), which we referred to as the “native” sequences and structures below. These 32 sequences represent nine classes of self-cleaving ribozymes — hatchet, hepatitis delta virus (HDV), CPEB3, hammerhead (HHR), hovlinc, pistol, twister (TW), twister sister (TS), and Varkud satellite (VS). Although the biological functions of most of these nine classes of ribozymes are not fully understood, HHR, HDV, and VS ribozymes were found to be involved in the rolling circle replication of several kinds of circular RNA molecules [59]. HHR and HDV ribozymes were also found to be important for retrotransposition [59]. Additionally, several HHR and HDV ribozymes are thought to be function in the regulation of gene expression in both bacteria or eukaryotes [59]. The 32 ribozyme sequences include the two published versions of the hovlinc ribozyme: the fully active 168-nt Human_hovlinc and minimally active 83-nt Human_hovlinc_mini that retains ∼ 10% of its activity [32]. Among these ribozymes, six *trans*-acting ones (Pis_1, Pis_2, Pis_3, Pis_4, Hatch_1, and Hatch_2) were converted to *cis*-acting ribozymes by adding small loops connecting the enzyme and substrate parts (Table S1). The native secondary structures of these 32 ribozyme sequences are shown in Figure S1 and summarized in Table S2.

**Figure 1:**
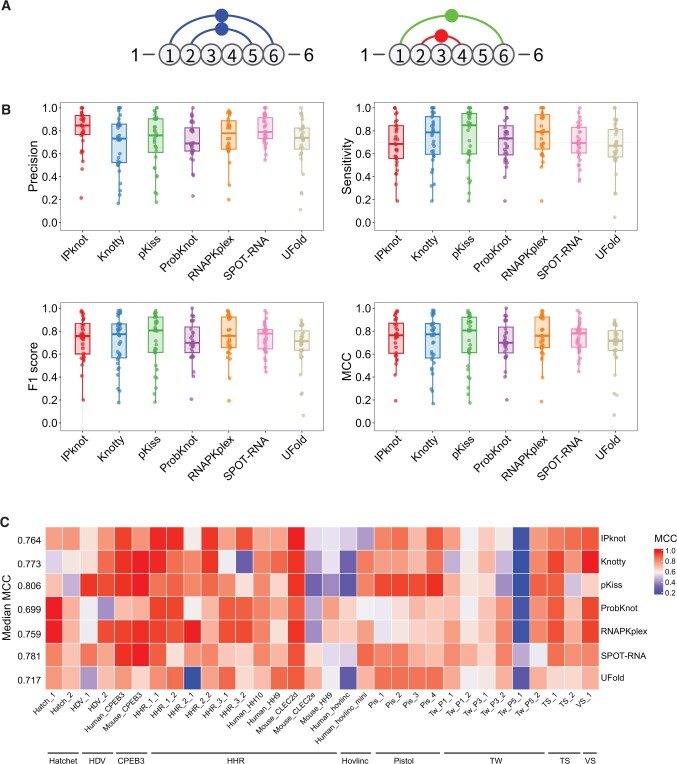
Illustration of the evaluation of the prediction and the performance of the seven programs **A**. Illustration of the evaluation of prediction. The reference (left panel) and predicted (right panel) structures of a 6-nt RNA sequence are represented by arc diagrams. In this prediction, the TP is base pair 1–6 (green in right panel) as it is present in both reference and predicted structures; the FN is base pair 2–5 as it is present only in the reference structure; the FP is base pair 2–4 (red in right panel) as it is present only in the predicted structure; and the TNs are base pairs 1–2, 1–3, 1–4, 1–5, 2–3, 2–6, 3–4, 3–5, 3–6, 4–5, 4–6, and 5–6 as they are absent in both reference and predicted structures (*i.e.*, all the possible base pairs *i*–*j*, *i*, *j* ∈ [1,6], *i* < *j*, with the exclusion of the TP, FN, and FP). Thus, for this prediction, the precision is 0.5, the sensitivity is 0.5, the F1 score is 0.5, and the MCC is ∼ 0.42. **B**. Box plots showing the precision, sensitivity, F1 score, and MCC across the 32 ribozyme sequences for each program. **C**. Heatmap showing the MCCs of the predictions obtained by each program for each ribozyme. The numbers on the left side of the heatmap are the median MCCs for each program. TP, true positive; FN, false negative; FP, false positive; TN, true negative; MCC, Matthews correlation coefficient; HHR, hammerhead; HDV, hepatitis delta virus; TW, twister; TS, twister sister; VS, Varkud satellite.

We then estimated the performance of the seven programs starting with the 32 native sequences of the self-cleaving ribozymes (Table S1) using four different metrics: precision, sensitivity, F1 score, and Matthews correlation coefficient (MCC) (see Materials and methods) (**Figure 1**A). Each metric measures different properties of an *in silico* RNA secondary structure prediction. Precision and sensitivity indicate the fraction of correctly predicted base pairings relative to, respectively, all predicted base pairs and those known to be true based on solved RNA structures. The F1 score is the harmonic mean of precision and sensitivity. On the other hand, while precision, sensitivity, and F1 score emphasize the correctly predicted base pairings (*i.e.*, the true positives), MCC is a more balanced measure [60,61].

Overall, all seven programs demonstrated good general performance as gauged by high median values (mostly in the range of ∼ 0.70–0.85) for all four metrics across all ribozymes (Figure 1B; **Table 1**). As shown in Table 1, pKiss showed the best overall performance in terms of median levels of sensitivity, F1 score, and MCC, while IPknot achieved the highest median precision. The best predictions for the 32 ribozymes are shown in Figure S2. Still, the performance of different programs on specific ribozymes or classes of ribozymes showed appreciable, and sometimes striking, variation (Figure 1C). Overall, the median difference between the maximum and minimum MCCs for the same ribozyme was ∼ 0.34 (Table S3). Out of the 32 ribozyme sequences, secondary structures of only two (Human_CPEB3 and Mouse_CLEC2d) or three (HHR_1_1, Mouse_CPEB3, and VS_1) could be predicted with MCC ≥ 0.9 by five or four programs, respectively, while none could be predicted by all seven programs (Figure 1C; Table S3). Secondary structure prediction for a member of the HHR class, HHR_3_2, represented the most striking example of the differences among the programs: IPknot, RNAPKplex, and ProbKnot yielded MCCs of ∼ 0.9; pKiss, UFold, and SPOT-RNA produced significantly lower MCCs of ∼ 0.61, ∼ 0.63, and ∼ 0.65, respectively, while the MCC of Knotty was only ∼ 0.28 (Figure 1C; Table S3). Altogether, these results indicate that the performance of the programs depends on some intrinsic properties of the native ribozyme secondary structures.

**Table 1:** Performance of the RNA secondary structure prediction programs on native ribozyme sequences

Program	Precision	Sensitivity	F1 score	MCC
IPknot	0.85	0.69	0.76	0.76
Knotty	0.73	0.79	0.77	0.77
pKiss	0.76	0.85	0.81	0.81
ProbKnot	0.69	0.73	0.70	0.70
RNAPKplex	0.78	0.79	0.76	0.76
SPOT-RNA	0.79	0.69	0.78	0.78
SPOT-RNA unseen data	0.79	0.68	0.77	0.77
UFold	0.74	0.67	0.71	0.72
SPOT-RNA2	0.75	0.84	0.79	0.79

*Note*: The median values based on the 32 ribozyme sequences are shown. MCC, Matthews correlation coefficient.

Thus, we investigated the correlation between the performance of RNA secondary structure prediction programs and several properties of the native ribozyme secondary structures, including the length of the sequence, the number of helices, the average length of helices, and the fraction of paired bases. As shown in Figure S3, with the exception of RNAPKplex, the performance of other programs did correlate significantly (*P* < 0.1) (Table S4) with different structural properties, as exemplified by the positive correlation with the average length of helices (Knotty and SPOT-RNA) or negative correlation with the number of helices (pKiss).

We also specifically investigated the ability of the programs to predict pseudoknots, a very common RNA structure element critical for the function of diverse classes of RNA molecules, including the RNA components of RNase P and telomerase RNA enzymes, ribosomal RNAs (rRNAs), and small nuclear RNAs (snRNAs) [42,62–66]. Pseudoknots are also critical for catalytic activities of many self-cleaving ribozymes, such as HDV [40,67], HHR [51], TW (54), pistol [53], and hovlinc [32], where they function by stabilizing the RNA tertiary structures and forming catalytic cores [41,42]. Despite their ubiquity and functional importance, pseudoknots are notoriously difficult to predict computationally due to their context sensitivity [43]. The non-nested base pairs forming the pseudoknots are usually undetectable by the RNA secondary structure prediction algorithms, like the widely used dynamic programming [43,68,69]. Therefore, pseudoknot prediction is out of the scope of many popular RNA secondary structure programs, such as Mfold [70] and RNAfold [45], and can only be achieved by several specially designed folding-based and machine learning-based programs, such as those tested in this work.

Thus, we evaluated how well the seven programs could predict the 29 pseudoknots involving 110 base pairs that are known to occur in 18 out of the 32 ribozyme secondary structures. The programs were evaluated based on three criteria: (1) correlation between MCCs and the numbers of bases involved in pseudoknots to evaluate the effects of pseudoknots on the overall program performance (Figure S3); (2) prediction of a correct pseudoknot by at least one base pair (**Table 2**); and (3) the fraction of correctly predicted base pairings in the pseudoknots (**Table 3**). In the third criterion, all pseudoknot base pairs were evaluated together since the number of pseudoknot base pairs from an individual ribozyme was too small for correct evaluation. As expected, all programs performed significantly worse in predicting pseudoknots compared to their performance in prediction of the overall RNA secondary structures (Tables 1–3), and the performance of the programs varied significantly. For example, the performance of IPknot, ProbKnot, and UFold, as measured by MCC, was negatively correlated with the number of base pairs involved in pseudoknots (Figure S3), suggesting that the performance of these programs is particularly affected by pseudoknots. Interestingly, based on the criteria 2 and 3, the deep learning-based program SPOT-RNA performed the best in terms of precision among the seven programs, while the folding-based program pKiss resulted in the highest sensitivity and F1 score. Nonetheless, the overall performance level of SPOT-RNA was either tied with or second to pKiss among the seven programs (Tables 2 and 3).

**Table 2:** Detection of pseudoknots by at least one base pair

Program	Precision	Sensitivity	F1 score
IPknot	0.11	0.07	0.08
Knotty	0.18	0.24	0.21
pKiss	0.32	0.41	0.36
ProbKnot	0.25	0.03	0.06
RNAPKplex	0.40	0.14	0.21
SPOT-RNA	0.43	0.31	0.36
UFold	0.10	0.10	0.10
SPOT-RNA2	0.53	0.28	0.36

**Table 3:** Detection of base pairs in pseudoknots

Program	Precision	Sensitivity	F1 score
IPknot	0.13	0.08	0.10
Knotty	0.22	0.35	0.27
pKiss	0.43	0.55	0.48
ProbKnot	0.23	0.03	0.05
RNAPKplex	0.44	0.25	0.32
SPOT-RNA	0.54	0.35	0.42
UFold	0.10	0.05	0.06
SPOT-RNA2	0.72	0.35	0.47

Curiously, all programs performed worst for the Tw_P5_1 ribozyme secondary structure prediction, while scoring much better on another ribozyme of the same subclass — Tw_P5_2 (Figure 1C; Table S3). The MCC for Tw_P5_1 obtained by SPOT-RNA was ∼ 0.45, while those from the other programs ranged between ∼ 0.17 and ∼ 0.24 (Table S3). Strikingly, unlike Tw_P5_2, Tw_P5_1 ribozyme’s secondary structure includes two non-canonical (not Watson–Crick and not G–U) base pairs (Figure S1), and prediction of non-canonical base pairs is out of the scope of these classical folding-based programs. However, SPOT-RNA was designed to handle such non-canonical base interactions because they were included in the training dataset for its machine learning algorithm [31]. Therefore, although the two non-canonical base pairs were not included in the final structure output by SPOT-RNA due to low probabilities (∼ 7.4E−10 and ∼ 0.003 for base pairs 2–36 and 10–30, respectively), they might have still benefitted the final SPOT-RNA prediction, resulting in a more accurate Tw_P5_1 secondary structure. As to another deep learning-based program UFold, although accepted as input, non-canonical base pairs were later excluded in the post-processing procedure that derived the secondary structure [50]. This might be the reason why the UFold prediction had the MCC of ∼ 0.24 for Tw_P5_1 ribozyme, which was lower than that generated by SPOT-RNA but higher than MCCs from all other folding-based programs. Although several other ribozymes also contain non-canonical base pairs in their structures, unlike Tw_P5_1 which has two non-canonical base pairs with one in the middle of a helix, they all have at most one such base pair which is always at the end of a helix (Figure S1). Therefore, for those ribozymes, the prediction of those non-canonical base pairs would not be critical for the prediction of the whole RNA secondary structure.

Overall, our benchmark efforts indicated that all the programs exhibited adequate overall performance. Their efficacy varied widely depending on the specific ribozyme, most likely due to different strengths and weaknesses in regards to specific properties of the RNA secondary structures. IPknot, ProbKnot, and UFold were poor at pseudoknot prediction, while pKiss suffered from predicting secondary structures containing many helices. Knotty and SPOT-RNA performed better when predicting long helices. The deep learning-based program SPOT-RNA showed comparable overall performance to the classical folding-based programs, meanwhile, it overperformed in prediction of secondary structures involving non-canonical base pairs. Importantly, this deep learning-based RNA secondary structure prediction program has reached and, in some ways, overperformed the top level of the classical folding-based programs in terms of the highly challenging pseudoknot prediction task, even though significant room for improvement remains.

A point of potential “unfairness” is that the deep learning-based program SPOT-RNA might have “seen” some of the ribozymes used in this work in its training process [31]. However, this program published in 2019 still gave very good performance on the hovlinc ribozyme which was discovered very recently by us in 2021 and whose functional RNA secondary structure was elucidated using compensatory mutagenesis approach and found to consist of four essential elements: helices S1 and S4, and pseudoknots pk_1 and pk_2 [32]. SPOT-RNA was the third and the best of the seven programs for predicting the RNA secondary structures of the full-length and minimal functional versions of the hovlinc ribozyme, respectively, in terms of MCC as illustrated in Figure 1C. Specifically, in the minimal hovlinc version, SPOT-RNA and Knotty captured all four structural elements. In contrast, pKiss, RNAPKplex, and UFold predicted three elements each (S1, S4, and pk_2 by pKiss and UFold; pk_1, pk_2, and S4 by RNAPKplex), the ProbKnot predicted two helices (S1 and S4), and the IPknot only captured the S4 helix (Figure S4).

To further evaluate the performance of SPOT-RNA, we excluded 9 ribozymes from the 32 ribozymes (Human_CPEB3, Mouse_CPEB3, Human_HH9, Mouse_HH9, Human_HH10, HDV_2, HHR_3_1, Tw_P1_1, and VS_1), because sequences with identity > 60% to these ribozymes were used to train that model. We found no drop in the SPOT-RNA performance on the remaining 23 sequences (Table 1, row “SPOT-RNA unseen data”). This further showed that the observed performance of SPOT-RNA was not biased by the presence of the sequences used to train this program.

### Evolutionary information does not significantly improve RNA secondary structure prediction

We then applied SPOT-RNA2 to these 32 ribozyme sequences to evaluate the potential improvement of RNA secondary structure prediction with the incorporation of evolutionary information (Figure S5; Table S5). The median values of the precision, sensitivity, F1 score, and MCC of the SPOT-RNA2 predictions were ∼ 0.75, ∼ 0.84, ∼ 0.79, and ∼ 0.79, respectively (Table 1). None of these values surpassed the highest levels achieved by the seven programs that did not utilize evolutionary information. In comparison with SPOT-RNA, the updated version SPOT-RNA2 improved in sensitivity (from ∼ 0.69 to ∼ 0.84), but showed no significant improvement in precision, F1 score, and MCC.

In terms of MCC on the individual ribozyme sequences, SPOT-RNA2 overperformed all the seven programs in the prediction of the RNA secondary structure of Hatch_2, performed worse than any of the seven programs in the prediction of the RNA secondary structure of Mouse_CLEC2d, and performed better than some while worse than others for the other 30 sequences (Figure S6). Notably, the most substantial drop in MCC occurred in the prediction for Human_hovlinc_mini (∼ 0.28 or ∼ 31%). This may be attributed to the artificial nature of Human_hovlinc_mini which was derived from the full-length 168-nt Human_hovlinc sequence by multiple deletions to represent the minimal functional form of the ribozyme [32], making it more challenging to obtain its evolutionary information. Compared with SPOT-RNA, SPOT-RNA2 resulted in increased, decreased, and identical MCCs for 16, 15, and 1 sequence, respectively (Figure S6).

In prediction of the pseudoknots in the secondary structures of the 32 ribozyme sequences, SPOT-RNA2 achieved precision, sensitivity, and F1 score of ∼ 0.53, ∼ 0.28, and ∼ 0.36, respectively, by the criterion 2 (Table 2), and ∼ 0.72, ∼ 0.35, and ∼ 0.47, respectively, by the criterion 3 (Table 3). In comparison with the seven programs, it improved in precision but not in sensitivity and F1 score. Overall, the results above indicate that the incorporation of the evolutionary information does not bring a significant improvement in predicting RNA secondary structures.

### The deep learning-based programs suffer the least from extended sequence context

RNA structure prediction programs frequently encounter sequences that contain not only the actual functional RNA but also some additional sequences at either 5′, 3′, or both ends. Such scenarios could include, for example, genome-wide computational screens for certain functional RNA species using sliding windows of genomic sequences, or synthetic biology and gene therapy applications where a ribozyme or some other functional RNAs have to act as a functional or regulatory element embedded in a longer sequence [51,71,72]. Therefore, the ability to correctly identify an RNA structural motif and/or to predict whether it could fold into an active structure within a longer sequence context has a considerable practical value.

**Figure 2:**
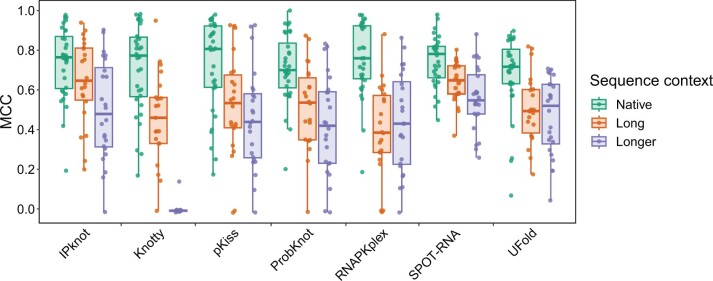
Box plots of MCCs for each program across all ribozymes active after addition of the extra sequences in the different sequence contexts

To investigate the effects of additional sequences on the self-cleavage activity and secondary structure prediction, we added 56–795 nt of additional flanking sequences to 31 of the 32 self-cleaving ribozyme sequences (except for the Human_hovlinc_mini), resulting in extensions ranging from 33.5% to 597.0%. The additional sequences were either randomly generated or represented the cognate sequences from the corresponding genomes (see Materials and methods for details). We confirmed the self-cleavage activity of 46 variants (see Materials and methods) and further stratified them into “long” and “longer” categories based on the lengths of the final sequences (Figure S7): the “long” variants were 183 nt in length with the exception of the VS_1 variant (223 nt), while the “longer” ones were 362–498 nt with the exception of the Mouse_CLEC2e variant (942 nt). We then employed these active extended variants to assess the performance of the RNA secondary structure prediction programs. As shown in **Figure 2**, the addition of the extra sequences resulted in a drop in the performance of all the programs. The trend was consistent across all the tested ribozyme sequences (Figure S8). SPOT-RNA and UFold showed the smallest decreases in the performance. The median MCCs for native, “long”, and “longer” sequences were ∼ 0.78, ∼ 0.65, and ∼ 0.55, respectively, for SPOT-RNA, and were ∼ 0.72, ∼ 0.49, and ∼ 0.52, respectively, for UFold. Knotty showed the largest decrease (median MCCs for the native, “long”, and “longer” sequences were ∼ 0.77, ∼ 0.46, and ∼ 0.009, respectively). Overall, Knotty performed the worst in this test, especially for ribozymes in the “longer” sequence context, and it even failed in the prediction of one “long” and five “longer” sequences.

These results highlight the challenge of accurately predicting RNA secondary structures even when a relatively small additional sequence is added to each ribozyme as compared to what would be expected if, for example, a ribozyme is placed in a context of a much larger messenger RNA (mRNA). The results also indicate that the performance of SPOT-RNA and UFold suffers the least from the additional sequence context and this might be another advantage of the deep learning-based methods.

### Sensitivity to detect small functionally relevant changes in RNA structures

Recently developed high-throughput methods based on combination of mutagenesis and next-generation sequencing resulted in valuable datasets that measure effects of sequence variants on activities for multiple ribozymes [34,35]. We took advantage of two such deep mutational scanning datasets to assess the effects of mostly small perturbations of the ribozyme structures on the performance of the RNA secondary structure prediction programs. The first dataset was based on the study by Zhang et al. that generated 18,307 mutants containing one or more mutations in the human 81-nt CPEB3 self-cleaving ribozyme (Figure S9A) resulting in relative activities (RAs) that ranged from 0.5 to 1.73, with 1 being equal to the activity of the wild-type sequence [34]. The second dataset was based on the study by Kobori and Yokobayashi that generated 20,535 mutants for a 54-nt TW ribozyme from *Oryza sativa* (Figure S9B) resulting in RAs ranging from 0.5 to 1.22 [35].

**Figure 3:**
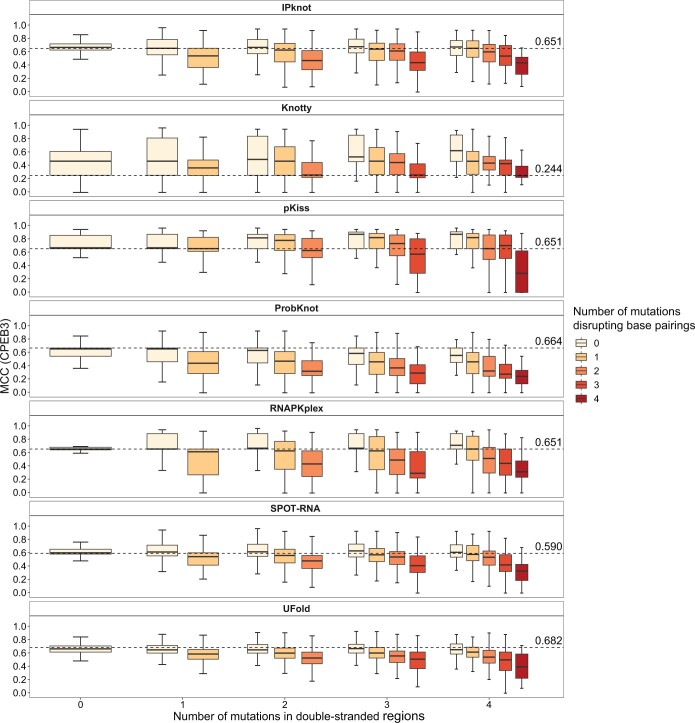
Effects of mutations disrupting or not disrupting base pairings in double-stranded regions on RNA structure predictions Box plots of MCCs (Y-axes) are shown for the mutants that contain variable number of mutations (X-axes) that belong to the indicated categories (the inset on the right) in CPEB3 ribozyme. Mutants with > 4 mutations in the CPEB3 dataset were excluded from the analysis, because the number was too small. The dashed lines with corresponding values on them show the levels of the wild-type ribozyme sequences.

**Figure 4:**
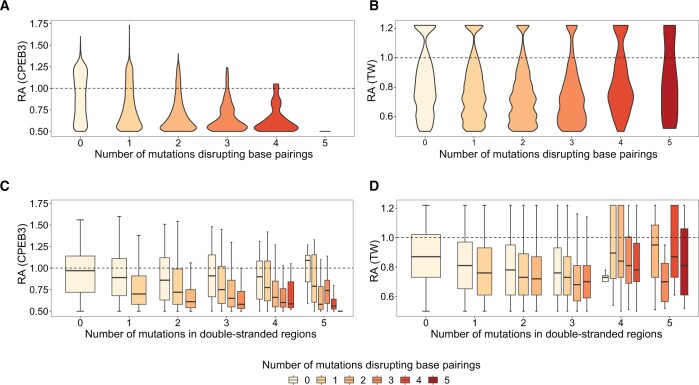
Effects of mutations disrupting or not disrupting base pairings in double-stranded regions on RAs Violin plots of RAs (Y-axes) are shown for mutants that contain variable number of mutations (X-axes) disrupting base pairings in the RNA structures of the CPEB3 (**A**) and TW (**B**) ribozymes. Box plots of RAs (Y-axes) are shown for mutants that contain variable number of total mutations in double-stranded regions (X-axes) that belong to the indicated categories (insets at the bottom) in the CPEB3 (**C**) and TW (**D**) ribozymes. RA, relative activity.

It’s worth to note that not all the mutations might influence the self-cleavage activity by disrupting the RNA secondary structure. Some mutations, such as those substituting the general base or acid of the self-cleaving reaction, could abolish the reaction without disrupting the ribozyme’s secondary structure [39,41]. Therefore, prior to the assessment, we investigated whether we could correlate changes in RNA secondary structure of the ribozymes to changes in their RAs. First, we found that single mutations that preserved base pairings did not significantly affect the RAs (Figure S10). Therefore, the MCCs of these mutants from all the programs should be very close to the levels of predictions based on the wild-type ribozyme sequences, which was indeed the case (Figure S10). On the other hand, mutations that disrupted the base pairings would be expected to affect RNA secondary structure the most, and this was indeed observed as shown by the general decrease of MCC with the increase in the number of such mutations per ribozyme (**Figure 3**, Figure S11). We then tested whether the number of base pairing disrupting mutations inversely correlated with RA, and indeed found such a trend for both ribozymes (**Figure 4**A and B). Furthermore, the decrease in RA was directly related to the number of mutations that disrupted the base pairings, since mutations in double-stranded regions that preserved the base pairing (*i.e.*, single mutation changing a Watson–Crick base pair to a G–U pair or *vice versa*, and compensatory mutations) did not lead to decrease in RA (Figure 4C and D). These results indicate that the RA could serve as a metric to evaluate changes of the RNA secondary structures in the mutants.

**Figure 5:**
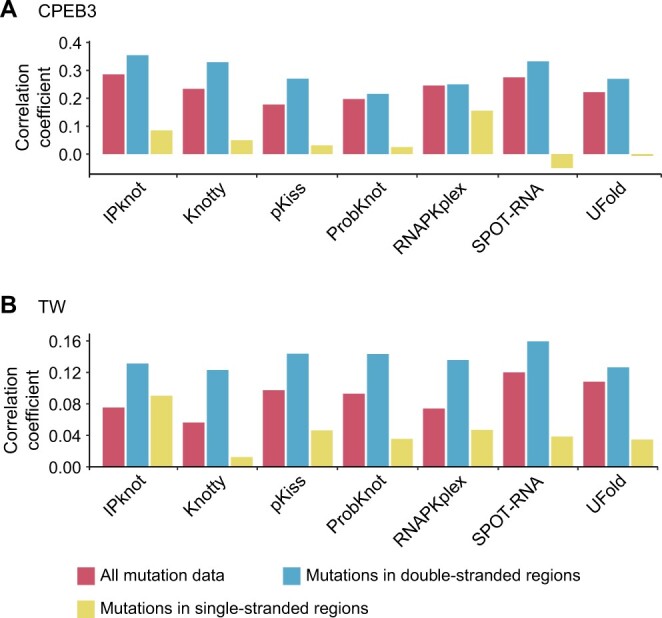
Correlation between the MCCs of the predicted structures and the corresponding RAs for ribozyme mutants The correlation coefficients (Y-axes) from the CPEB3 (**A**) and TW (**B**) datasets were both higher in mutants with mutations confined to double-stranded regions compared to all mutants, and became significantly lower or even negative for mutants that contained mutations in single-stranded regions.

As shown in **Figure 5** and Figures S12 and S13, for all seven programs, the MCCs of the predicted secondary structures of the mutants correlated with the corresponding RAs for both ribozymes. As expected, the correlations were stronger for mutations located only in the double-stranded regions and disappeared or became very weak for mutations confined to the single-stranded regions (Figure 5, Figures S14–S17). These results indicate that the RNA secondary structure prediction programs are sensitive enough to capture the relatively small effects of mutations on both RNA secondary structure and function. Overall, the deep learning-based SPOT-RNA performed quite well being the second-best and the best for the CPEB3 and TW datasets, respectively, when considering only mutations in the double-stranded regions (Figure 5).

**Figure 6:**
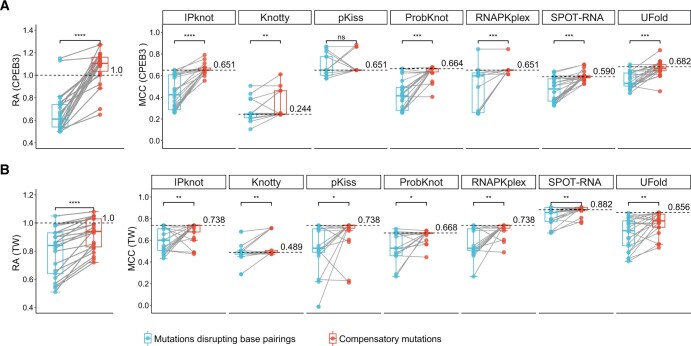
Compensatory mutagenesis analysis Changes in RAs (left panels, Y-axes) and MCCs (right panels, Y-axes) between the pairs of mutants with single mutations disrupting base pairings (blue circles) and corresponding compensatory mutations (red circles) are shown for the CPEB3 (**A**) and TW (**B**) datasets. The dashed lines with corresponding values on them show the levels of the wild-type ribozyme sequences. The statistical significances from two-sides paired Mann–Whitney–Wilcoxon tests are shown above the connecting lines (*, *P* < 0.05; **, *P* < 0.01; ***, *P* < 0.001; ****, *P* < 0.0001; ns, not significant).

To further test the ability of the different programs to correctly gauge small functionally relevant changes in secondary structure, we focused on two types of sequence changes: type I, single mutations that disrupt base pairings, and type II, the corresponding compensatory mutations that recover the disrupted base pairings. In such cases, the single mutations should disrupt the secondary structure and reduce the RA, while the corresponding compensatory mutations introduce sequence changes in the background of the single mutations that should recover the disrupted secondary structure and therefore, also recover the activity [34]. We identified 23 and 30 pairs of single and compensatory mutations in the CPEB3 and TW datasets, respectively. As expected, in 22 and 21 pairs from the CPEB3 and TW datasets, respectively, the type I mutations reduced the RA that was then restored, at least partially, by the type II mutations (**Figure 6**). Most of the programs, IPknot, ProbKnot, RNAPKplex, SPOT-RNA, and UFold, could reproduce the patterns of the RA: the MCC values were reduced by the type I mutations and at least partially recovered by the type II mutations (Figure 6). Such pattern was not observed for Knotty in both datasets, and for pKiss in the CPEB3 dataset.

We calculated the changes in the RAs (ΔRA) and MCCs (ΔMCC) between the corresponding pairs of single and compensatory mutants (see Materials and methods). We then calculated the correlations between ΔRA and ΔMCC for each program across the 22 and 21 pairs of CPEB3 and TW mutants, respectively. Ideally, ΔRA and ΔMCC should exhibit significant positive correlations. As shown in Figure S18, this correlation was observed only in SPOT-RNA. Investigations using F1 score instead of MCC revealed essentially the same results (Figure S19). These results demonstrate that while most, albeit not all, of the programs are sufficiently sensitive to detect the small yet functionally relevant perturbations of the single and compensatory mutations on the RNA secondary structure, SPOT-RNA appears to be the most sensitive one. To further confirm the sensitivity of SPOT-RNA, we also checked the correlations between ΔRA and the changes of the SPOT-RNA-predicted base pairing probabilities between the corresponding pairs of single and compensatory mutants, and observed similar results (Figure S20).

## Discussion

In this study, we investigated the performance of seven RNA secondary structure prediction programs, including five classical folding-based and two deep learning-based programs, using characterized secondary structures of self-cleaving ribozymes as benchmarks. It is important to mention that our results and conclusions are based on a specific class of functional RNAs and therefore their applicability to other functional structured RNAs requires further investigation.

The five classical programs attempt to find the best RNA structures based on various implementations of the conventional folding-based approaches using a wide range of algorithms. Knotty and RNAPKplex are based on dynamic programming. Knotty solves the minimum free energy (MFE) structure [47], and RNAPKplex is an “intramolecular variant” of RNA–RNA interaction prediction which first computes the accessibilities of the sequence and then identities regions forming pseudoknots [45]. IPknot and ProbKnot identify the maximum expected accuracy (MEA) structure; and while IPknot solves the MEA structure by integer programming [48], ProbKnot first predicts base pairing probabilities using a partition function and then assembles the MEA structure from the probabilities [44]. Additionally, pKiss achieves predictions using abstract shape analysis of pseudoknot structures [46]. In the deep learning-based program SPOT-RNA, an ensemble model of residual networks (ResNets) and long short-term memory (LSTM), is first trained on bpRNA dataset and then fine-tuned on the high-resolution experimental RNA structures from the Protein Data Bank (PDB) using transfer learning technique [31]. Another deep learning-based program UFold first converts the RNA sequence into an image-like representation and then uses a fully convolutional network U-Net model to predict the structure [50]. Many of the programs have been reviewed by Bugnon and colleagues [73].

Other deep learning-based programs have been developed recently. For example, Townshend et al. published a deep learning-based open source method named Atomic Rotationally Equivariant Scorer (ARES) achieving highly accurate predictions of tertiary RNA structures at atom level [74], and Singh et al. published SPOT-RNA2, an improved version of SPOT-RNA that incorporates evolutionary conservation analysis [30]. However, ARES is a scoring algorithm relying on other methods to produce RNA structure for scoring [74] and thus is not suitable for the comparison performed in this work. SPOT-RNA2, as discussed above, needs evolutionary information in addition to the nucleotide sequence for prediction, and thus cannot be used to detect newly evolved functional RNAs [30]. Also, in our assessment, the incorporation of evolutionary information in SPOT-RNA2 does not improve the prediction of RNA secondary structures significantly. Additionally, in 2021, Sato et al. published MXfold2 [75] and Wang et al. published RPRes [76]. These two programs were also excluded from this study due to the lack of the pseudoknot prediction capability and a ready-to-use model for the end user, respectively [75,76].

We characterized performance of the programs in four different applications based on the reported sequence of each ribozyme: (1) overall prediction of the secondary structure; (2) prediction of pseudoknots; (3) prediction of RNA secondary structure in an expanded sequence context; and (4) ability to detect relatively small perturbations in secondary structure and function. While showing some notable differences in the performance on individual ribozymes, on average, all programs have achieved high degree of concordance with the characterized secondary structures when using the reported sequences as input. Interestingly, in this task, a folding-based program pKiss slightly overperformed all other programs in terms of sensitivity, F1 score, and MCC. On the other hand, the performance of the programs has shown significant variation in more challenging tasks. For example, the programs differed significantly, and none performed perfectly, in the pseudoknot prediction challenge. Interestingly, the deep learning-based program has shown adequate, if not the best, performance in all challenging tasks, while the classical folding-based programs performed well only in some of them. For example, pKiss and SPOT-RNA were the top 2 programs in terms of the pseudoknot prediction; however, unlike SPOT-RNA, pKiss failed to capture the relatively small effects of the perturbations on RNA secondary structures caused by sequence changes. SPOT-RNA overperformed the folding-based programs when predicting ribozyme secondary structures involving non-canonical base pairs and in terms of the ability to detect small effects on secondary structure and function caused by sequence changes.

Additionally, the performance of all the classical folding-based programs, especially Knotty, dropped dramatically when the ribozyme sequences were embedded in larger sequence contexts. This is a known limitation of the classical folding-based RNA secondary structure prediction programs — the rapidly enlarged searching space due to the increase of the length of the RNA molecule hampers the programs from finding the correct base pairings [22]. Indeed, it has been shown for the full-length mRNAs, the folding-based programs cannot capture the RNA folding pathway derived from the experimentally probed structures [77]. This limitation hinders the application of the classical folding-based programs in some scenarios, such as analysis of the full-length mRNAs and lncRNAs, and when designing long regulatory RNA molecules with embedded short elements. The existence of regulatory RNA structural elements in the untranslated regions (UTRs) and coding regions of mRNAs [77,78], as well as our recent finding of the hovlinc ribozyme in a human lncRNA which demonstrates that the lncRNA might function by carrying catalytic RNA domains [32], emphasizes the importance of predicting RNA structural elements within long RNA molecules. In this respect, the deep learning-based approach might be a solution to this problem, since it could circumvent the expanded searching space problem. Indeed, our results indicate that the deep learning-based programs SPOT-RNA and UFold significantly overperform the classical folding-based programs in prediction of the ribozyme secondary structures embedded in larger sequence contexts.

However, it is worth noting that there is still a lot of room for improvement since the performance of deep learning based-programs, especially UFold, was still far from perfect in terms of the pseudoknot prediction and they also suffered, albeit to a much lesser degree, when the sequences of interest were embedded within larger sequence contexts. Still, combined with other features of SPOT-RNA such as prediction of lone base pairs, base triplets, and long-distance interactions, our study suggests that the SPOT-RNA program and the deep learning-based strategy in general have great potential in the area of RNA secondary structure prediction. In fact, they might give the field the conceptual leap forward in the same way that the AlphaFold algorithm propelled the protein-folding prediction field [79]. However, the dramatic differences between the two deep learning-based programs, SPOT-RNA and UFold, in some of the assays indicate that careful design of the deep learning architecture and selection of the datasets are necessary to achieve this potential.

In summary, based on our assessment (Table S6), we would like to make the following suggestions to the end users: (1) for folding short sequences (< ∼ 200 nt), all the seven programs can be used; (2) for identifying RNA structural elements within larger sequence contexts, the two deep learning-based programs SPOT-RNA and UFold would be better choices compared to other folding-based programs, and Knotty should be avoided; (3) if finding pseudoknots is critical, pKiss or SPOT-RNA would be preferable; (4) if non-canonical base pairs might be involved in the structures, SPOT-RNA should be used; and (5) if the study is to compare the effects of single and the corresponding compensatory mutations, SPOT-RNA would be the suitable program to use.

## Materials and methods

### RNA secondary structure prediction programs

The standalone versions of the RNA secondary structure prediction programs were downloaded from their respective official websites or GitHub (https://github.com) repositories as follows: IPknot (v1.0.0) [48,49], Knotty (git commit 9e8eab1) [47], pKiss (v2.2.14) [46], ProbKnot (v6.1) [44], RNAPKplex (v2.4.18) [45], SPOT-RNA (git commit 3a7a271) [31], UFold (git commit 6af7194) [50], and SPOT-RNA2 (git commit 2247afa). The default parameters of each program were used in the RNA secondary structure prediction, and for pKiss, the “mfe” mode was used. For SPOT-RNA and SPOT-RNA2, in each predicted base triplets (one base pairing with two other bases) only the base pair with the highest base pairing probability was included in the analysis. In very rare cases, RNAPKplex outputted more than one structure with identical free energy, and in such cases, the first structure was taken as the final prediction. For SPOT-RNA2, the nt database was downloaded from NCBI on December 1, 2023.

### Performance evaluation of RNA secondary structure prediction programs

For a given ribozyme sequence or its mutant, the predicted RNA secondary structures from the programs were compared with the native structure of the ribozyme. Note that for a ribozyme within a larger sequence context, all predicted base pairs involving nucleotides outside the embedded ribozyme segments were excluded from the comparison. The performance of the RNA secondary structure prediction programs was evaluated by the resulting precision, sensitivity, F1 score, and MCC.

The processed data, *i.e.*, the sequences and RAs of the mutants, from the deep mutational scanning datasets for the CPEB3 [34] and TW [35] ribozymes were downloaded from https://github.com/zh3zh/CODA. For the compensatory mutagenesis analysis, ΔRA or ΔMCC was calculated as the RA or MCC of a single mutant subtracted from the RA or MCC of the corresponding compensatory mutant. The RNA secondary structures were plotted using VARNA software [80].

### Synthesis of ribozymes

The “long” and “longer” variants of ribozymes were obtained by *in vitro* transcription (IVT). The DNA templates of IVT were synthesized by: (1) 1-round polymerase chain reaction (PCR) from annealed pairs of overlapping synthetic oligonucleotides essentially as described previously [32] (for all the “long” variants of ribozymes; randomly generated sequences with ∼ 50% GC content were attached to the 5′ and/or 3′ ends of the native ribozyme sequences); (2) 1-round PCR from genomic DNA (for the “longer” variants of Human_HH9, Human_HH10, Human_hovlinc, Human_CPEB3, Mouse_HH9, Mouse_CLEC2d, Mouse_CLEC2e, and Mouse_CPEB3; the cognate sequences from the corresponding genomes were attached to the native ribozyme sequences); or (3) 2-round PCR from the synthesized DNA templates of the corresponding “long” variants (for the “longer” variants of all other ribozymes; additional randomly generated sequences with ∼ 50% GC content were attached to the 5′ and/or 3′ ends of the “long” variants). The details of the PCRs and sequences of the oligos and primers are listed in Table S7. A T7 promoter was added to the 5′ end of the oligo representing the sense strand of RNA or the corresponding primer of PCR (Table S7). The IVT reactions were performed in the buffer containing 40 mM 2-(*N*-morpholino)ethanesulfonic acid (MES) pH 6.5, 6 mM MgCl_2_, 2 mM spermidine, 1 mM dithiothreitol (DTT), 1.5 mM of each adenosine triphosphate (ATP), uridine triphosphate (UTP), guanosine triphosphate (GTP), and cytidine triphosphate (CTP), 5 U/µl of T7 RNA polymerase (Catalog No. M0251, New England Biolabs, Ipswich, MA) and 10 ng/µl DNA template at 37°C for 2 h. Then, the uncleaved IVT products were purified on 15% denaturing polyacrylamide gel electrophoresis (PAGE) gels containing 6 M urea (Catalog No. E301U6F, WSHTBio, Shanghai, China).

### Cleavage assays

The PAGE-purified IVT products were first denaturized and renatured in 150 mM KCl and 30 mM Tris-HCl (pH 8.0). Then, the cleavage was initiated by adding MgCl_2_ to the final concentration of 6 mM, and the reactions were incubated at 37°C for 1 h in a total volume of 10 µl. Meanwhile, parallel control reactions were incubated with 6 mM ethylenediaminetetraacetic acid (EDTA) instead of MgCl_2_. Then, the reactions were quenched by adding 1 volume of RNA loading buffer containing 88% formamide, 50 mM EDTA, 0.02% xylene cyanol, and 0.02% bromophenol blue, and the cleavage products of “long” and “longer” sequences were separated, respectively, by 15% and 6% denaturing PAGE gels containing 6 M urea. The self-cleavage activity of a sequence would be confirmed when the expected bands of the cleavage products were observed in the gels.

## CRediT author statement

**Fei Qi:** Conceptualization, Methodology, Validation, Investigation, Visualization, Writing – original draft, Writing – review & editing, Supervision, Project administration. **Junjie Chen:** Software, Formal analysis, Data curation, Visualization. **Yue Chen:** Investigation, Writing – original draft, Writing – review & editing. **Jianfeng Sun:** Methodology, Writing – original draft, Writing – review & editing. **Yiting Lin:** Investigation. **Zipeng Chen:** Investigation. **Philipp Kapranov:** Supervision, Writing – original draft, Writing – review & editing. All authors have read and approved the final manuscript.

## Supplementary material

Supplementary material is available at *Genomics, Proteomics & Bioinformatics* online (https://doi.org/10.1093/gpbjnl/qzae043).

## Competing interests

The authors have declared no competing interests.

## Supplementary Material

qzae043_Supplementary_Data
