# Supplementary material for: Evaluating Performance of Different RNA Secondary Structure Prediction Programs Using Self-cleaving Ribozymes
Source: Genomics Proteomics Bioinformatics. 2024 Jun 8;22(3):qzae043. doi: 10.1093/gpbjnl/qzae043 (PMC12016570; doi:10.1093/gpbjnl/qzae043)
Supplement: qzae043_Supplementary_Data [file qzae043_supplementary_data.zip › Figure_S7.pdf]

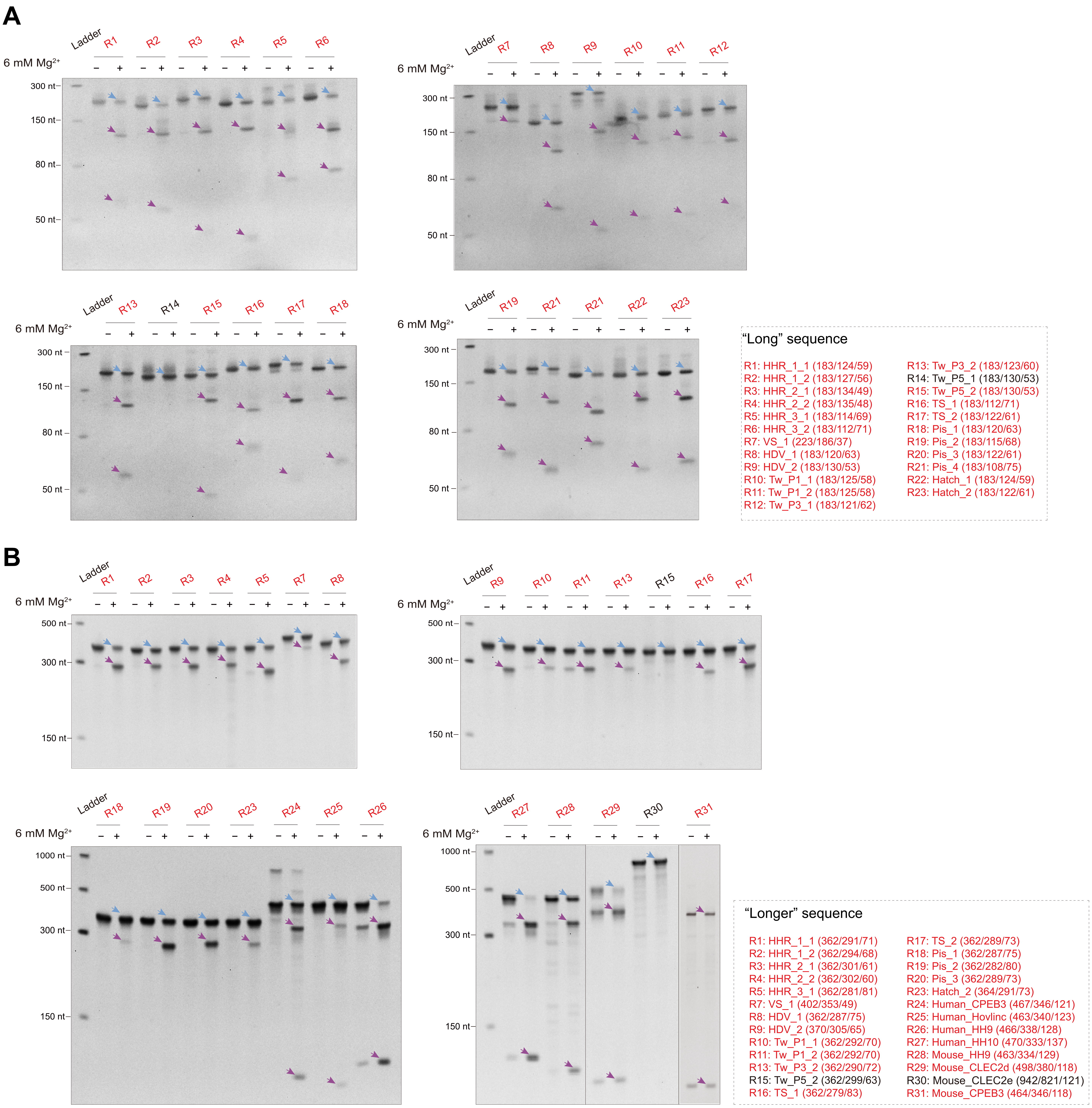

A
Ladder
R1 R2 R3 R4 R5 R6
6 mM Mg2+
– + – + – + – + – + – +
300 nt
150 nt
80 nt
50 nt
Ladder
R7 R8 R9 R10 R11 R12
6 mM Mg2+
300 nt
150 nt
80 nt
50 nt
Ladder
R13 R14 R15 R16 R17 R18
6 mM Mg2+
300 nt
150 nt
80 nt
50 nt
Ladder
R19 R21 R21 R22 R23
6 mM Mg2+
300 nt
150 nt
80 nt
50 nt
"Long" sequence
R1: HHR_1_1 (183/124/59)
R2: HHR_1_2 (183/127/56)
R3: HHR_2_1 (183/134/49)
R4: HHR_2_2 (183/135/48)
R5: HHR_3_1 (183/114/69)
R6: HHR_3_2 (183/112/71)
R7: VS_1 (223/186/37)
R8: HDV_1 (183/120/63)
R9: HDV_2 (183/130/53)
R10: Tw_P1_1 (183/125/58)
R11: Tw_P1_2 (183/125/58)
R12: Tw_P3_1 (183/121/62)
R13: Tw_P3_2 (183/123/60)
R14: Tw_P5_1 (183/130/53)
R15: Tw_P5_2 (183/130/53)
R16: TS_1 (183/112/71)
R17: TS_2 (183/122/61)
R18: Pis_1 (183/120/63)
R19: Pis_2 (183/115/68)
R20: Pis_3 (183/122/61)
R21: Pis_4 (183/108/75)
R22: Hatch_1 (183/124/59)
R23: Hatch_2 (183/122/61)
B
Ladder
R1 R2 R3 R4 R5 R7 R8
6 mM Mg2+
500 nt
300 nt
150 nt
Ladder
R9 R10 R11 R13 R15 R16 R17
6 mM Mg2+
500 nt
300 nt
150 nt
Ladder
R18 R19 R20 R23 R24 R25 R26
6 mM Mg2+
1000 nt
500 nt
300 nt
150 nt
Ladder
R27 R28 R29 R30 R31
6 mM Mg2+
1000 nt
500 nt
300 nt
150 nt
"Longer" sequence
R1: HHR_1_1 (362/291/71)
R2: HHR_1_2 (362/294/68)
R3: HHR_2_1 (362/301/61)
R4: HHR_2_2 (362/302/60)
R5: HHR_3_1 (362/281/81)
R7: VS_1 (402/353/49)
R8: HDV_1 (362/287/75)
R9: HDV_2 (370/305/65)
R10: Tw_P1_1 (362/292/70)
R11: Tw_P1_2 (362/292/70)
R13: Tw_P3_2 (362/290/72)
R15: Tw_P5_2 (362/299/63)
R16: TS_1 (362/279/83)
R17: TS_2 (362/289/73)
R18: Pis_1 (362/287/75)
R19: Pis_2 (362/282/80)
R20: Pis_3 (362/289/73)
R23: Hatch_2 (364/291/73)
R24: Human_CPEB3 (467/346/121)
R25: Human_Hovlinc (463/340/123)
R26: Human_HH9 (466/338/128)
R27: Human_HH10 (470/333/137)
R28: Mouse_HH9 (463/334/129)
R29: Mouse_CLEC2d (498/380/118)
R30: Mouse_CLEC2e (942/821/121)
R31: Mouse_CPEB3 (464/346/118)
