# Supplementary material for: Evaluating Performance of Different RNA Secondary Structure Prediction Programs Using Self-cleaving Ribozymes
Source: Genomics Proteomics Bioinformatics. 2024 Jun 8;22(3):qzae043. doi: 10.1093/gpbjnl/qzae043 (PMC12016570; doi:10.1093/gpbjnl/qzae043)
Supplement: qzae043_Supplementary_Data [file qzae043_supplementary_data.zip › Supplementary material captions.docx]

# Supplementary material

**Figure S1** **Native RNA secondary structures of the ribozymes**

The red triangles indicate the cleavage sites, and the non-canonical base pairs are marked in magenta.

**Figure S2** **The best predictions for the RNA secondary structures of the ribozymes**

The programs produced the best predictions are shown in the parentheses, and the non-canonical base pairs are marked in magenta.

**Figure S3** **Correlations between the MCCs of the predictions and the indicated intrinsic structural properties of the ribozymes**

The Pearson correlation coefficients and *P* values of significant correlations are shown.

**Figure S4** **The predicted structures of the minimal function version (83 nt) hovlinc ribozyme from the 7 programs**

**A.** SPOT-RNA. **B.** Knotty. **C.** pKiss. **D.** ProbKnot. **E.** UFold. **F.** RNAPKplex. **G.** IPknot. The captured essential elements are marked.

**Figure S5** **The predictions for the RNA secondary structures of the ribozymes by SPOT-RNA2**

The non-canonical base pairs are marked in magenta.

**Figure S6** **The MCCs of the predictions from SPOT-RNA2 compared with the 7 programs**

The dashed lines indicate the MCCs of the predictions from SPOT-RNA2.

**Figure S7** **Self-cleavage activities of the ribozymes in extended sequence context**

**A.** The “long” ribozyme sequences. **B.** The “longer” ribozyme sequences. The cleavage reactions were performed either in the presence of 6 mM Mg^2+^ (+) or 6 mM EDTA as control (−). Blue and purple arrowheads indicate uncleaved transcripts and cleavage products, respectively. Some cleavage products were too small to be in the range of the gels shown here. Numbers in the parentheses represent the expected lengths (nt) of uncleaved transcripts, downstream and upstream cleavage products, respectively, from left to right. Sequences whose activities were confirmed by these assays are marked in red. EDTA, ethylenediaminetetraacetic acid.

**Figure S8** **Effect of additional sequences on the performance of the structure prediction programs**

Boxplots of MCCs (Y-axis) for each ribozyme calculated based on its native sequence alone or embedded in the “long” or “longer” sequence contexts. Only the ribozymes active in multiple sequence contexts are shown. The numbers on top of the boxplots of the native sequences indicate their lengths (nt), while the numbers on top of the boxplots of the “long” and “longer” sequences indicate the numbers of additional nucleotides added to their 5' (upper) and 3' (lower) ends.

**Figure S9** **The actual sequences of the ribozymes used in the deep mutational scanning studies and the structures of these ribozymes**

**A.** The CPEB3 ribozyme. **B.** The twister ribozyme. The red triangles indicate the cleavage sites for both ribozymes, and the green arrows indicate the boundaries of the minimal human CPEB3 ribozyme. The minimal native sequence of the twister ribozyme was used in the mutation scan. The non-canonical base pairs are marked in magenta.

**Figure S10** **Effects of single mutations preserving base pairing on relative activities**

The RAs (left, Y-axes) and the MCCs from all the programs (right, Y-axes) were very close to the corresponding levels of the wild-type ribozyme sequences for the CPEB3 dataset (**A**) and the twister dataset (**B**). The dashed lines and the numbers show the levels for the wild-type ribozyme sequences.

**Figure S11** **Effects of mutations in double-stranded regions that do and do not disrupt base pairings on RNA structure predictions**

Box plots of MCCs (Y-axes) are shown for the mutants that contain variable number of mutations (X-axes) that belong to the indicated categories (the inset on the right) in twister ribozyme. Mutants > 3 mutations in the twister dataset were excluded from the analysis because the number was too few.

**Figure S12** **Correlations between the RAs and the MCCs for all mutants of the CPEB3 dataset**

The Pearson correlation coefficients and *P* values are shown.

**Figure S13** **Correlations between the RAs and the MCCs for all mutants of the twister dataset**

The Pearson correlation coefficients and *P* values are shown.

**Figure S14** **Correlations between the RAs and the MCCs for mutants containing mutations only in double-stranded regions of the CPEB3 dataset**

The Pearson correlation coefficients and *P* values are shown.

**Figure S15** **Correlations between the RAs and the MCCs for mutants containing mutations only in single-stranded regions of the CPEB3 dataset**

The Pearson correlation coefficients and *P* values are shown.

**Figure S16** **Correlations between the RAs and the MCCs for mutants containing mutations only in double-stranded regions of the twister dataset**

The Pearson correlation coefficients and *P* values are shown.

**Figure S17** **Correlations between the RAs and the MCCs for mutants containing mutations only in single-stranded regions of the twister dataset**

The Pearson correlation coefficients and *P* values are shown.

**Figure S18** **Correlations between the ΔRA and ΔMCCs of the pairs of single and corresponding compensatory mutants**

**A.** The CPEB3 dataset. **B.** The twister dataset. The Pearson correlation coefficients and *P* values are shown.

**Figure S19** **Correlations between the ΔRA and ΔF1_scores of the pairs of single and corresponding compensatory mutants**

**A.** The CPEB3 dataset. **B.** The twister dataset. The Pearson correlation coefficients and *P* values are shown.

**Figure S20** **Correlations between the ΔRA and changes of the SPOT-RNA-predicted base pairing probabilities of the pairs of single and corresponding compensatory mutants**

**A.** The CPEB3 dataset. **B.** The twister dataset. The Pearson correlation coefficients and *P* values are shown. The absolute values of the ΔRMSD (calculated between the base pairing probabilities of the single or the compensatory mutants and the native structure of the original sequence; shown in Y-axes) represented the changes of the SPOT-RNA-predicted base pairing probabilities. RMSD, root mean square distance.

**Table S1** **Information of the 32 native ribozymes sequences, and their corresponding “long” and “longer” sequences**

**Table S2** **Summary of the RNA secondary structures of the ribozymes**

**Table S3** **Predictions metrics of the 32 native ribozyme sequences from the 7 RNA secondary structure programs**

**Table S4** **Correlations between the MCCs of predictions and several structural properties of the ribozymes**

**Table S5 Predictions metrics of the 32 native ribozyme sequences from SPOT-RNA2**

**Table S6 Summary of the results of the assessment**

**Table S7 Details of the PCRs and sequences of the primers for synthesis of IVT DNA templates for obtaining the ribozymes**
