# Supplementary material for: Evaluating Performance of Different RNA Secondary Structure Prediction Programs Using Self-cleaving Ribozymes
Source: Genomics Proteomics Bioinformatics. 2024 Jun 8;22(3):qzae043. doi: 10.1093/gpbjnl/qzae043 (PMC12016570; doi:10.1093/gpbjnl/qzae043)
Supplement: qzae043_Supplementary_Data [file qzae043_supplementary_data.zip › Figure_S2.pdf]

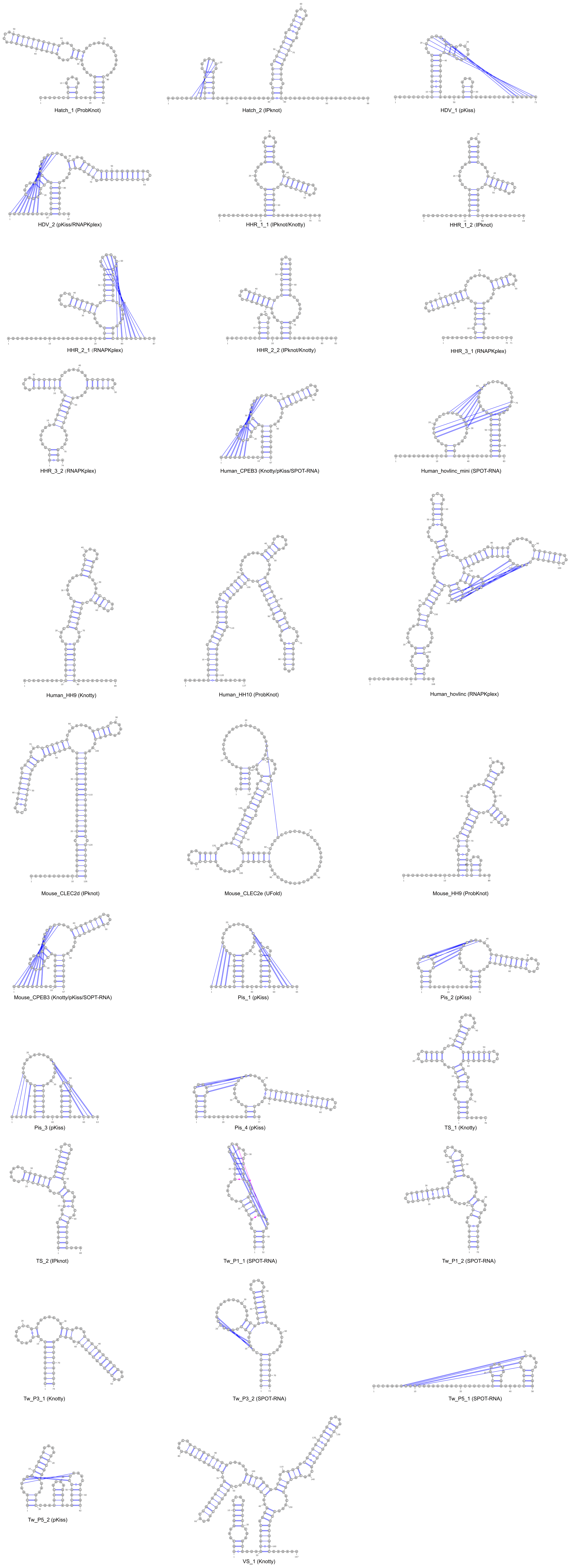
Hatch_1 (ProbKnot)
Hatch_2 (IPknot)
HDV_1 (pKiss)
HDV_2 (pKiss/RNAPKplex)
HHR_1_1 (IPknot/Knotty)
HHR_1_2 (IPknot)
HHR_2_1 (RNAPKplex)
HHR_2_2 (IPknot/Knotty)
HHR_3_1 (RNAPKplex)
HHR_3_2 (RNAPKplex)
Human_CPEB3 (Knotty/pKiss/SPOT-RNA)
Human_hovlinc_mini (SPOT-RNA)
Human_HH9 (Knotty)
Human_HH10 (ProbKnot)
Human_hovlinc (RNAPKplex)
Mouse_CLEC2d (IPknot)
Mouse_CLEC2e (UFold)
Mouse_HH9 (ProbKnot)
Mouse_CPEB3 (Knotty/pKiss/SOPT-RNA)
Pis_1 (pKiss)
Pis_2 (pKiss)
Pis_3 (pKiss)
Pis_4 (pKiss)
TS_1 (Knotty)
TS_2 (IPknot)
Tw_P1_1 (SPOT-RNA)
Tw_P1_2 (SPOT-RNA)
Tw_P3_1 (Knotty)
Tw_P3_2 (SPOT-RNA)
Tw_P5_1 (SPOT-RNA)
Tw_P5_2 (pKiss)
VS_1 (Knotty)
