# Supplementary figures and images for: Evaluating Performance of Different RNA Secondary Structure Prediction Programs Using Self-cleaving Ribozymes
Source: Genomics Proteomics Bioinformatics. 2024 Jun 8;22(3):qzae043. doi: 10.1093/gpbjnl/qzae043 (PMC12016570; doi:10.1093/gpbjnl/qzae043)

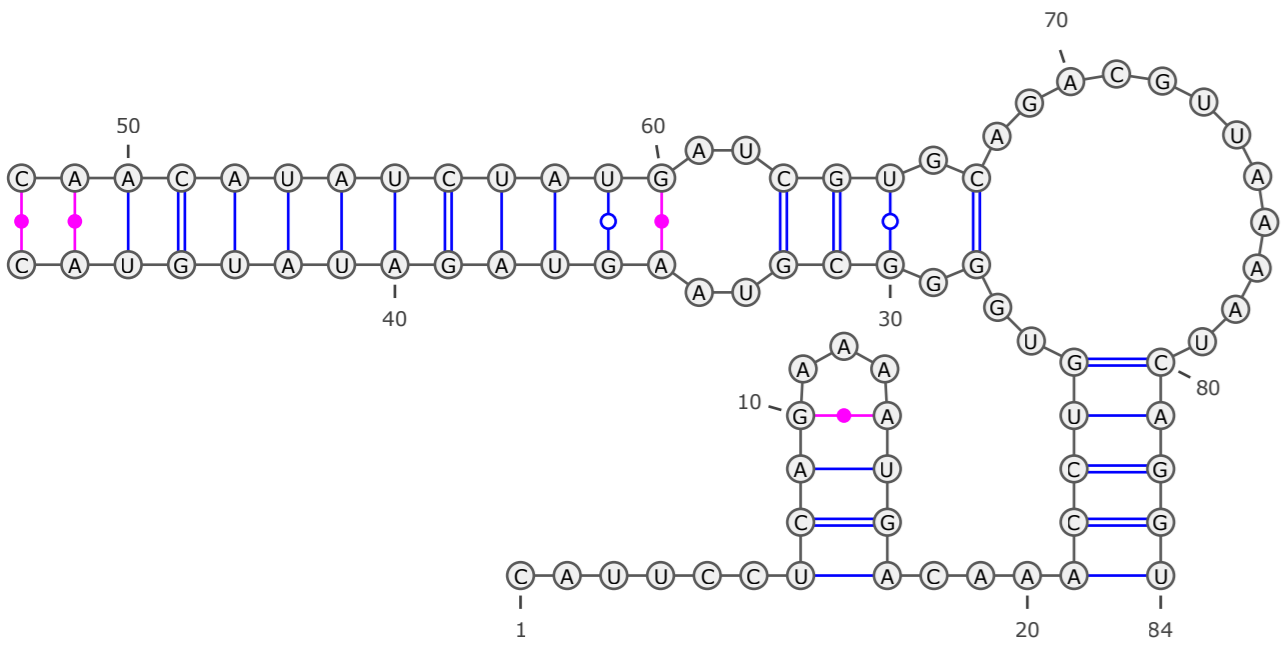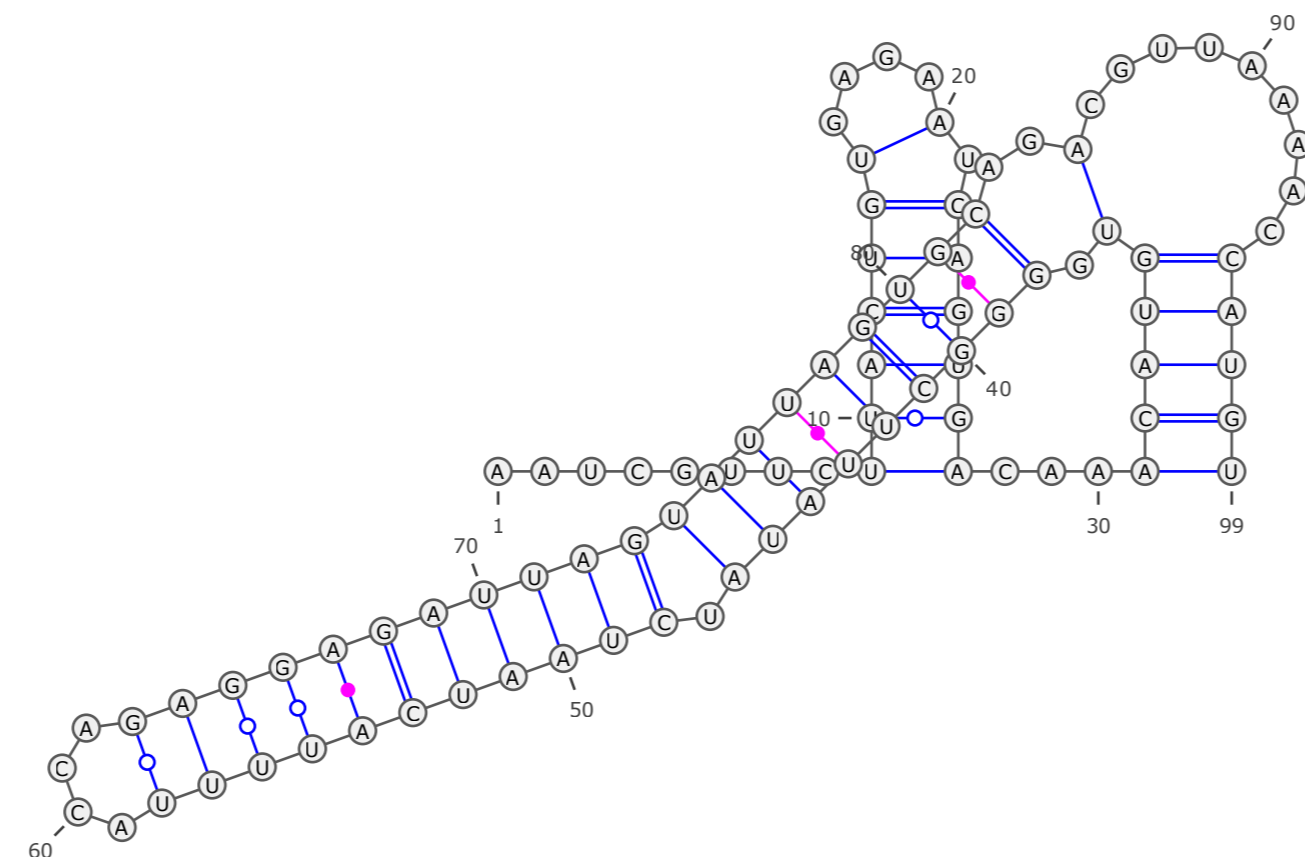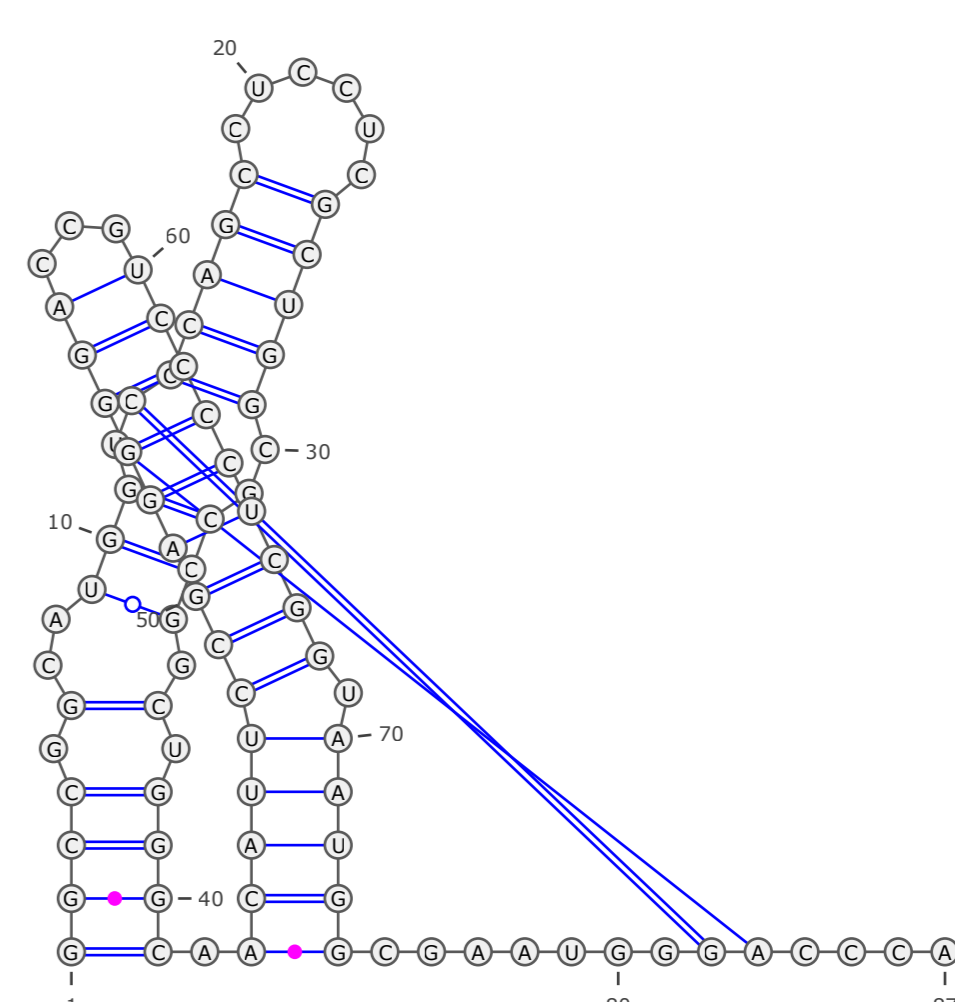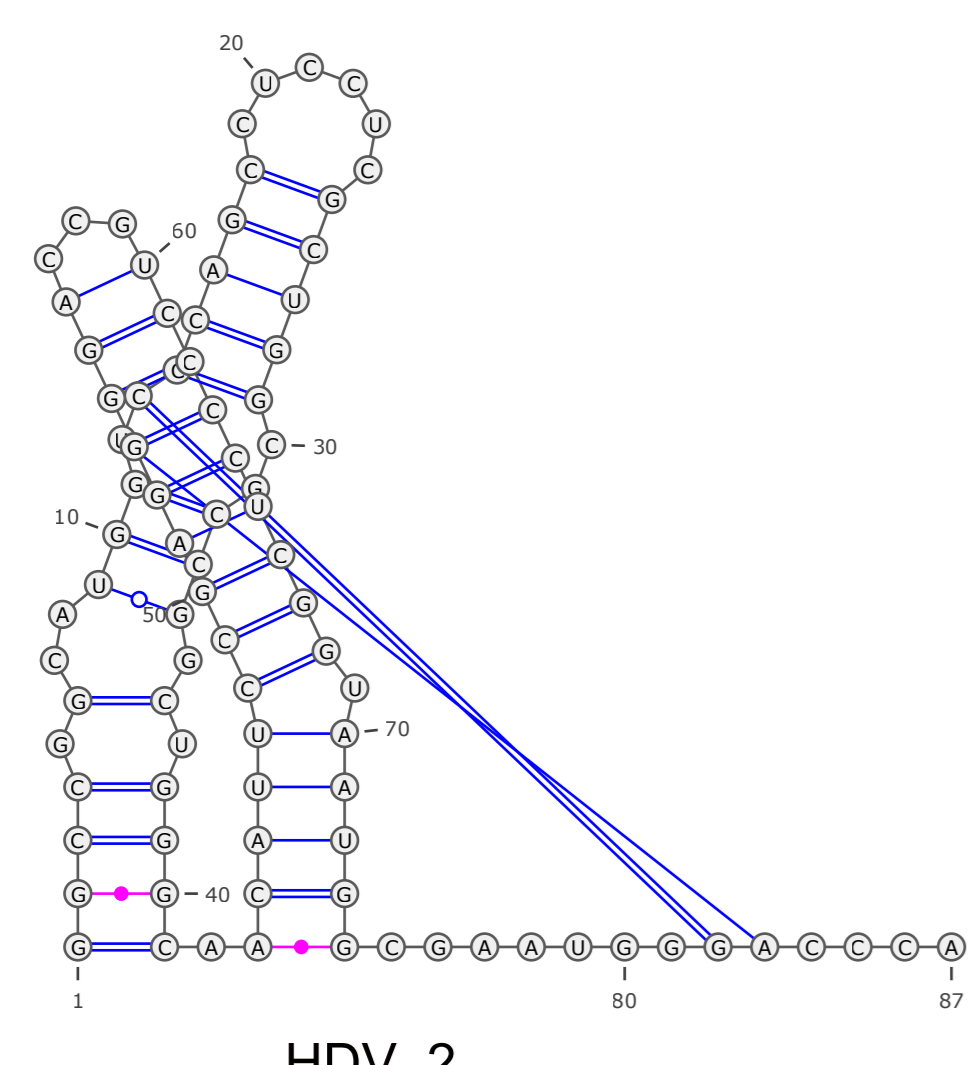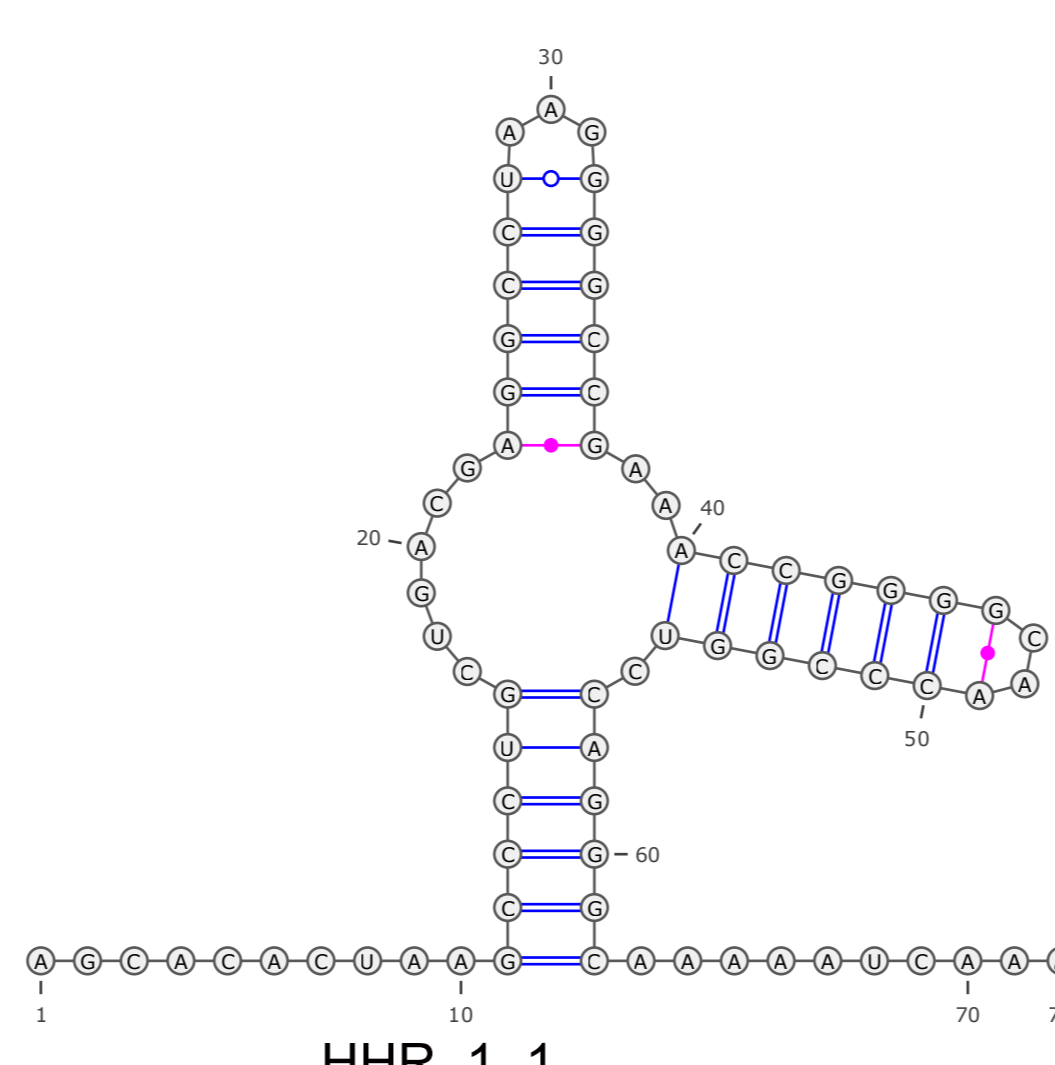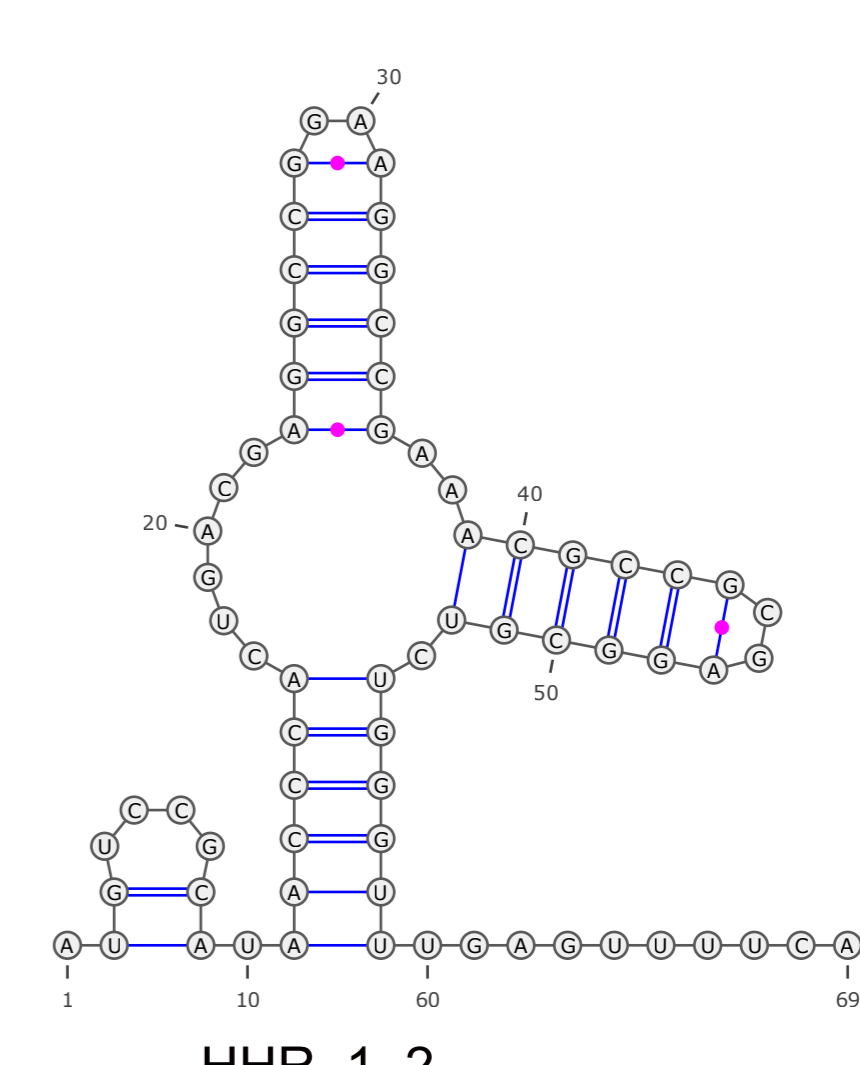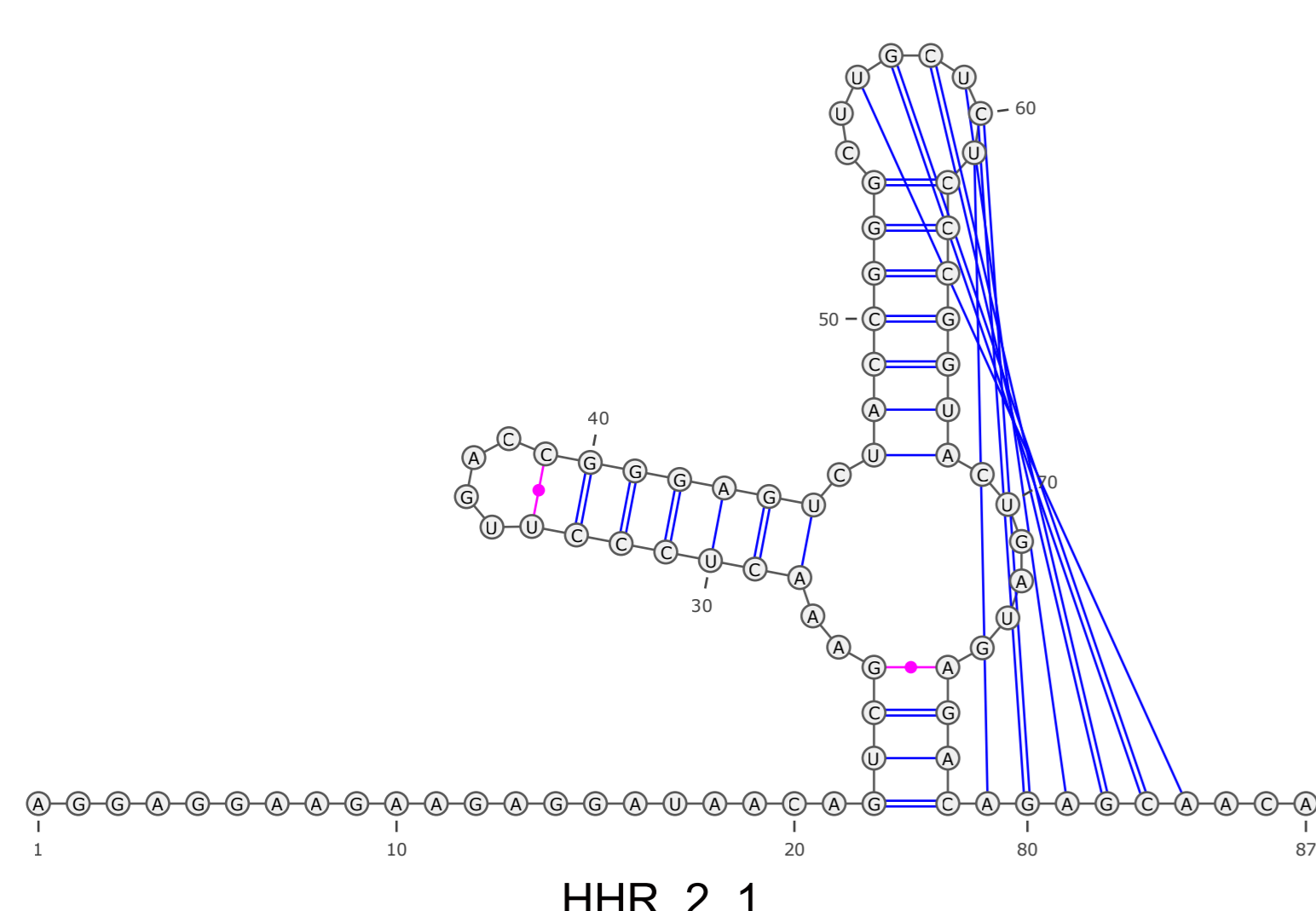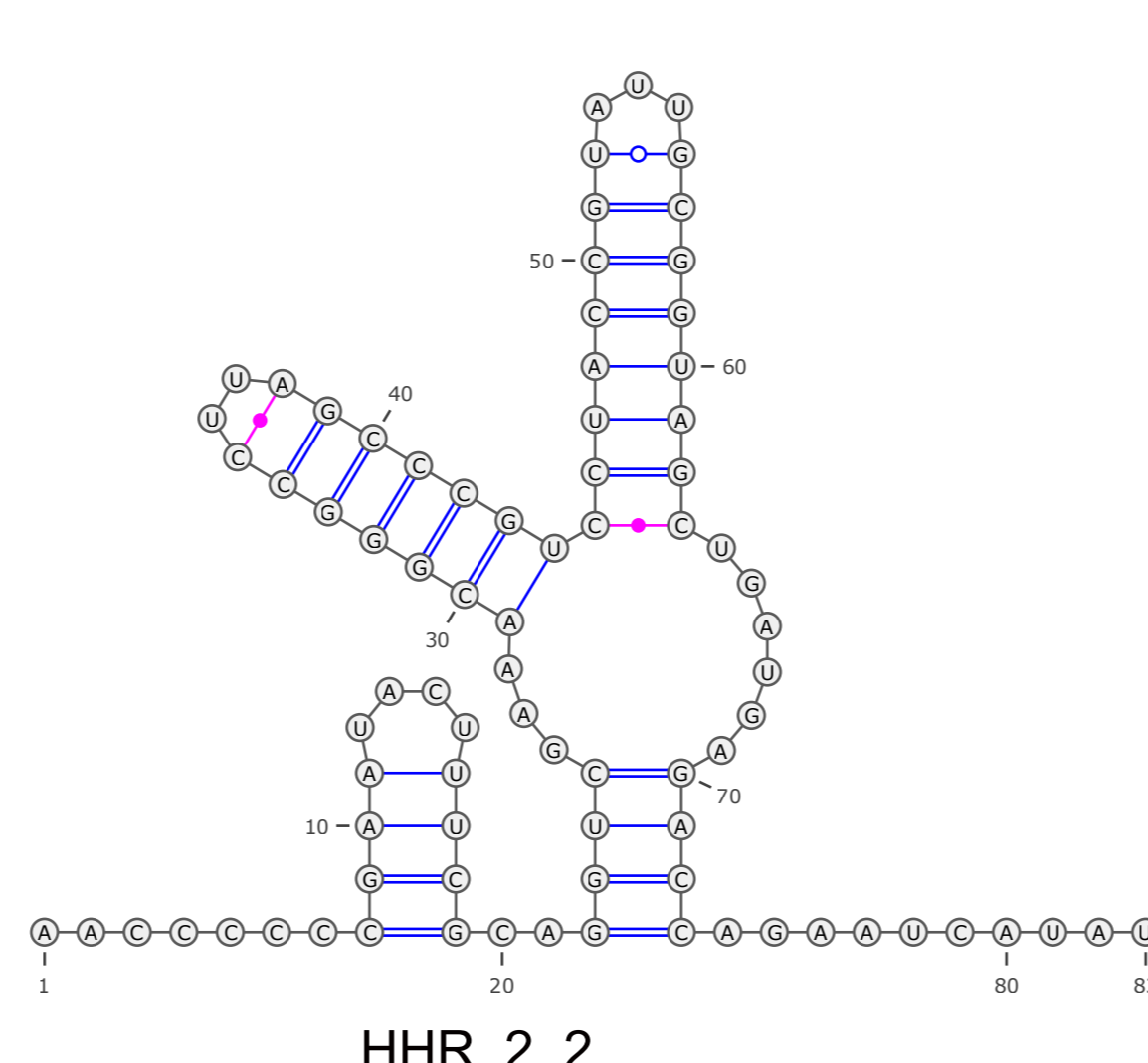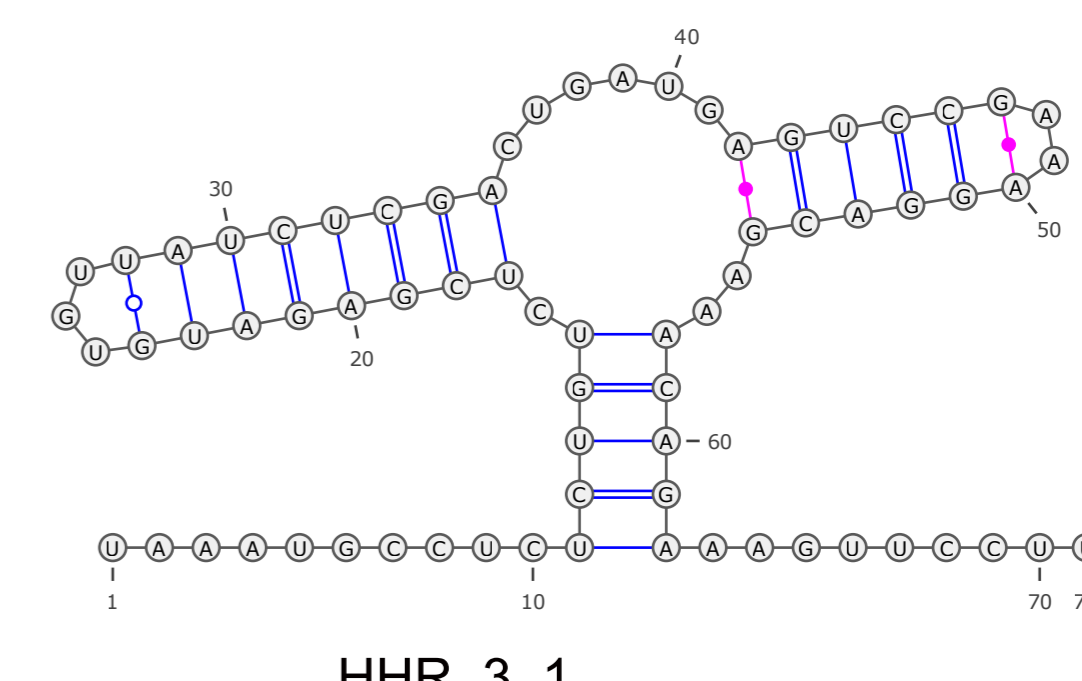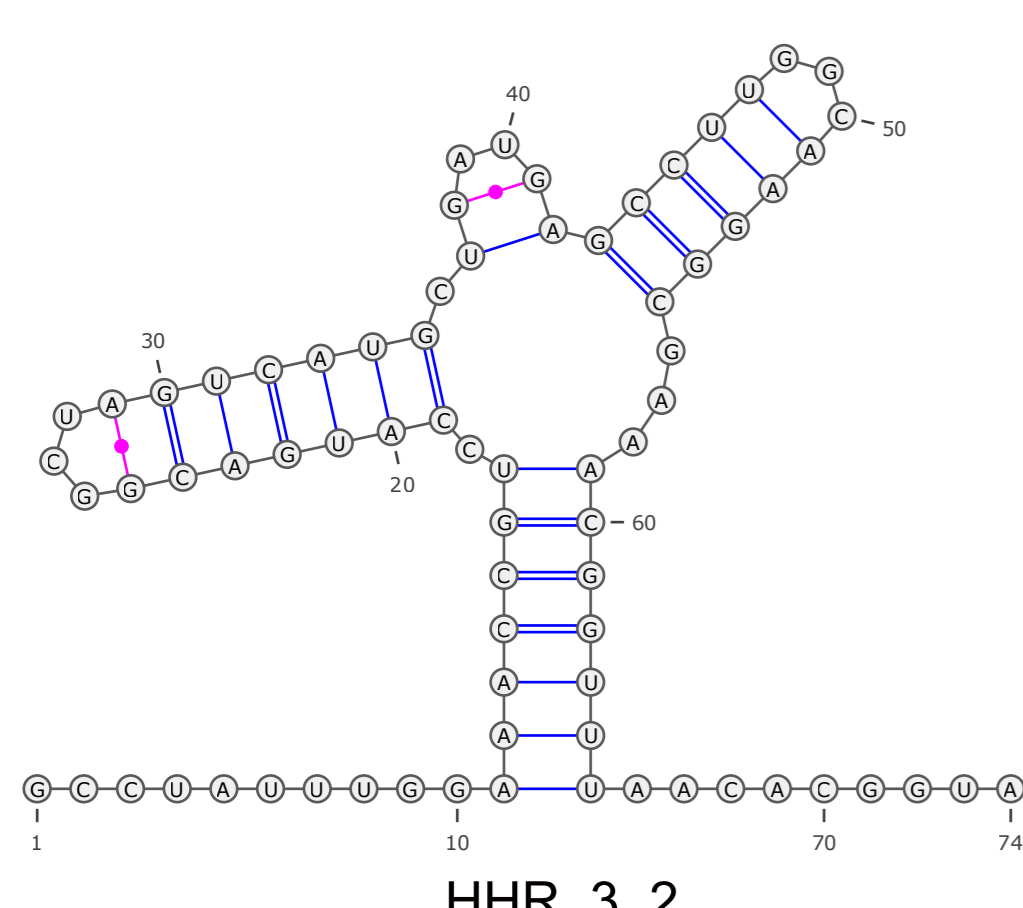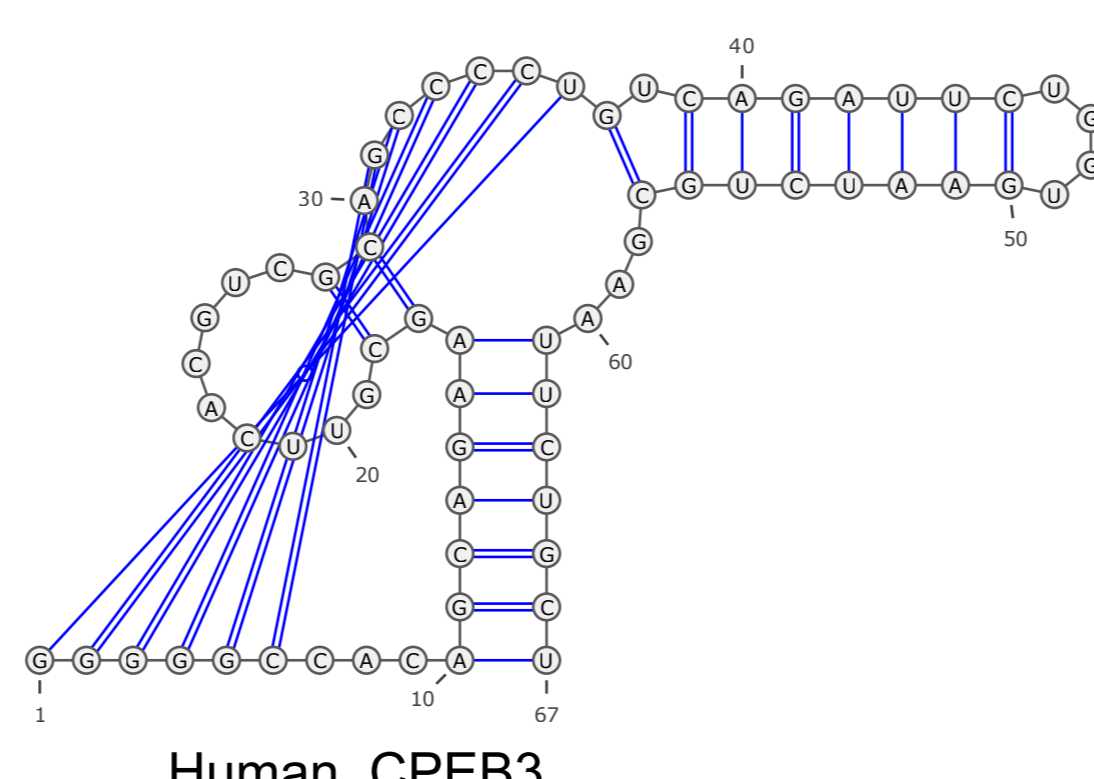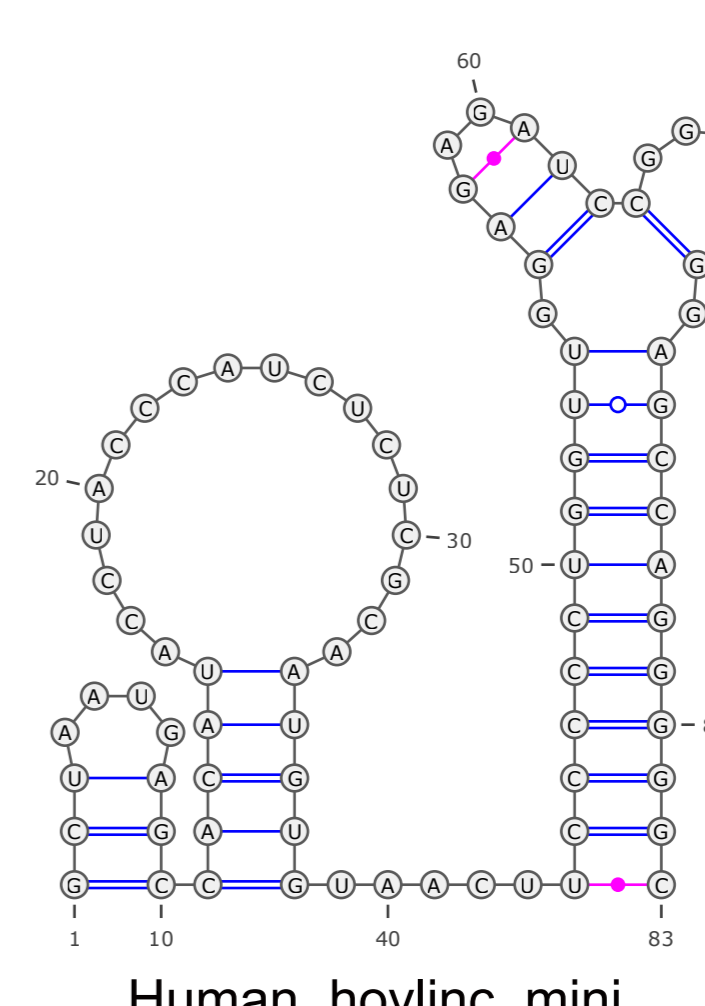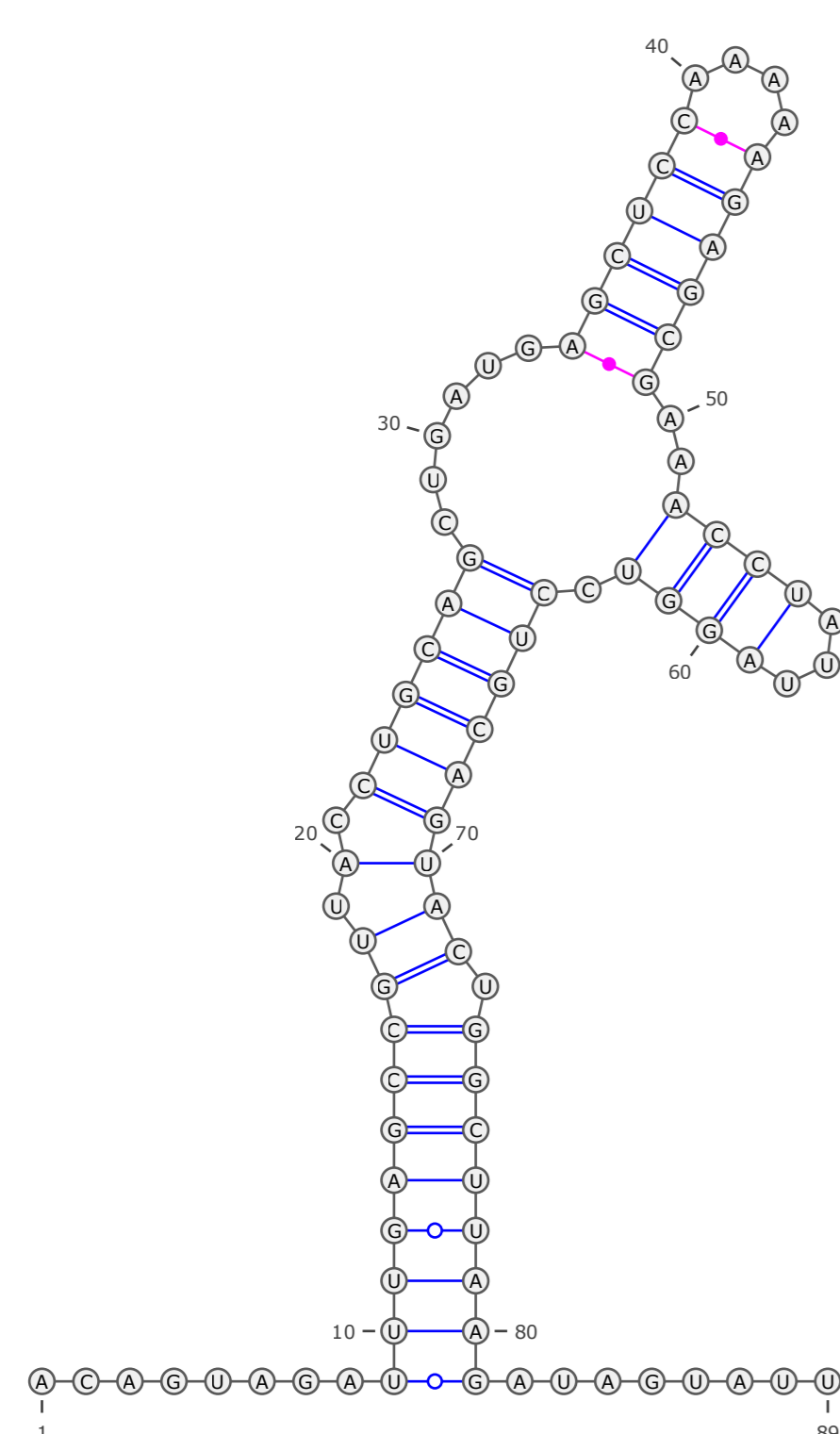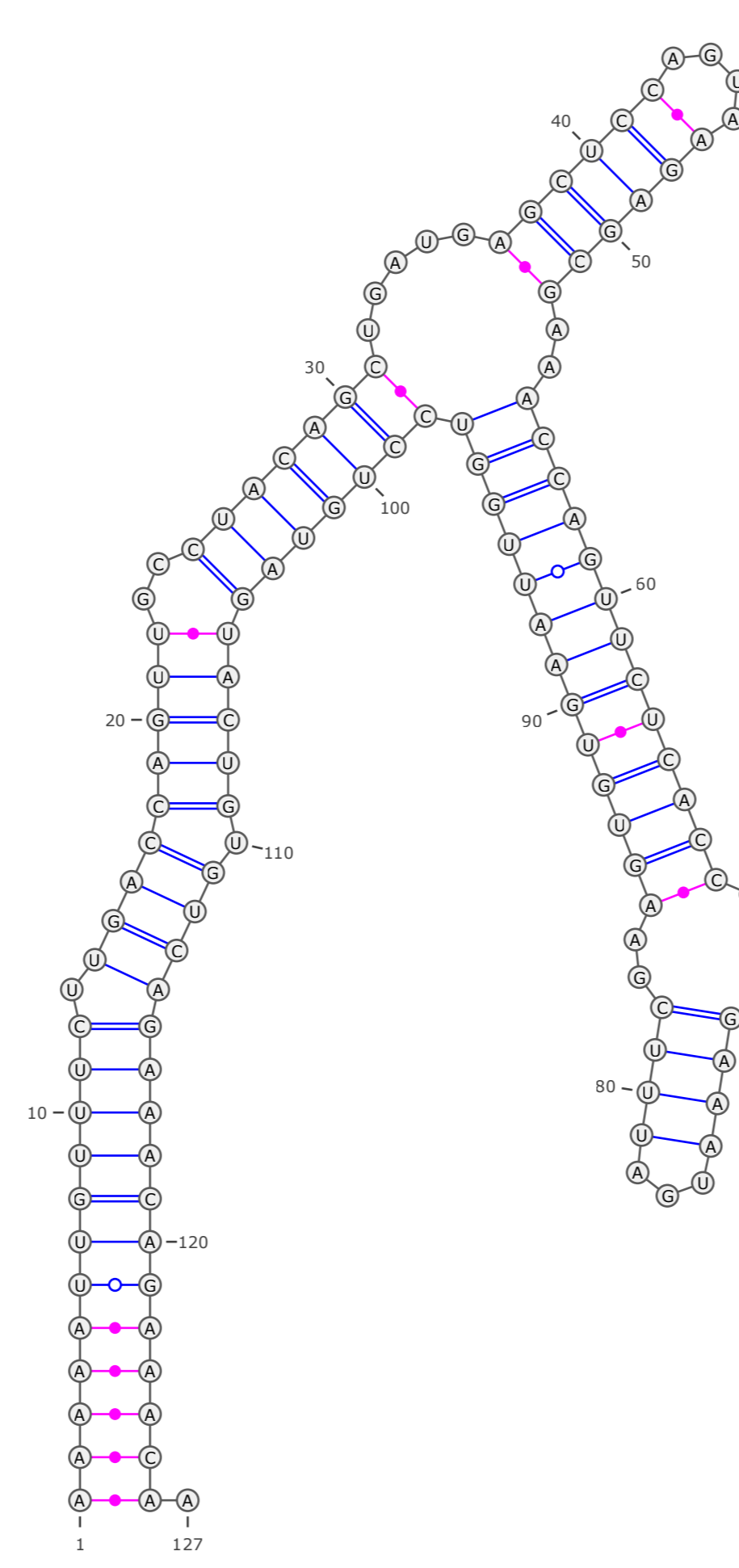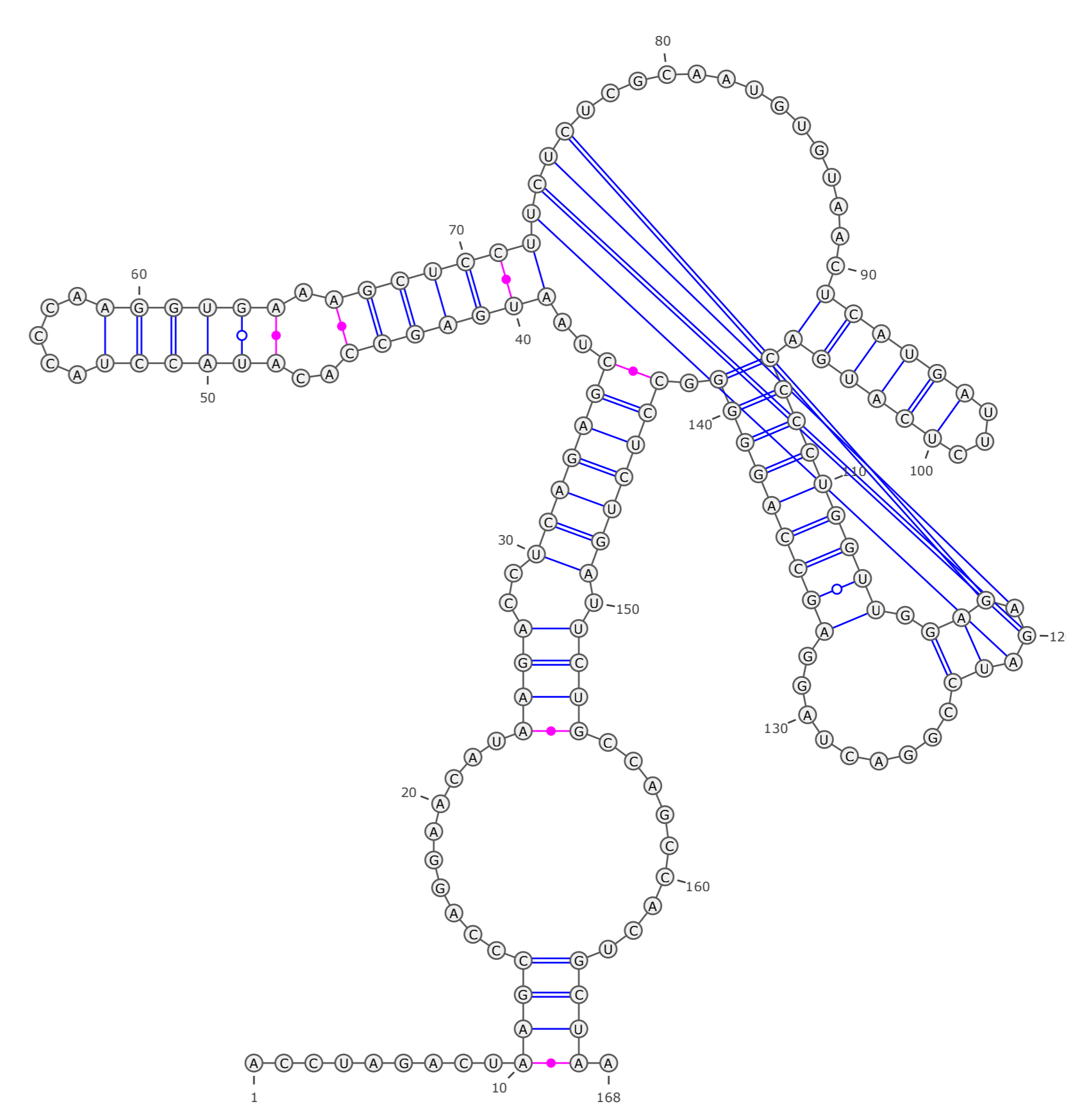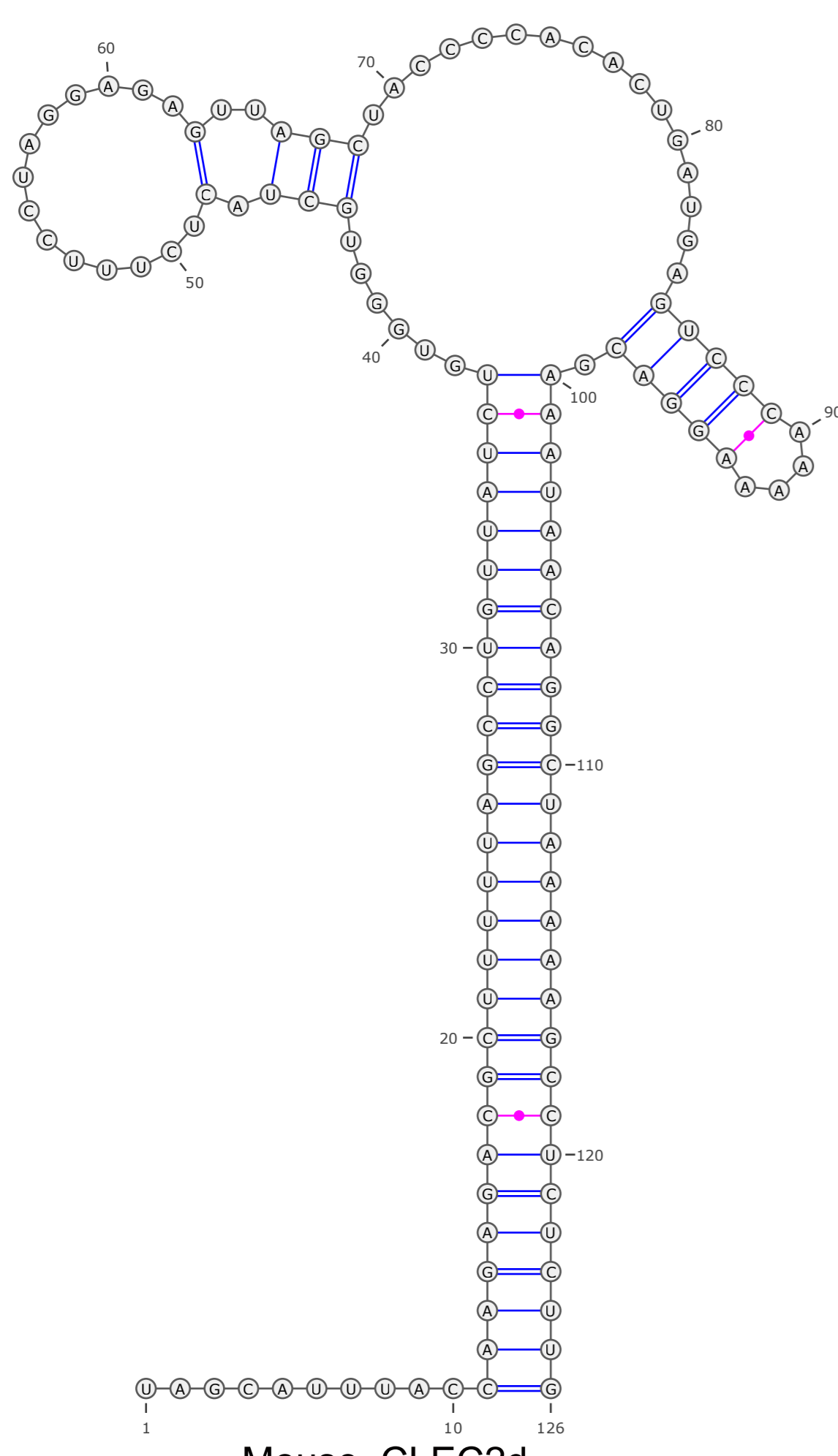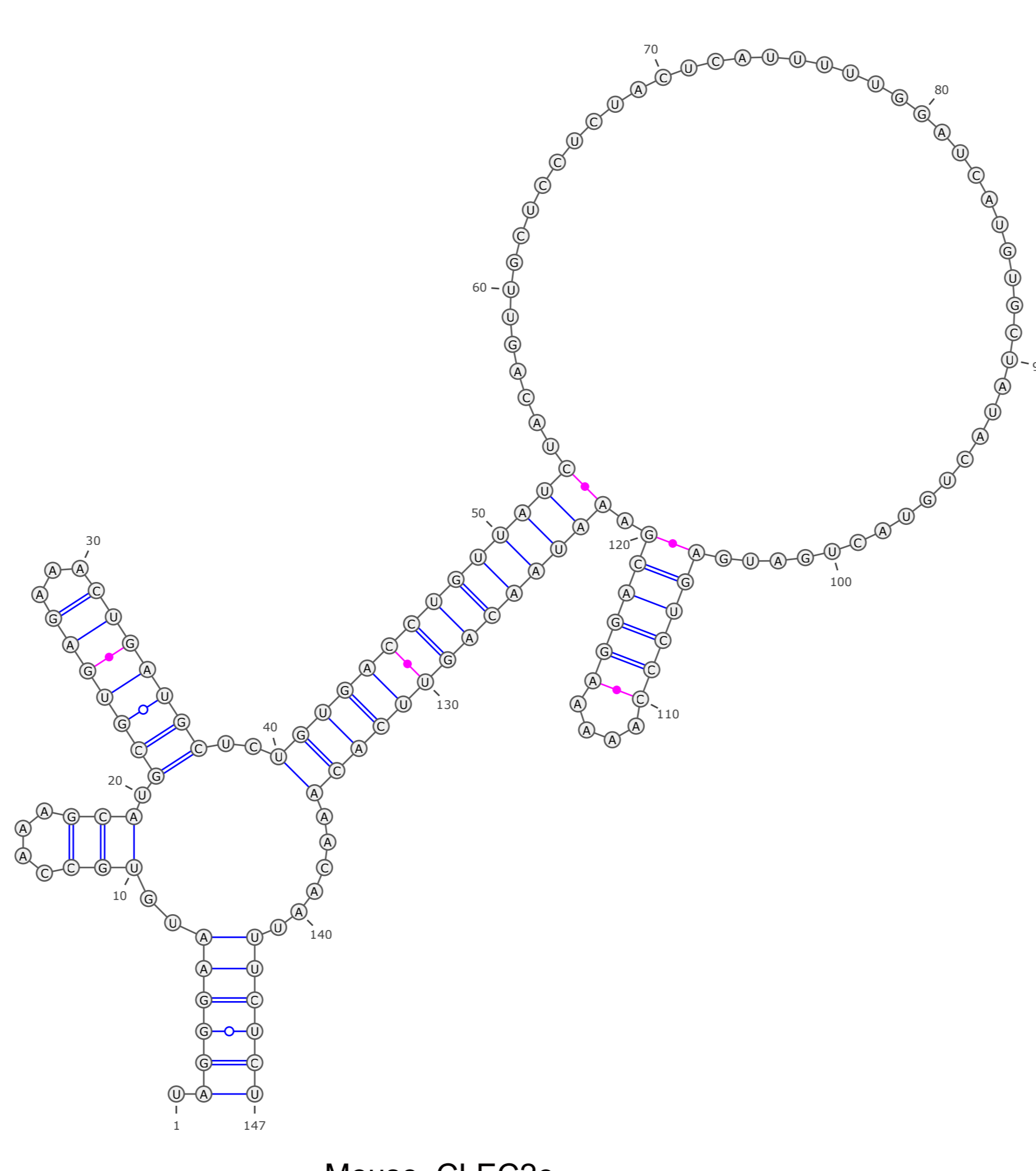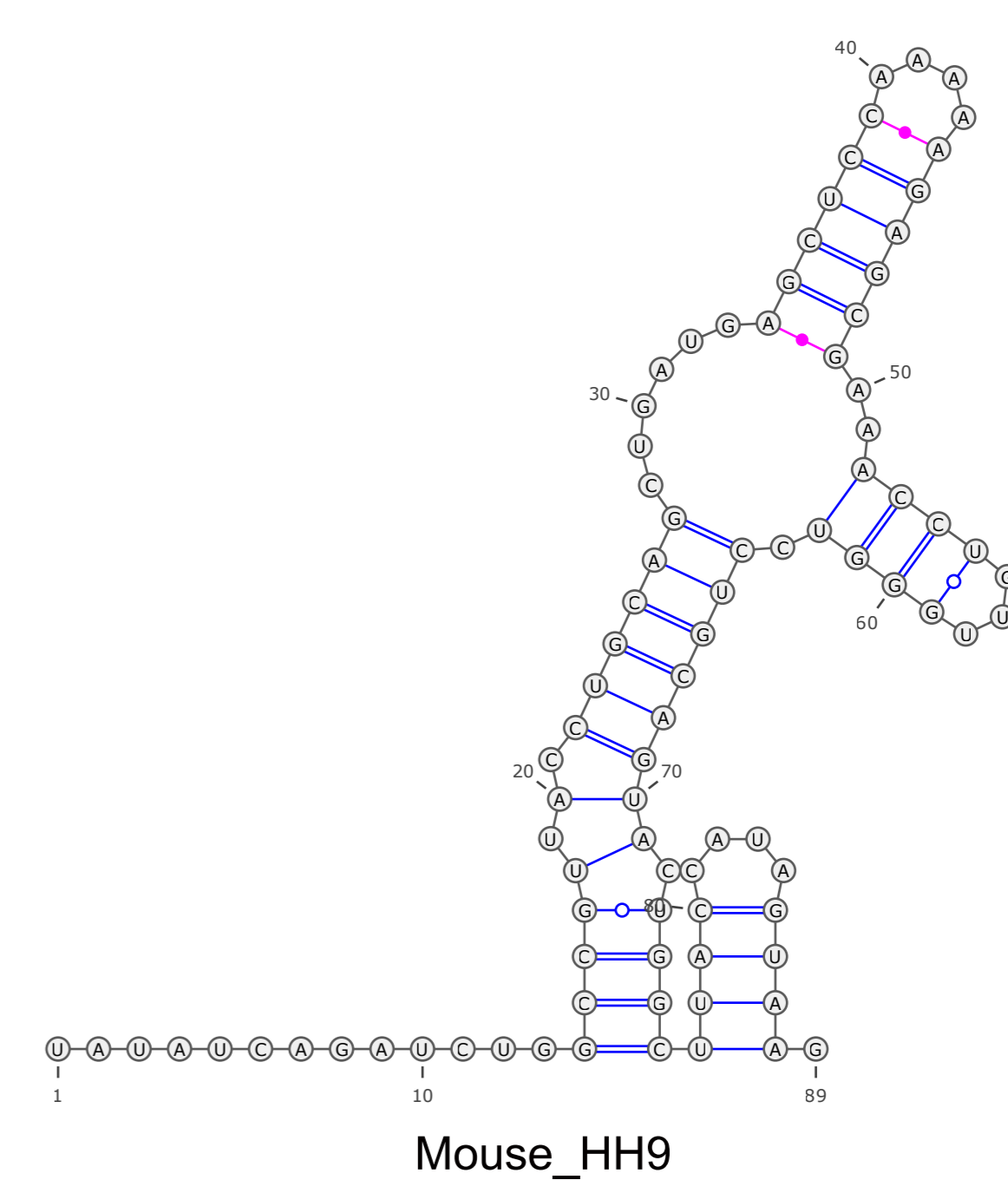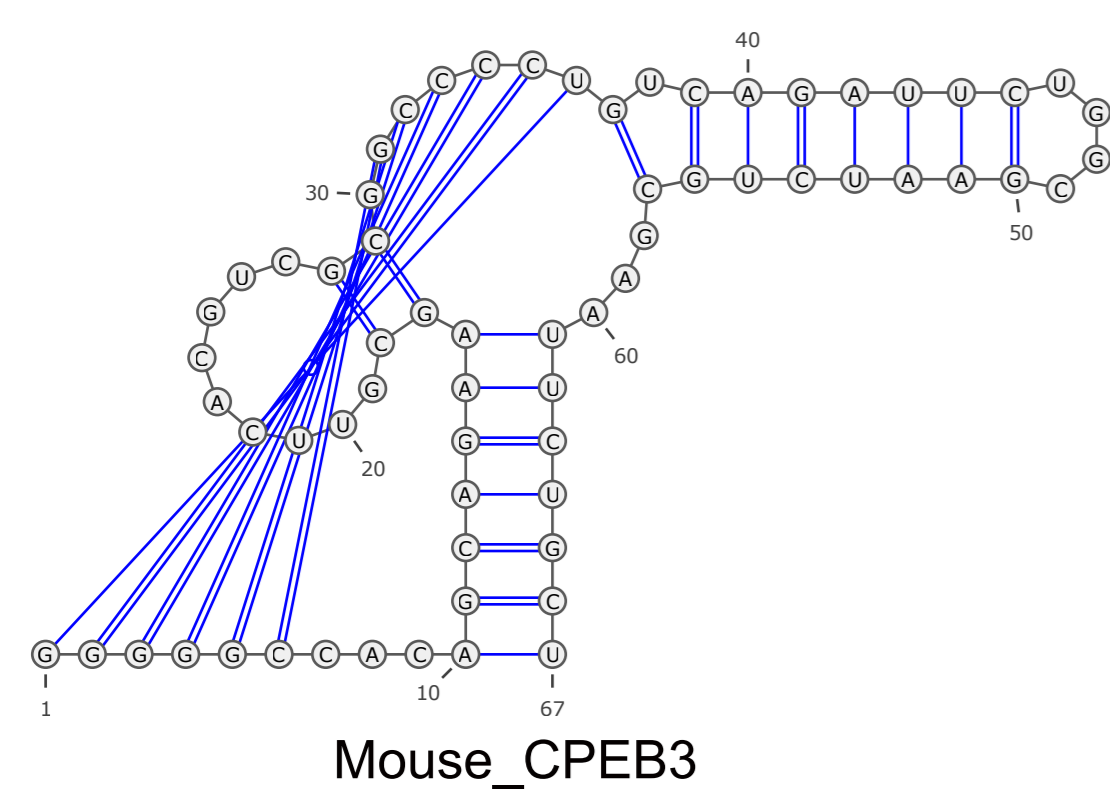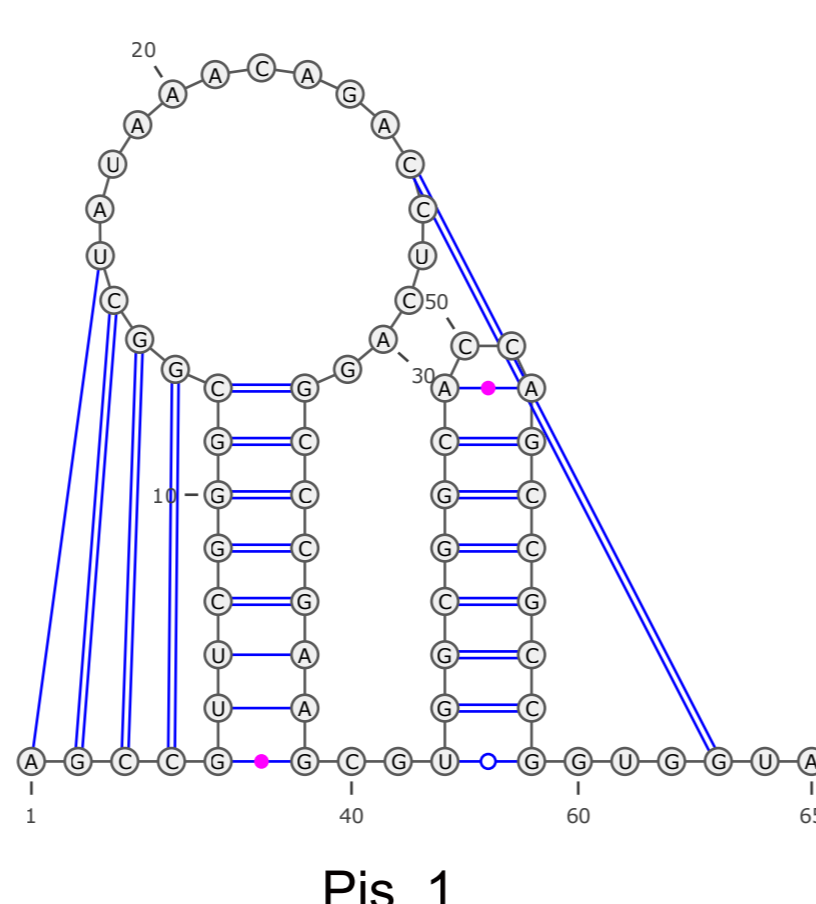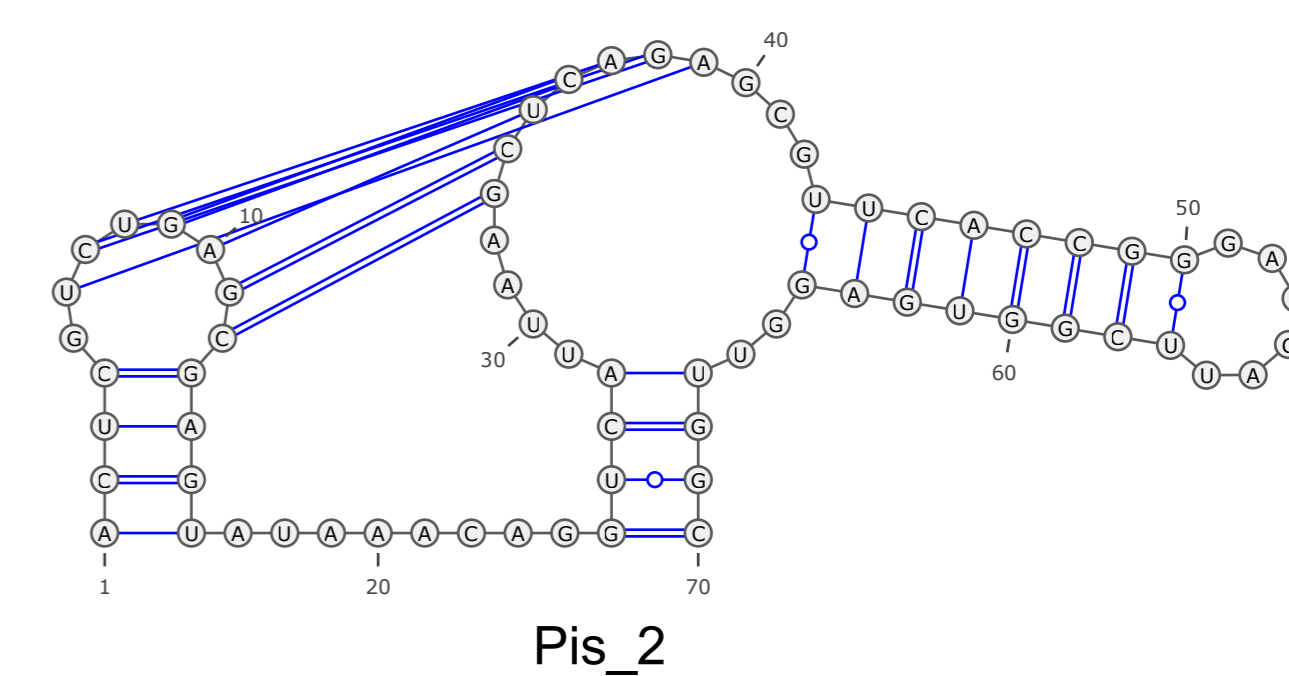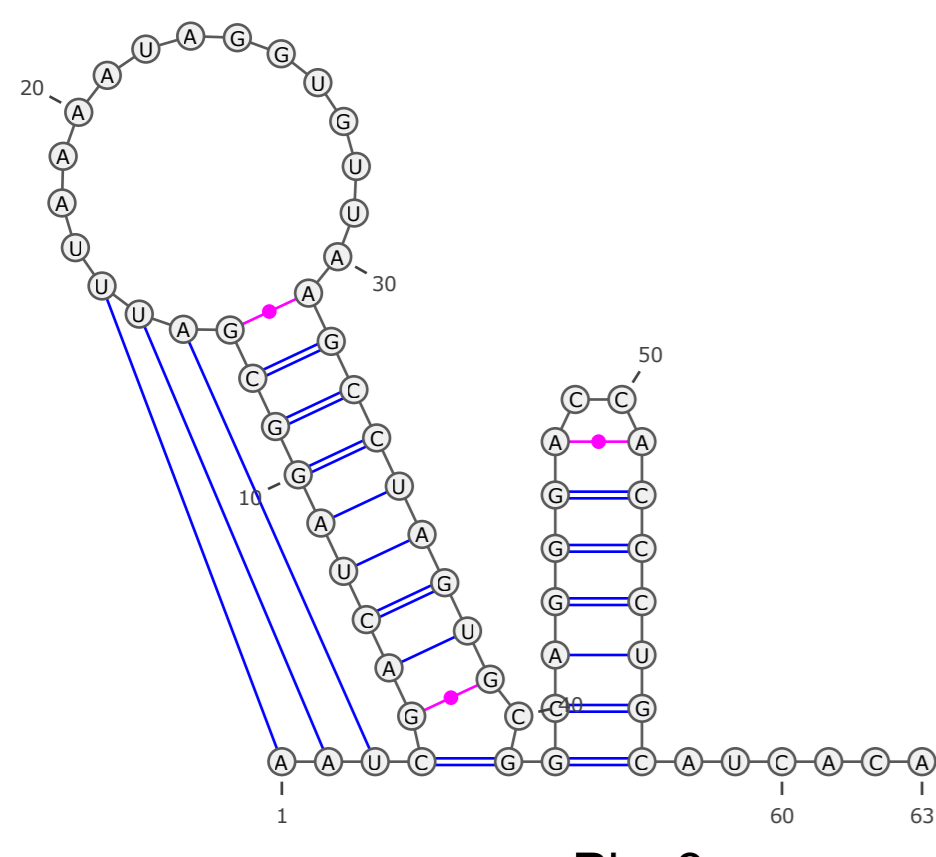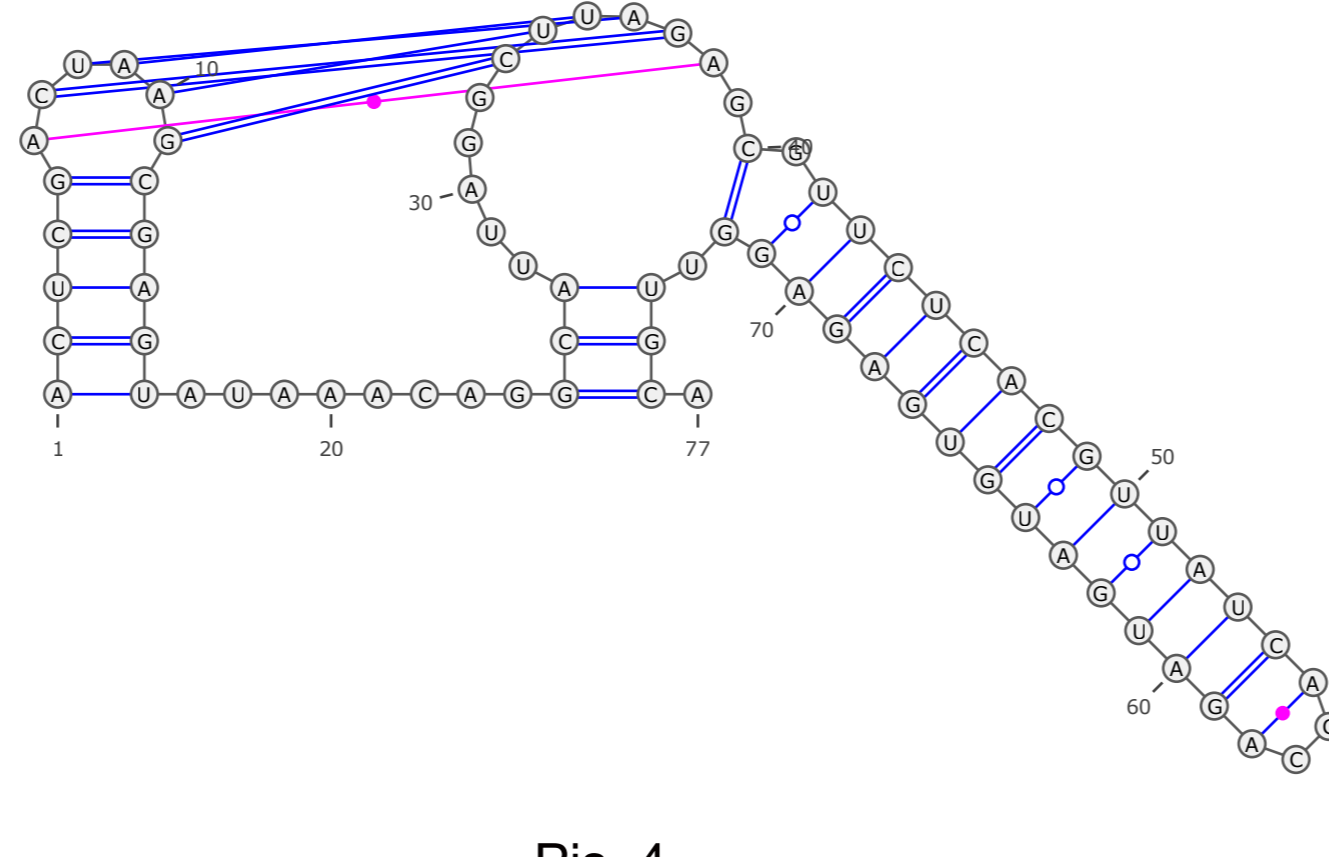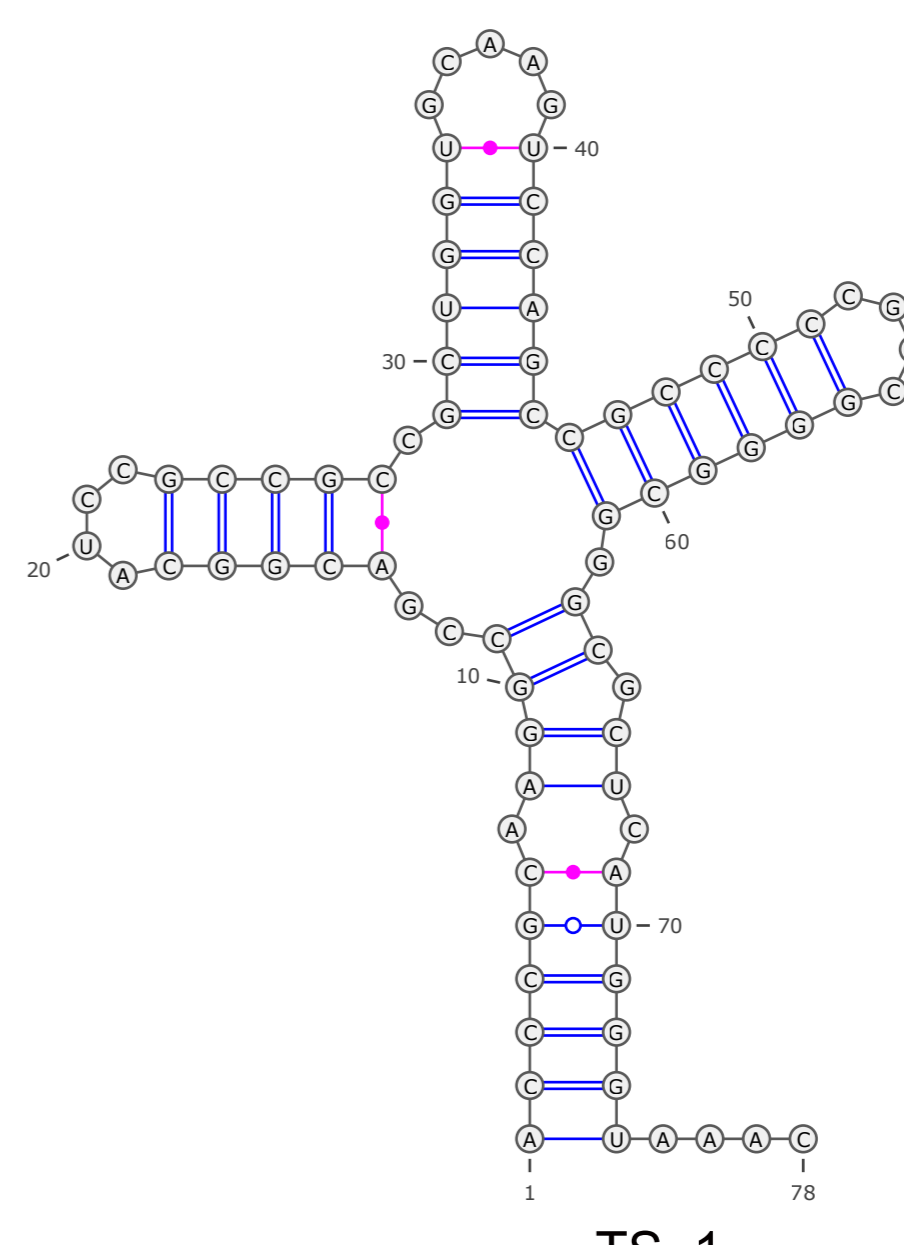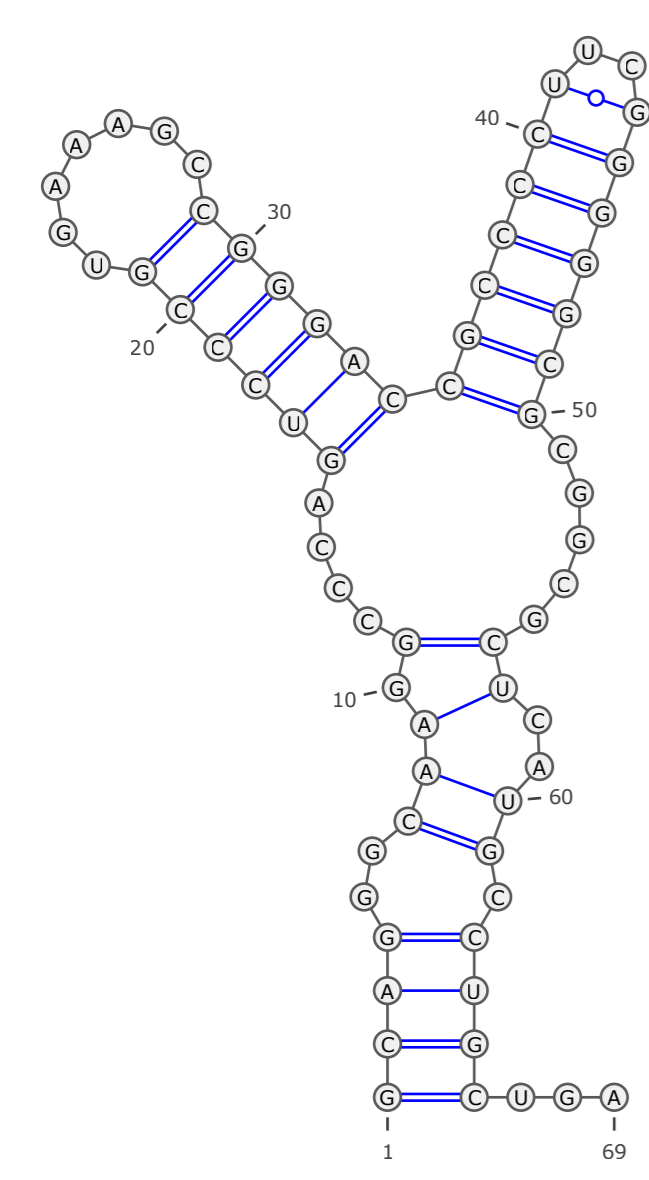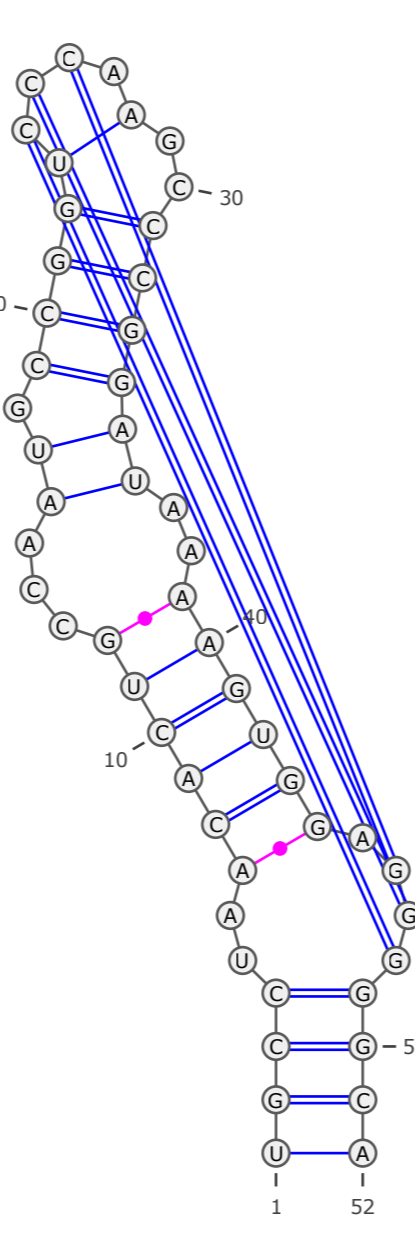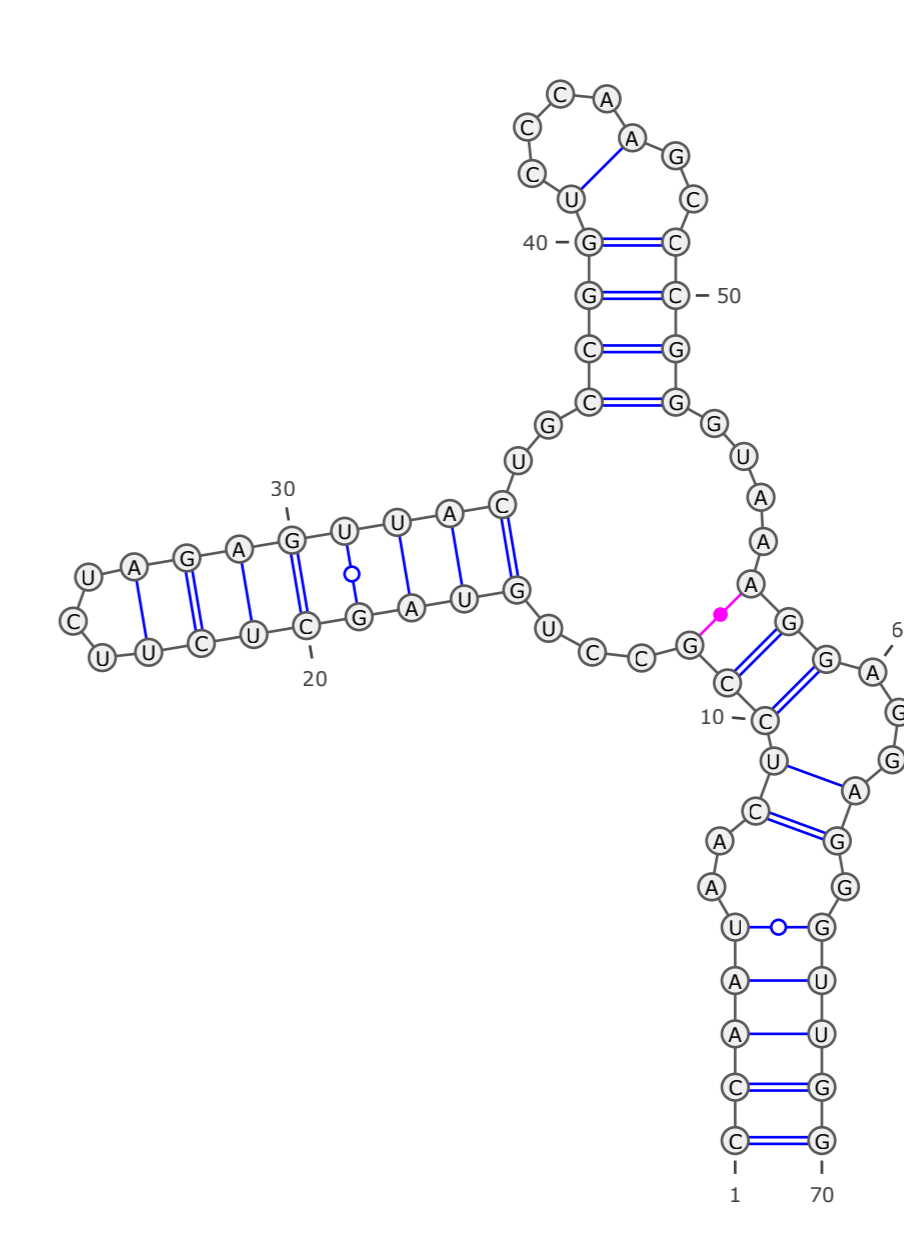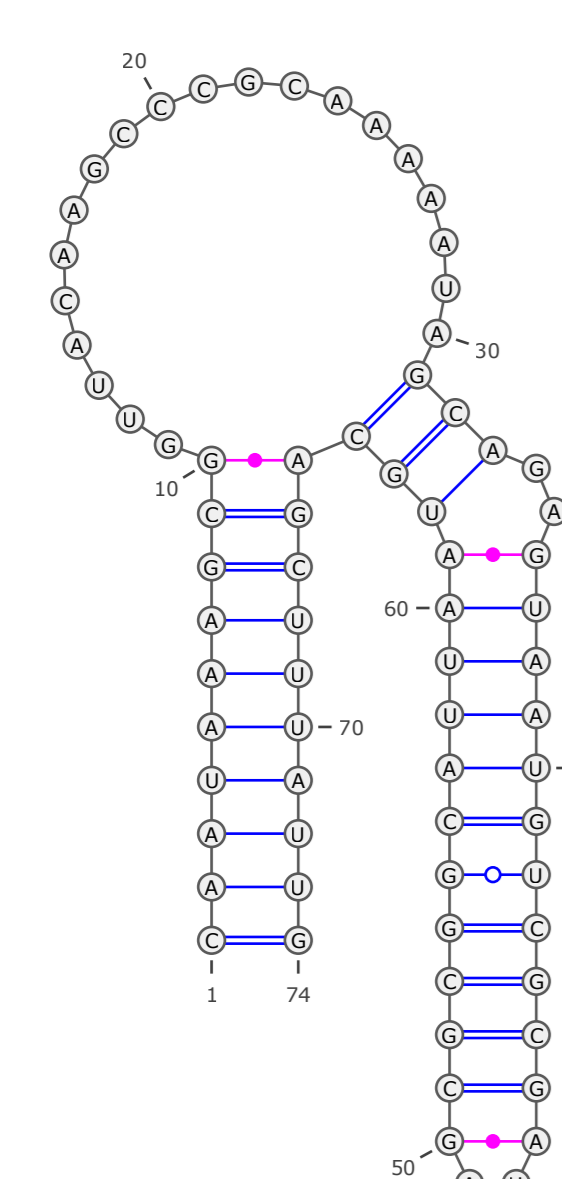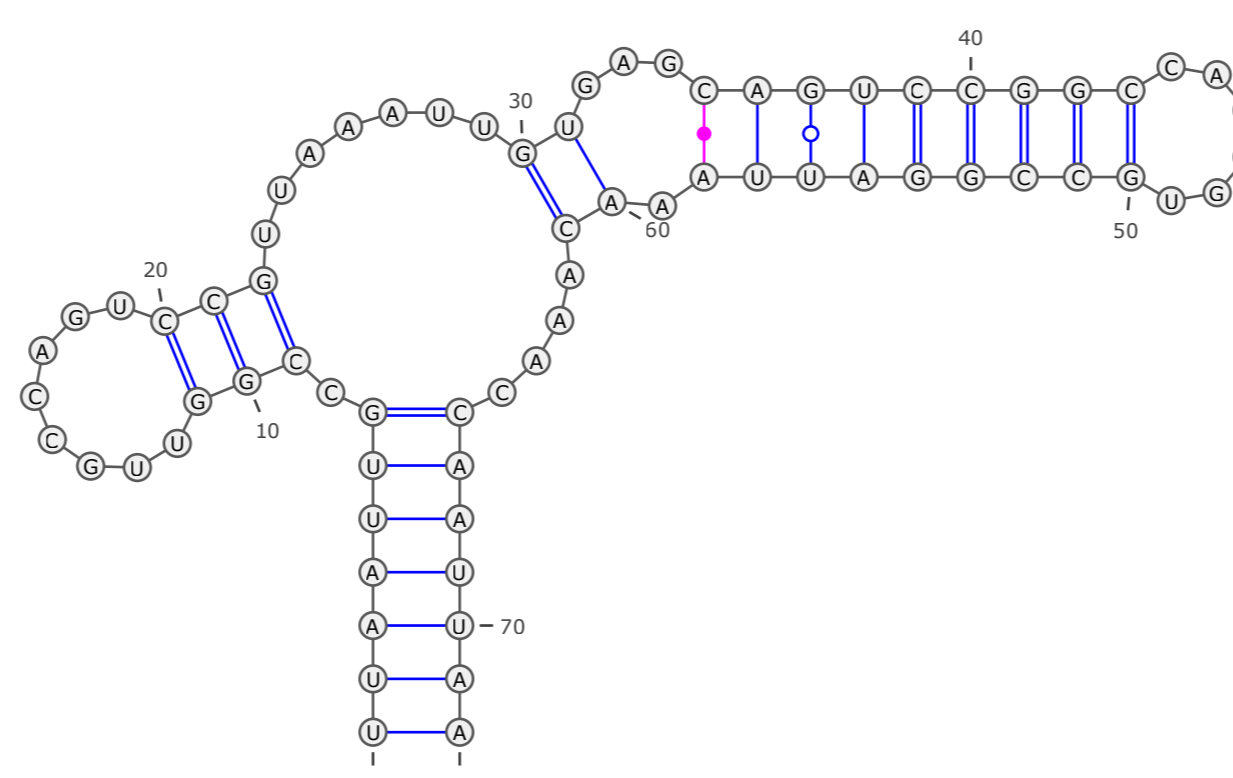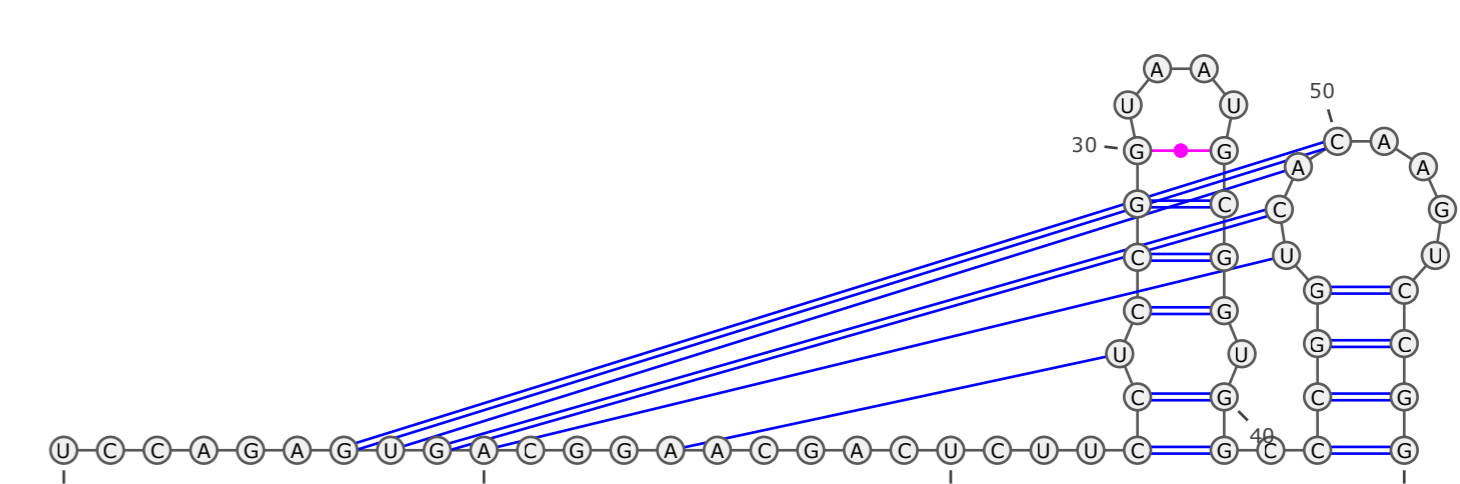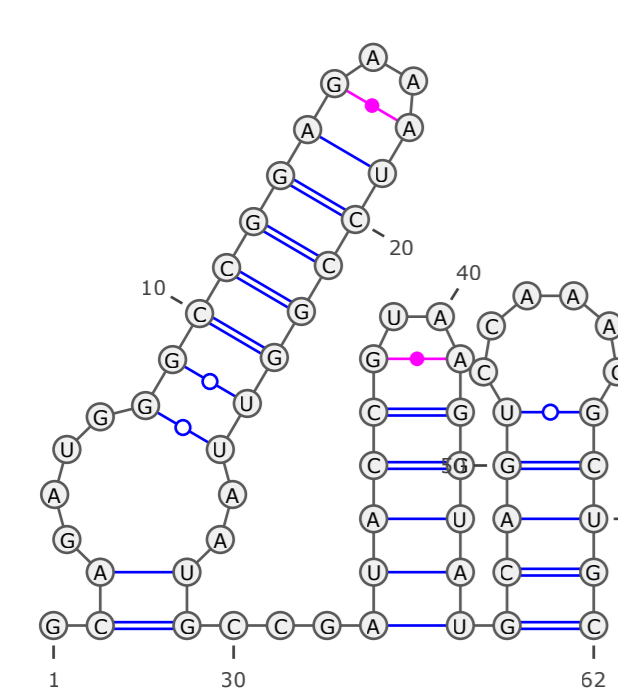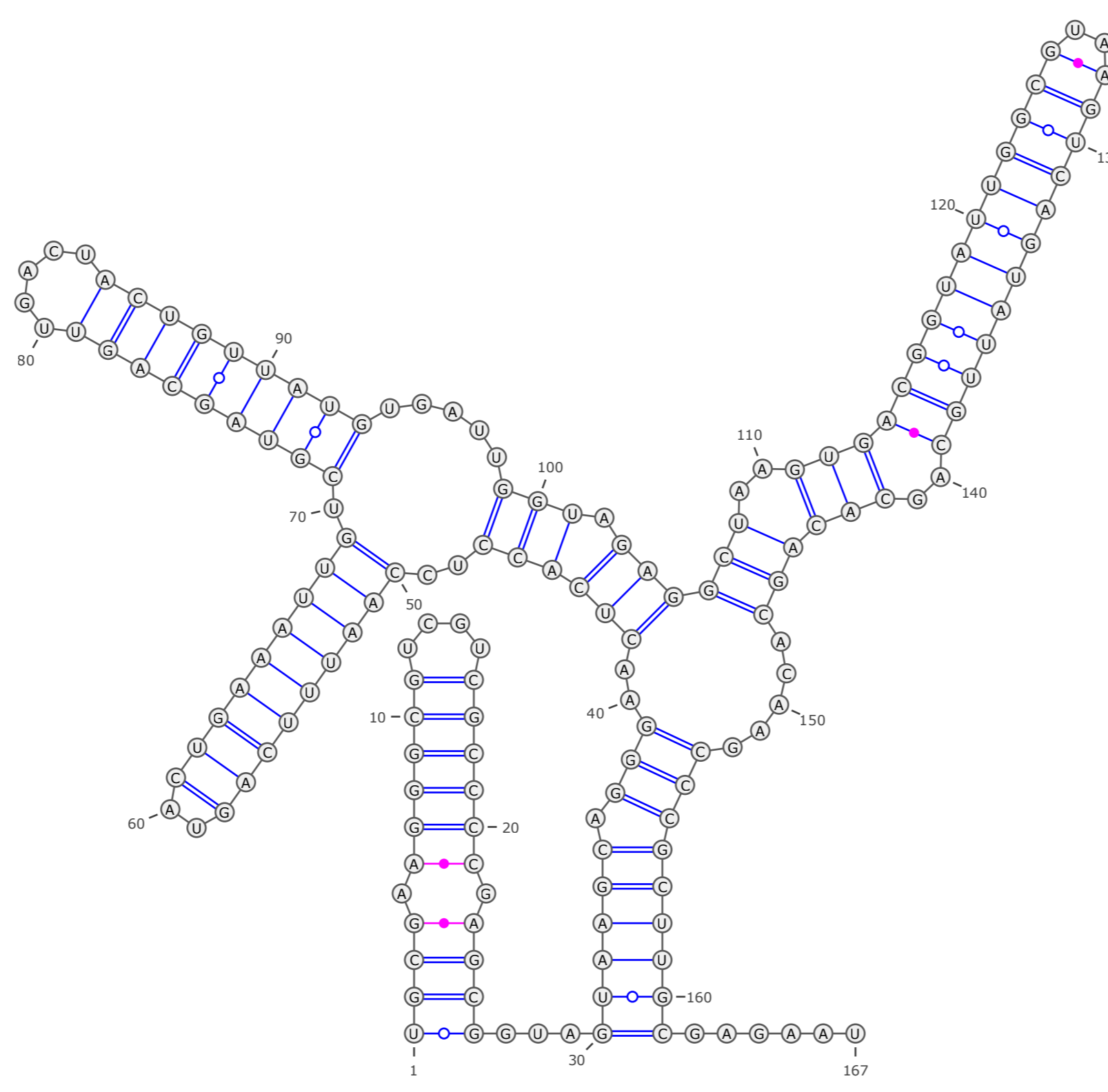

Supplement: qzae043_Supplementary_Data [file qzae043_supplementary_data.zip › Figure_S5.pdf]

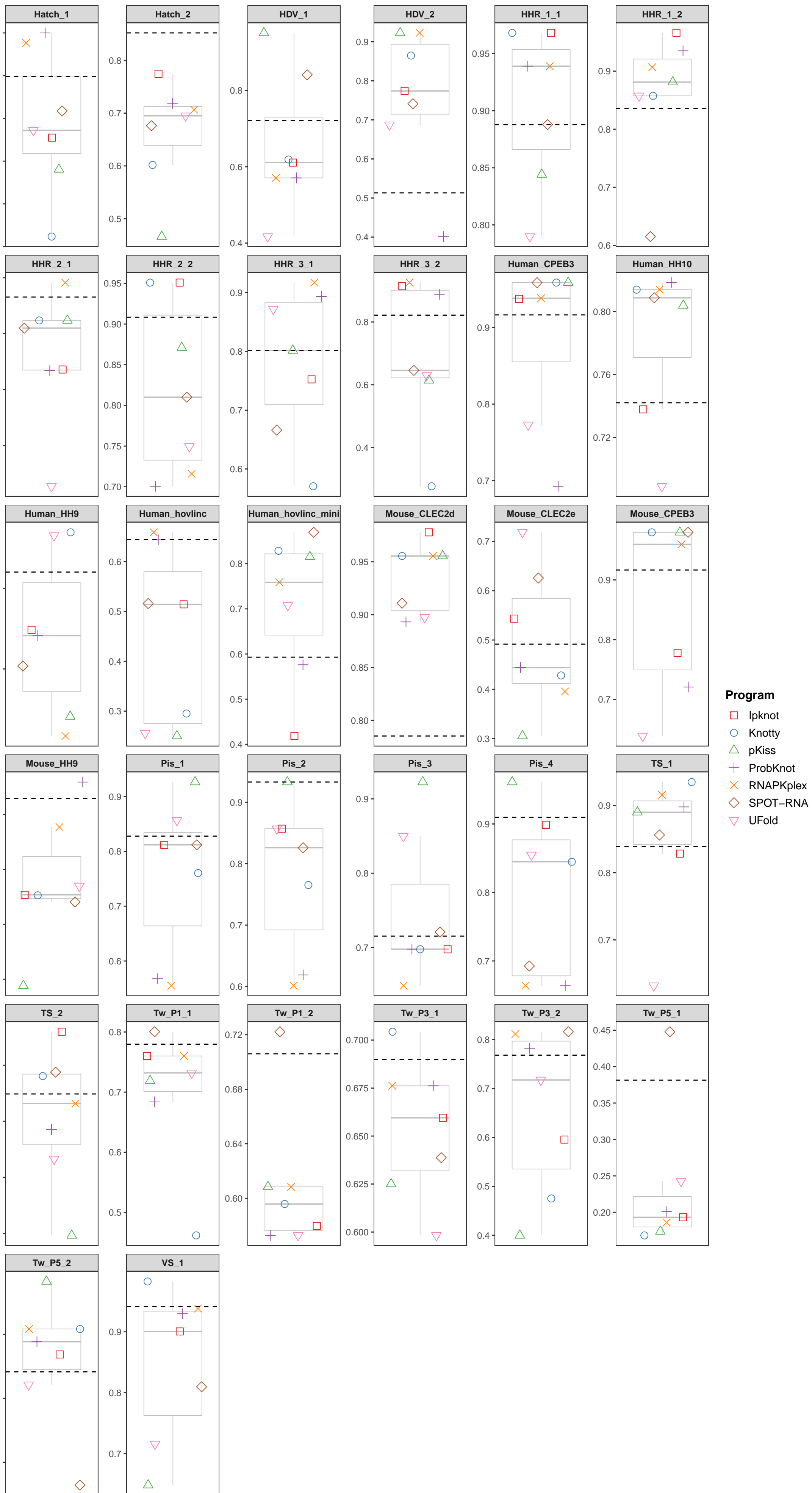

Supplement: qzae043_Supplementary_Data [file qzae043_supplementary_data.zip › Figure_S6.pdf]

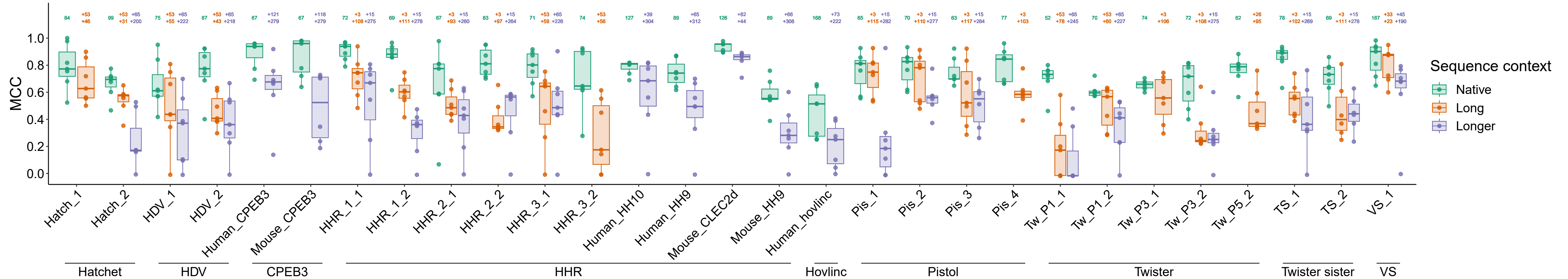

Supplement: qzae043_Supplementary_Data [file qzae043_supplementary_data.zip › Figure_S8.pdf]

A

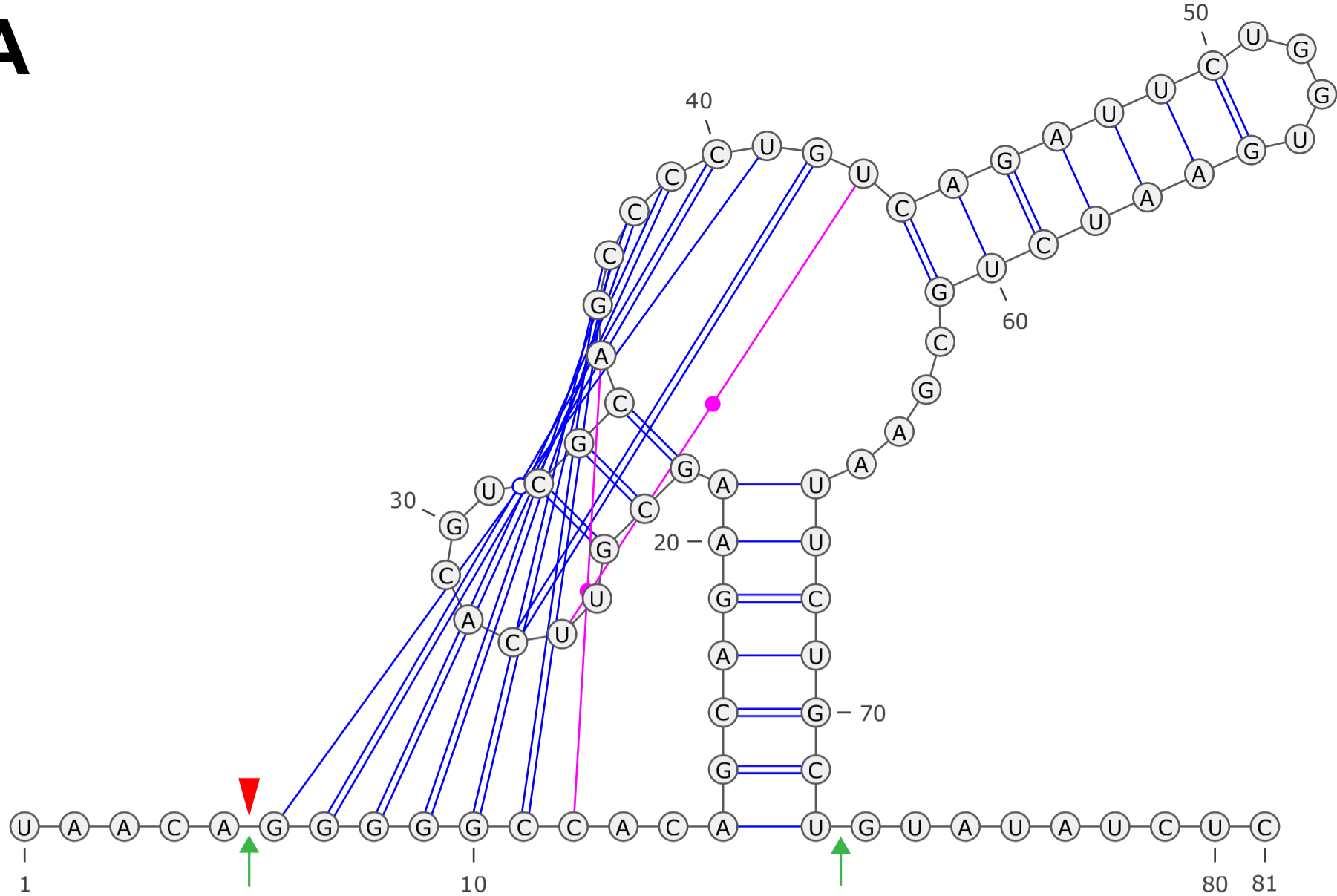

B

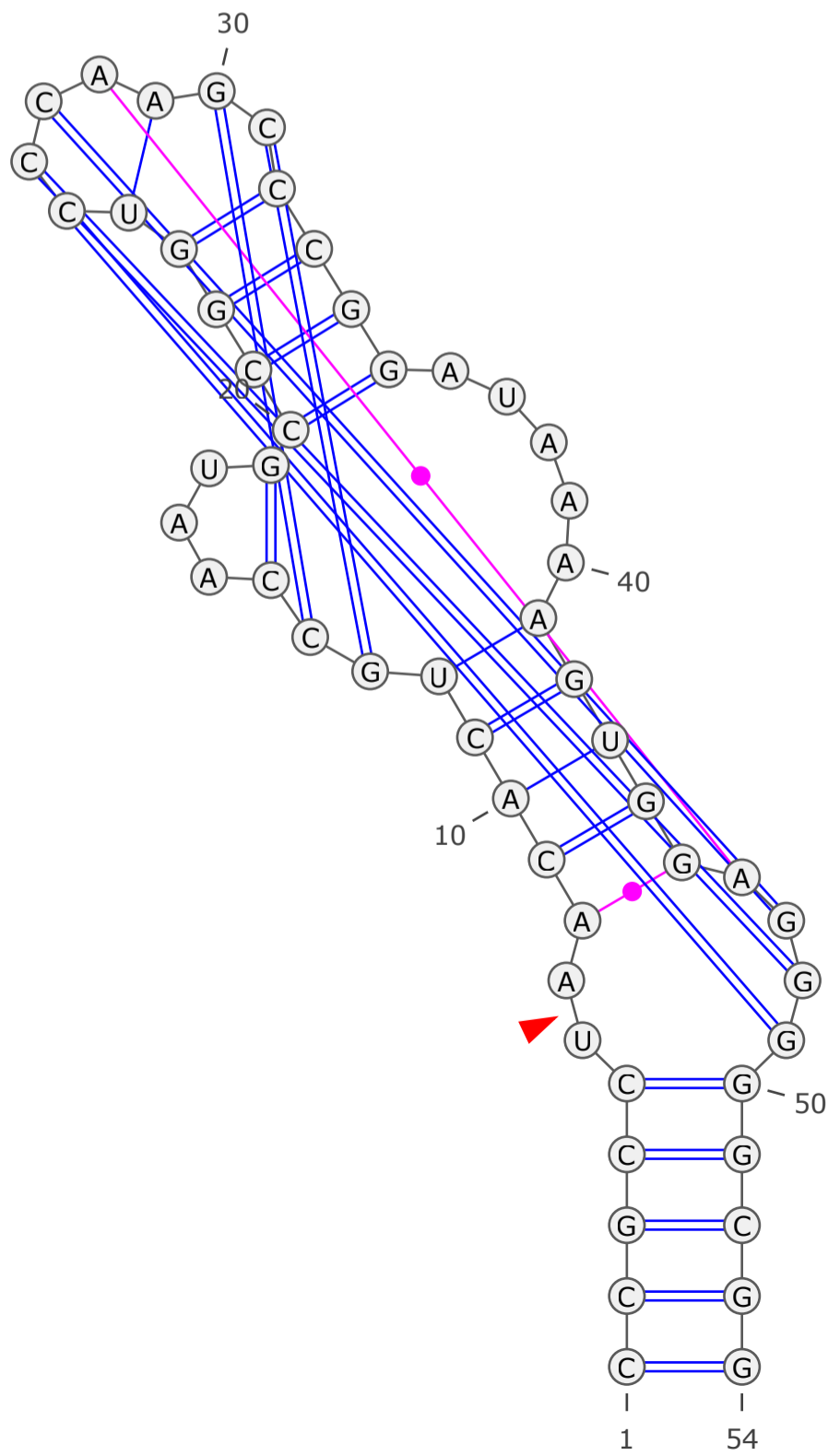

Supplement: qzae043_Supplementary_Data [file qzae043_supplementary_data.zip › Figure_S9.pdf]

**A**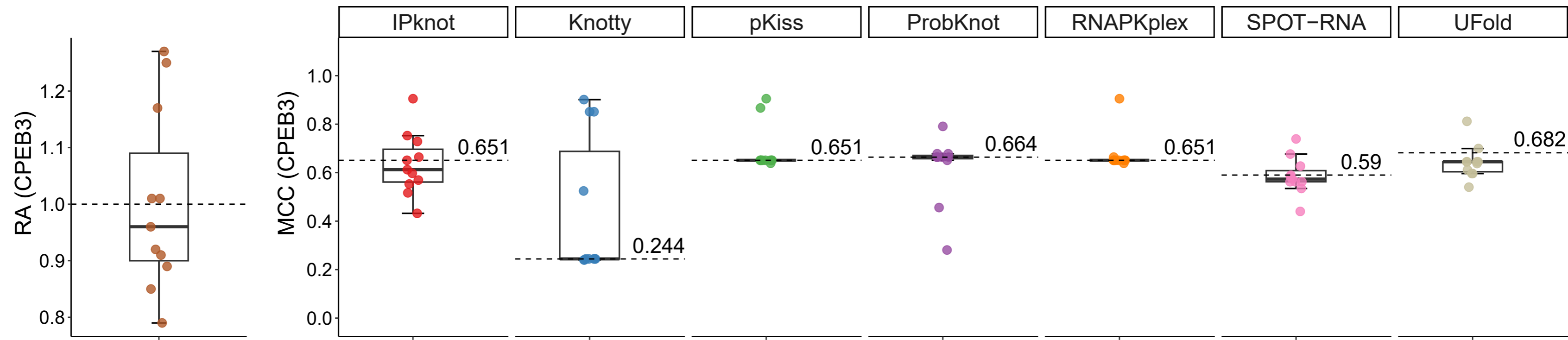**B**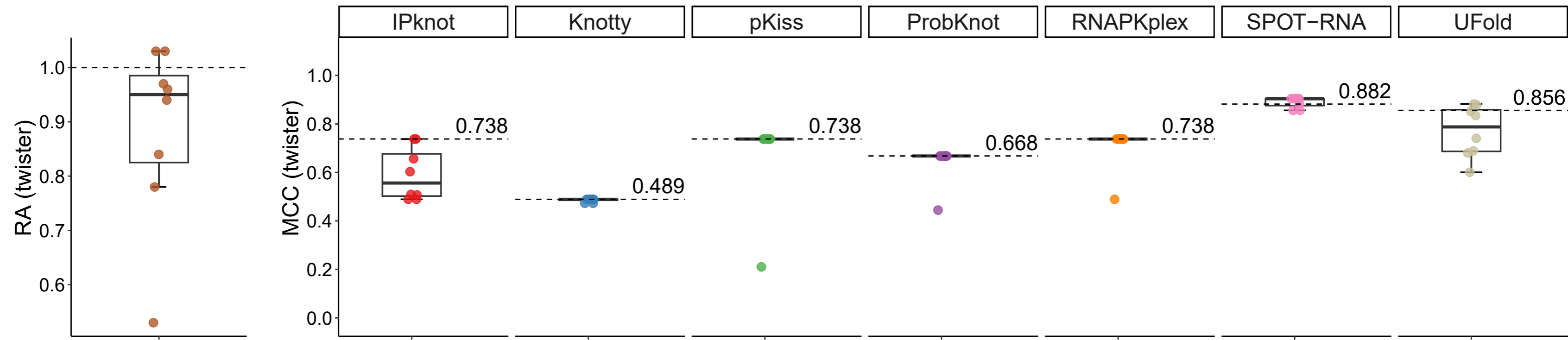

Supplement: qzae043_Supplementary_Data [file qzae043_supplementary_data.zip › Figure_S10.pdf]

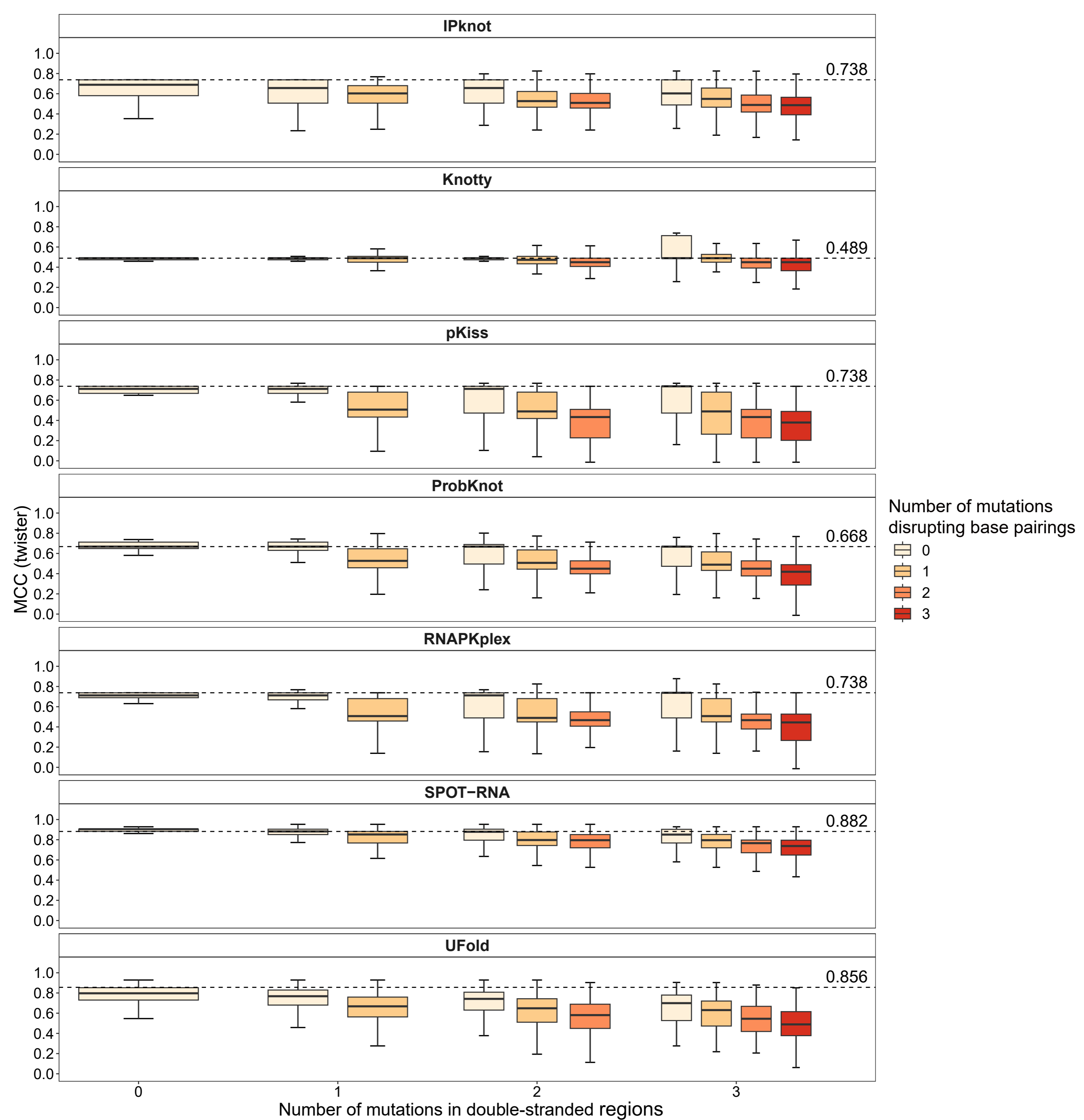

Supplement: qzae043_Supplementary_Data [file qzae043_supplementary_data.zip › Figure_S11.pdf]

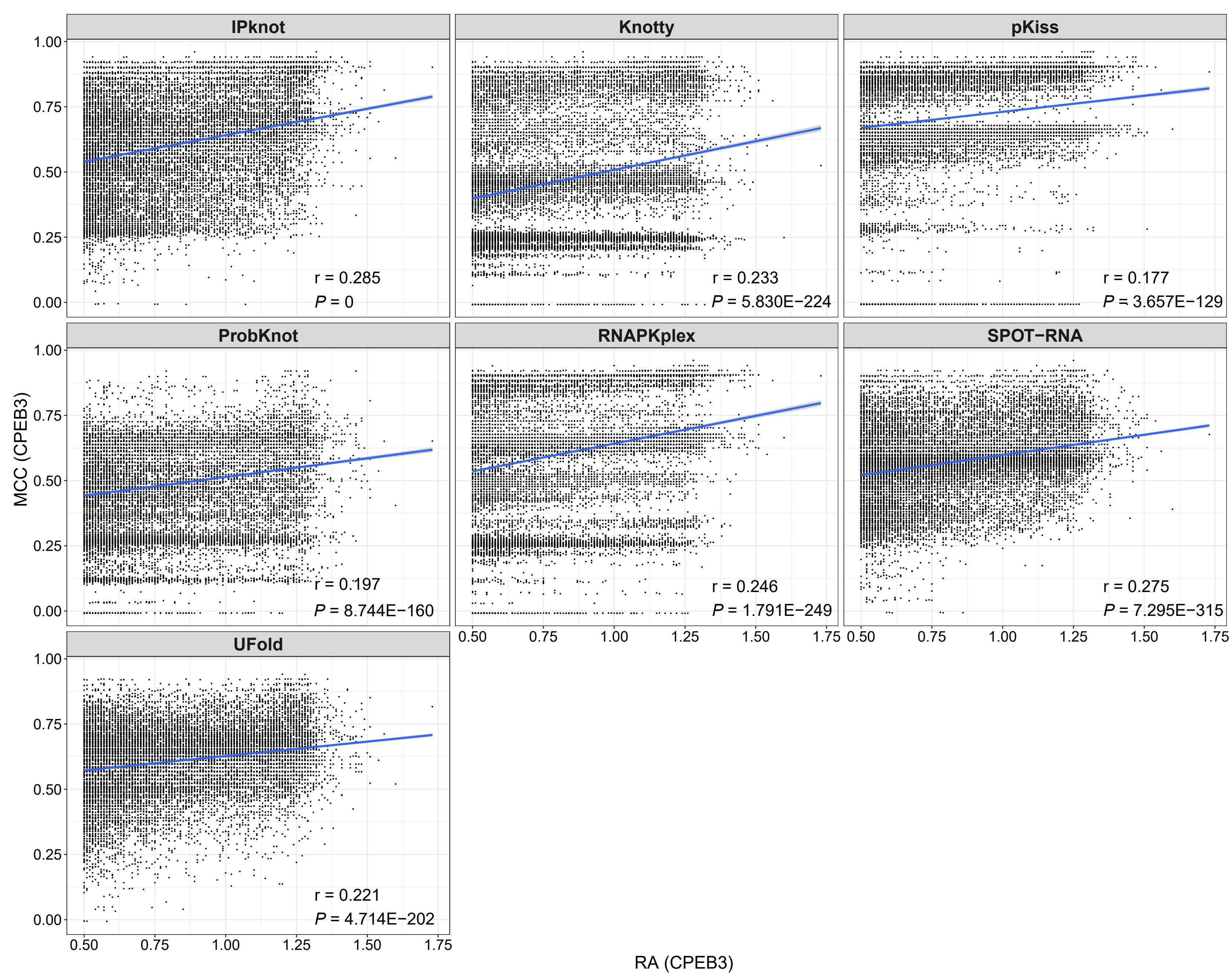

Supplement: qzae043_Supplementary_Data [file qzae043_supplementary_data.zip › Figure_S12.pdf]

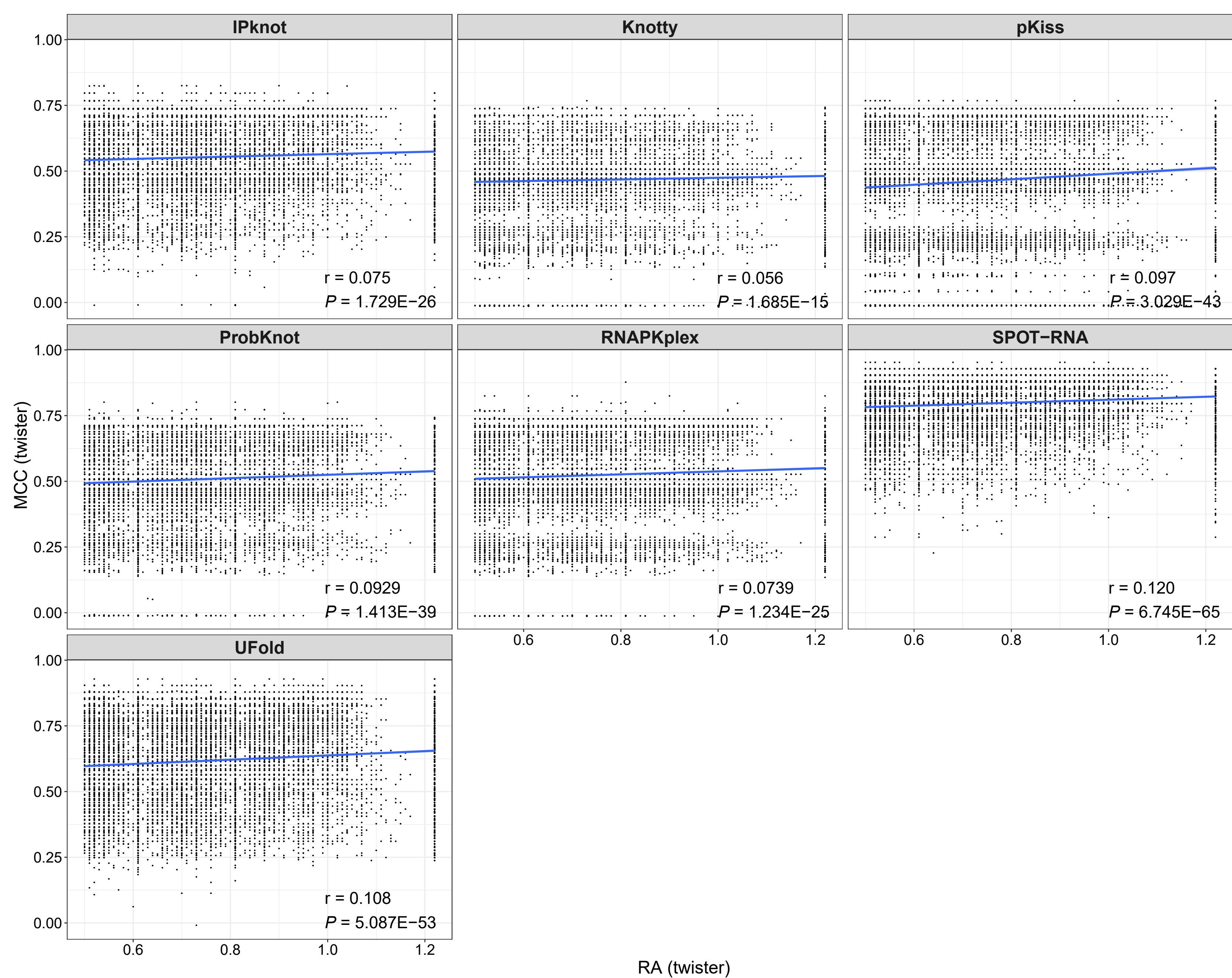

Supplement: qzae043_Supplementary_Data [file qzae043_supplementary_data.zip › Figure_S13.pdf]

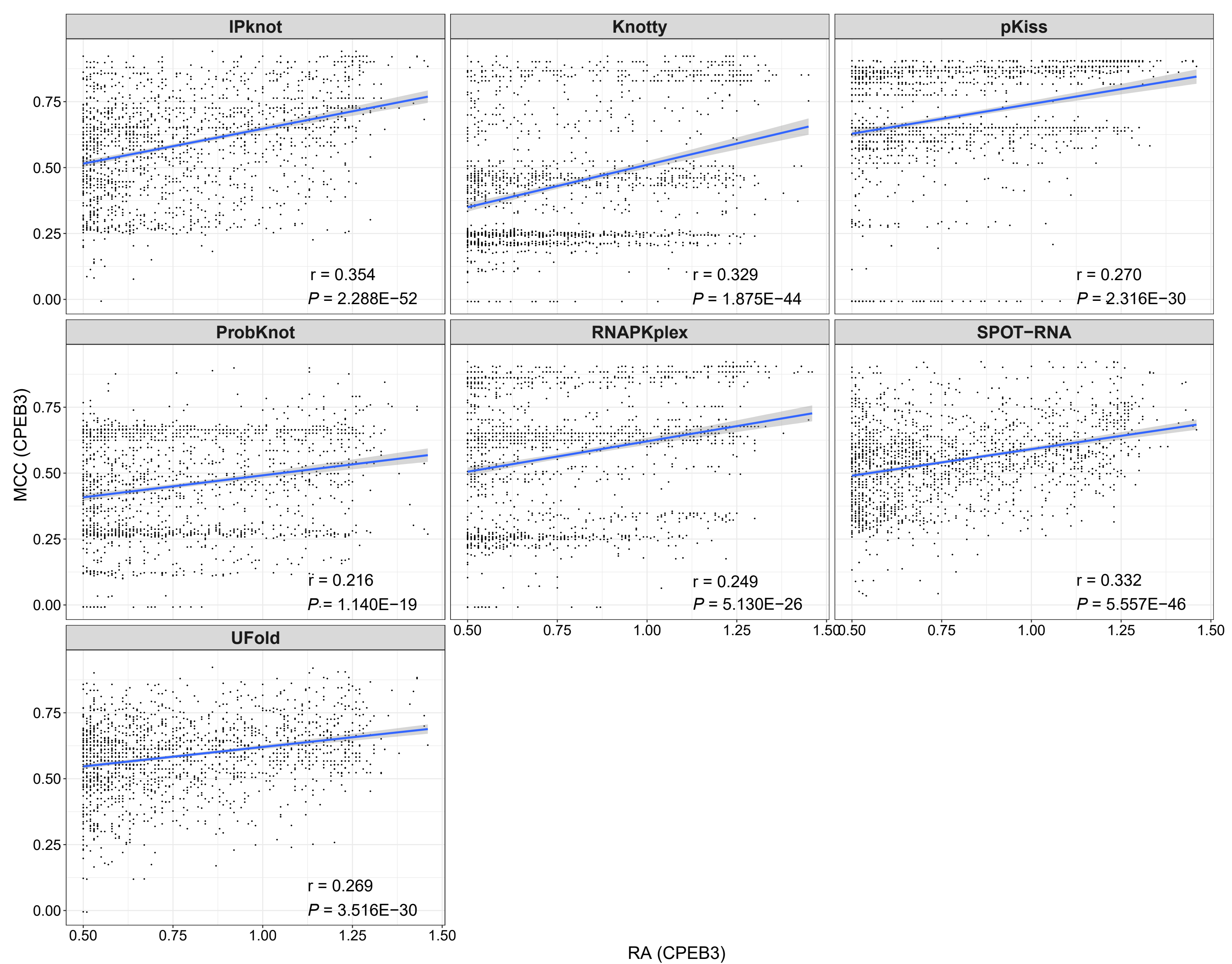

Supplement: qzae043_Supplementary_Data [file qzae043_supplementary_data.zip › Figure_S14.pdf]

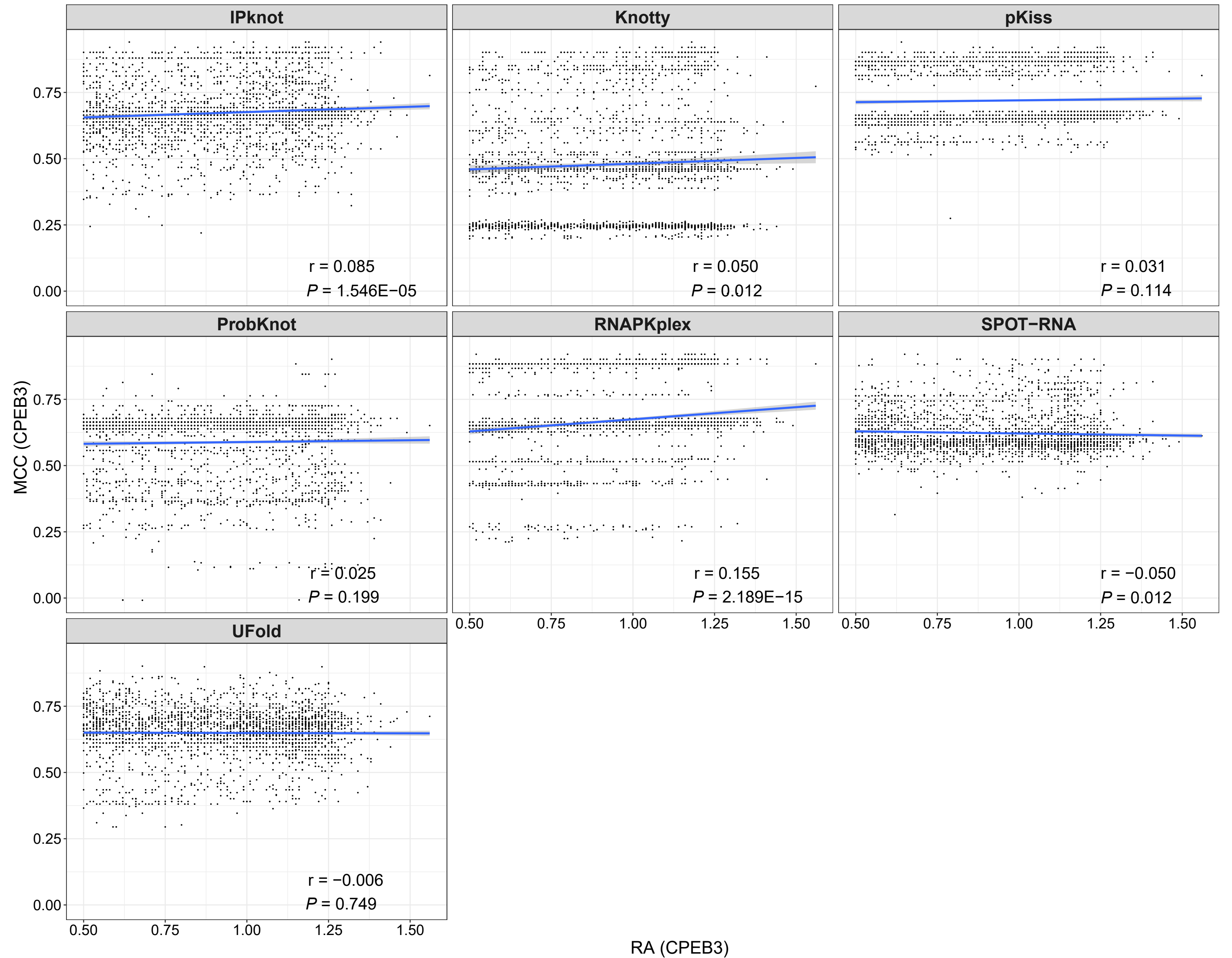

Supplement: qzae043_Supplementary_Data [file qzae043_supplementary_data.zip › Figure_S15.pdf]

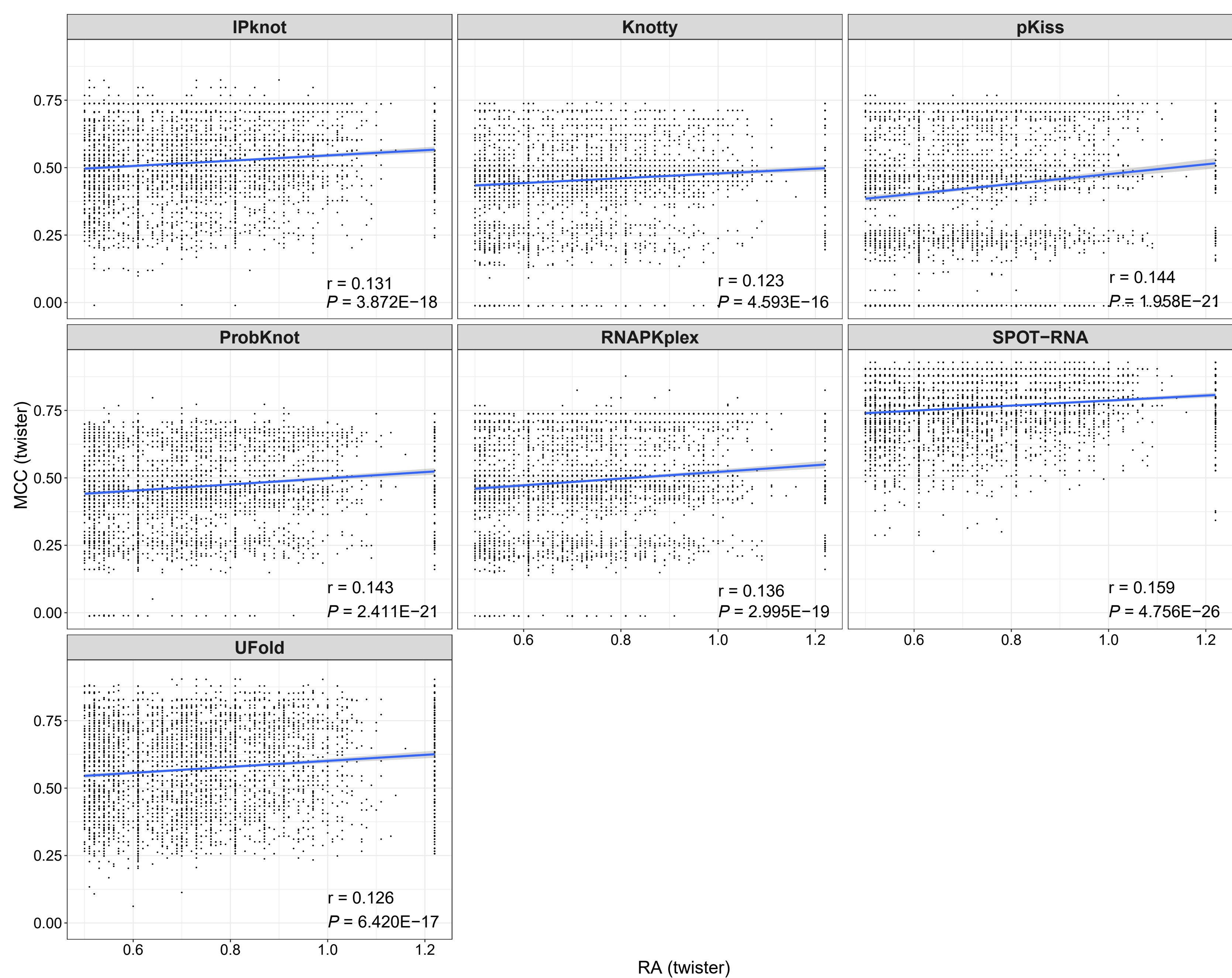

Supplement: qzae043_Supplementary_Data [file qzae043_supplementary_data.zip › Figure_S16.pdf]

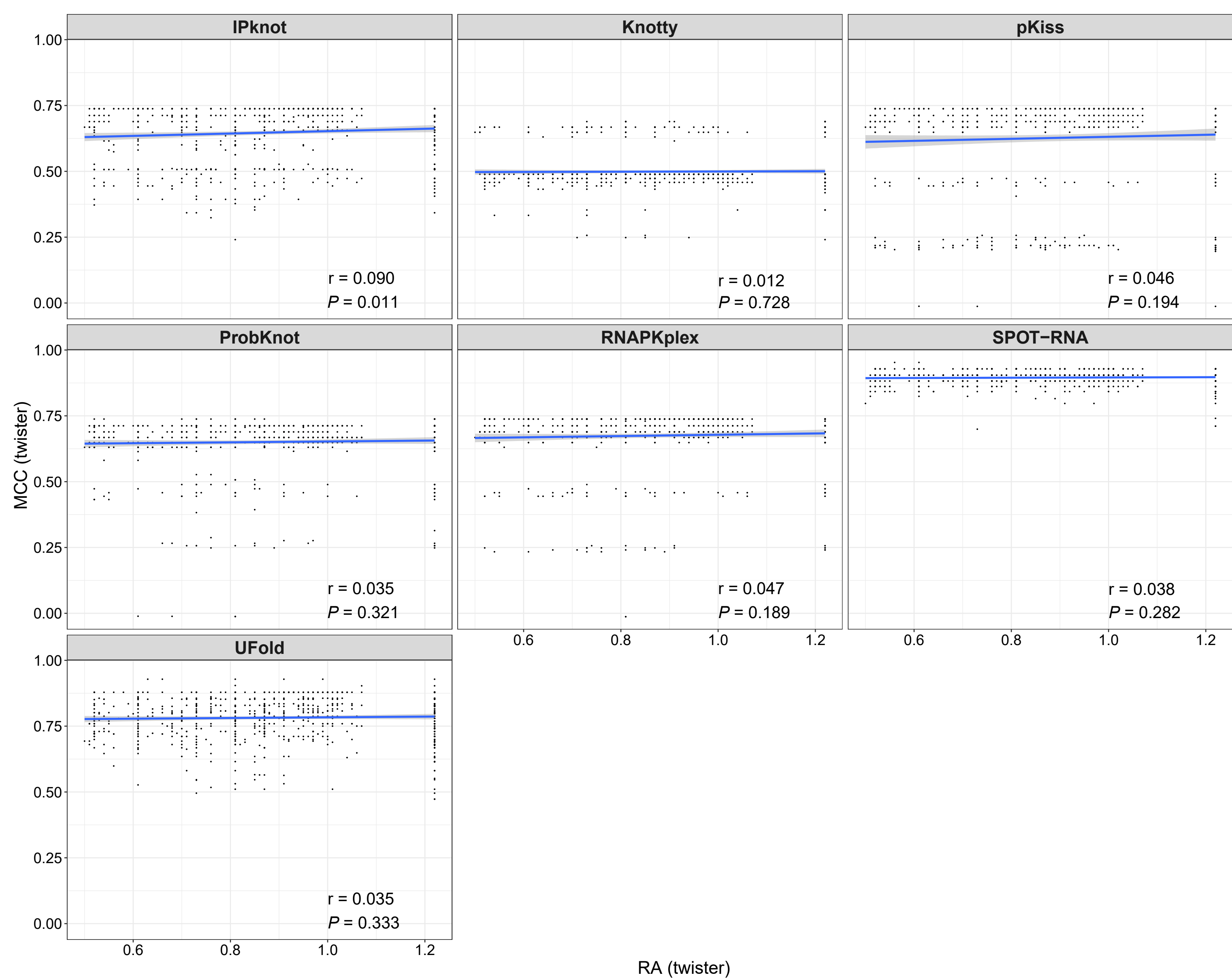

Supplement: qzae043_Supplementary_Data [file qzae043_supplementary_data.zip › Figure_S17.pdf]

**A**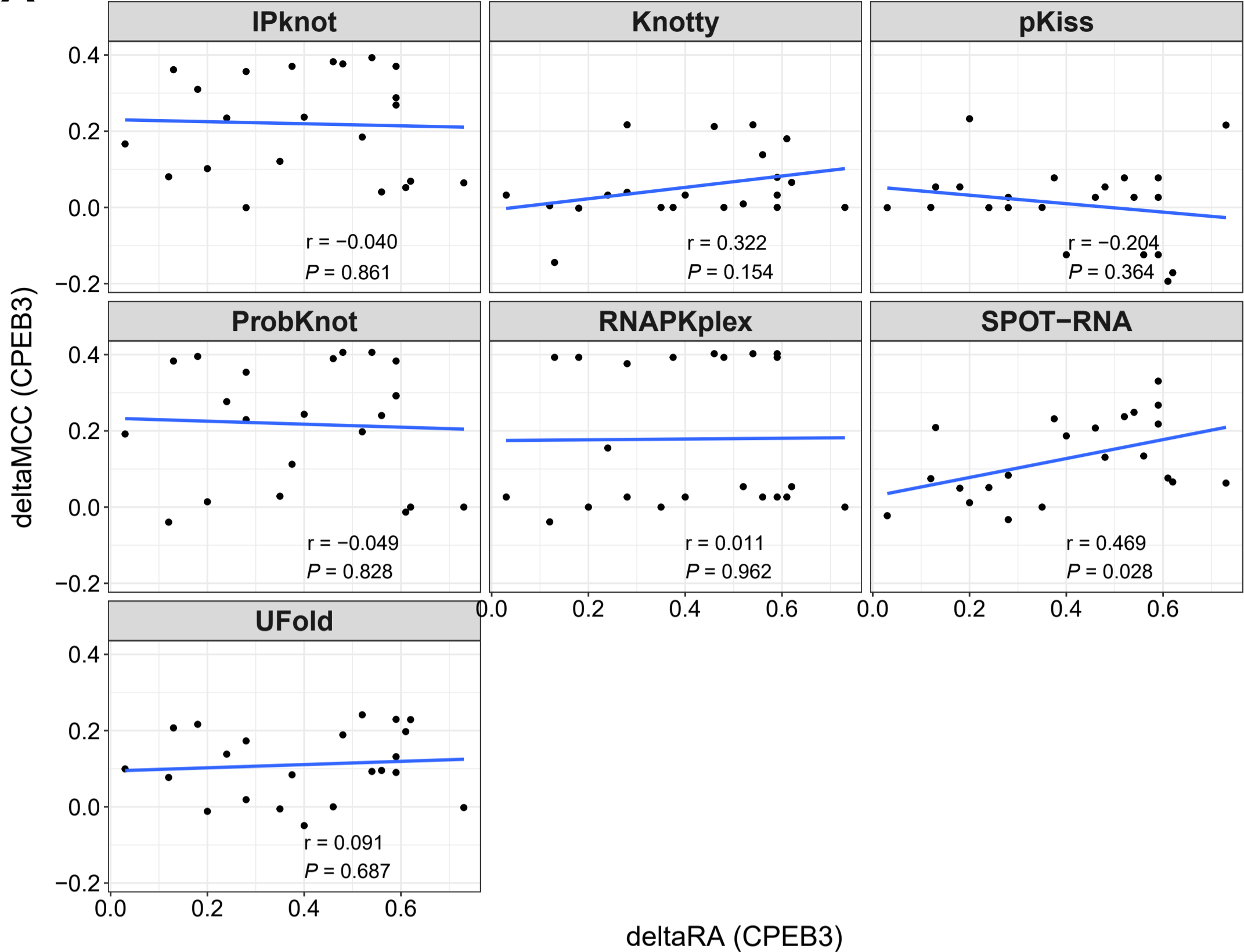**B**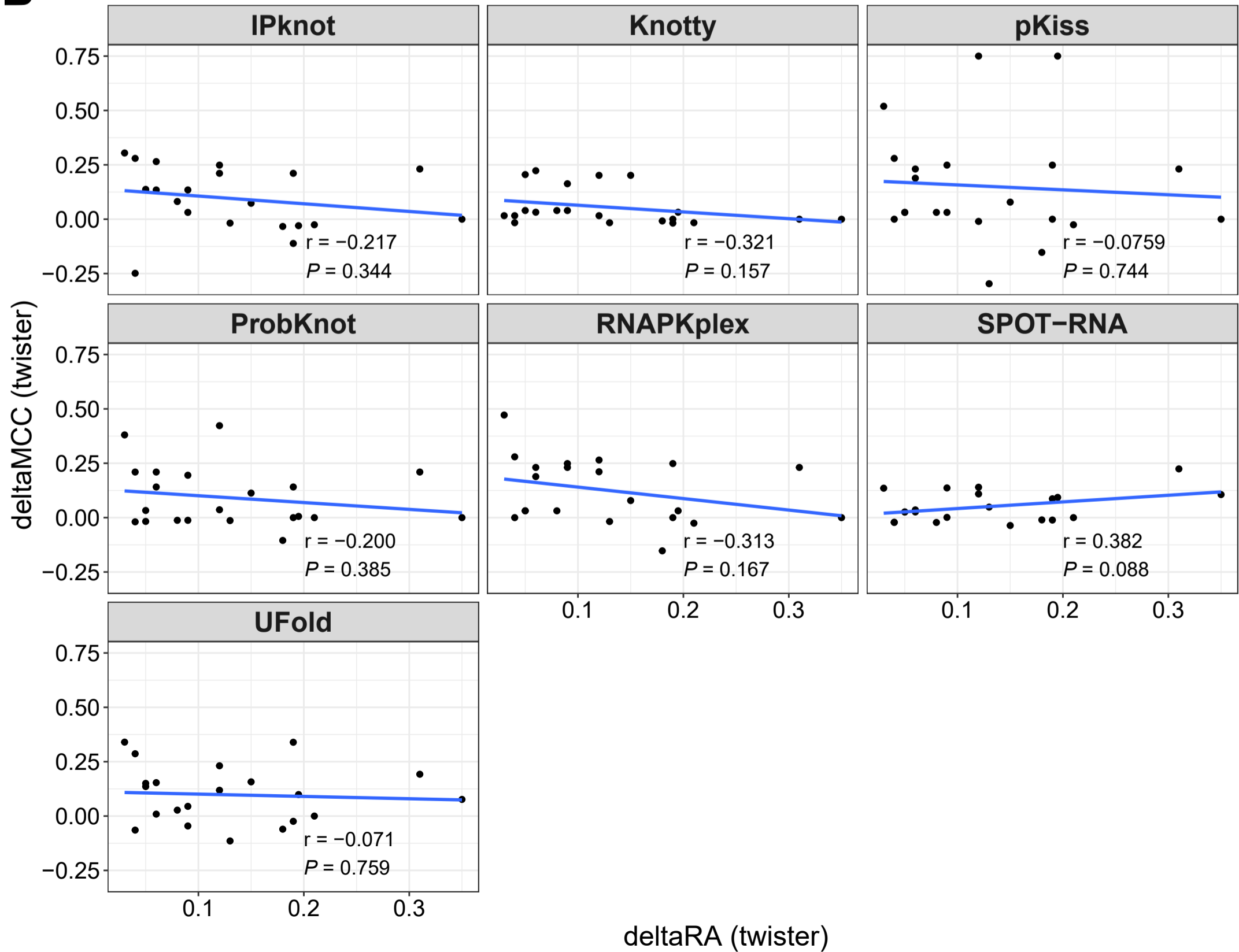

Supplement: qzae043_Supplementary_Data [file qzae043_supplementary_data.zip › Figure_S18.pdf]

**A**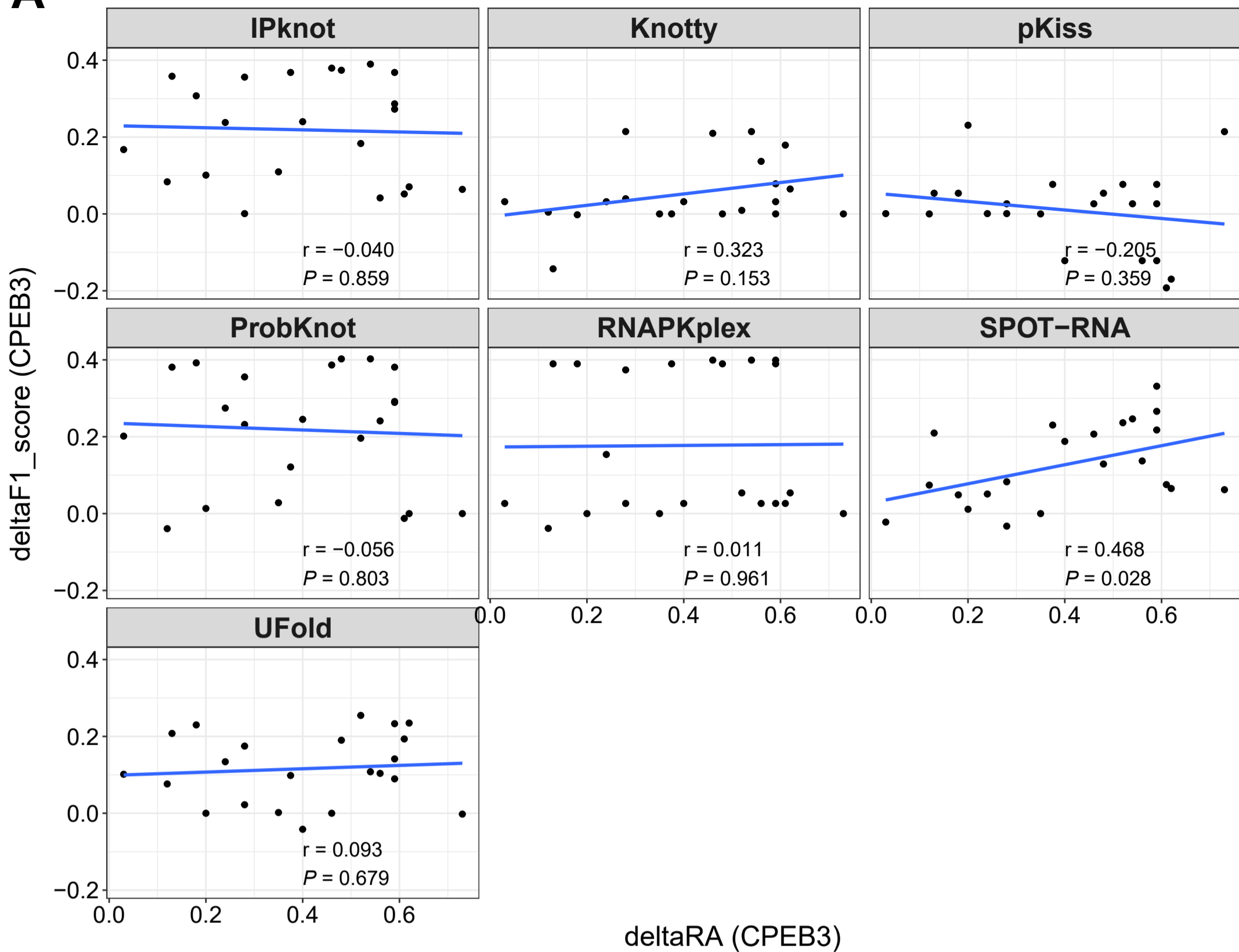**B**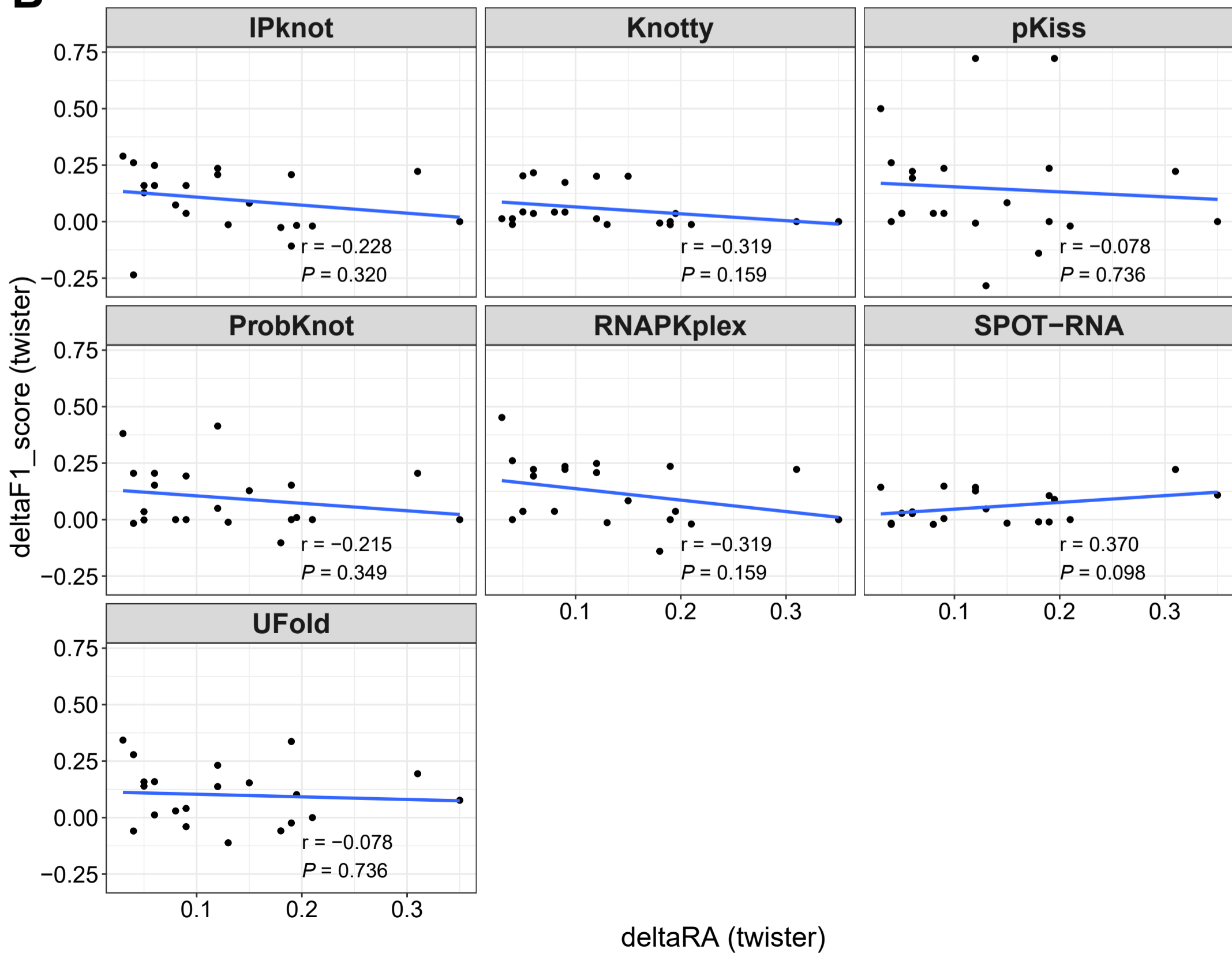

Supplement: qzae043_Supplementary_Data [file qzae043_supplementary_data.zip › Figure_S19.pdf]

**A**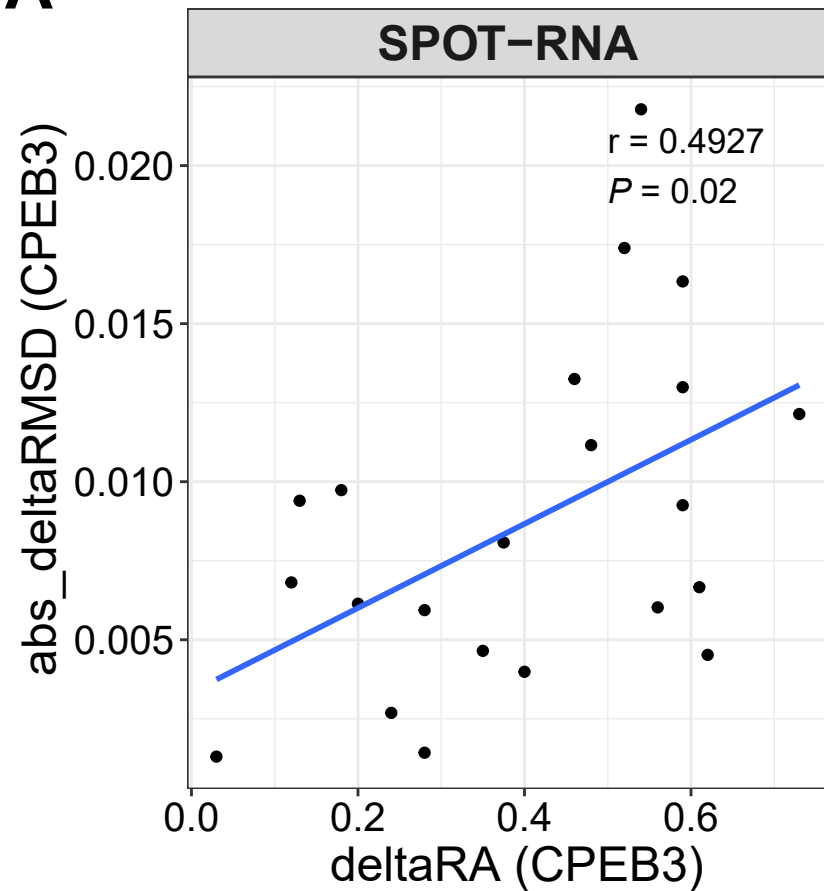**B**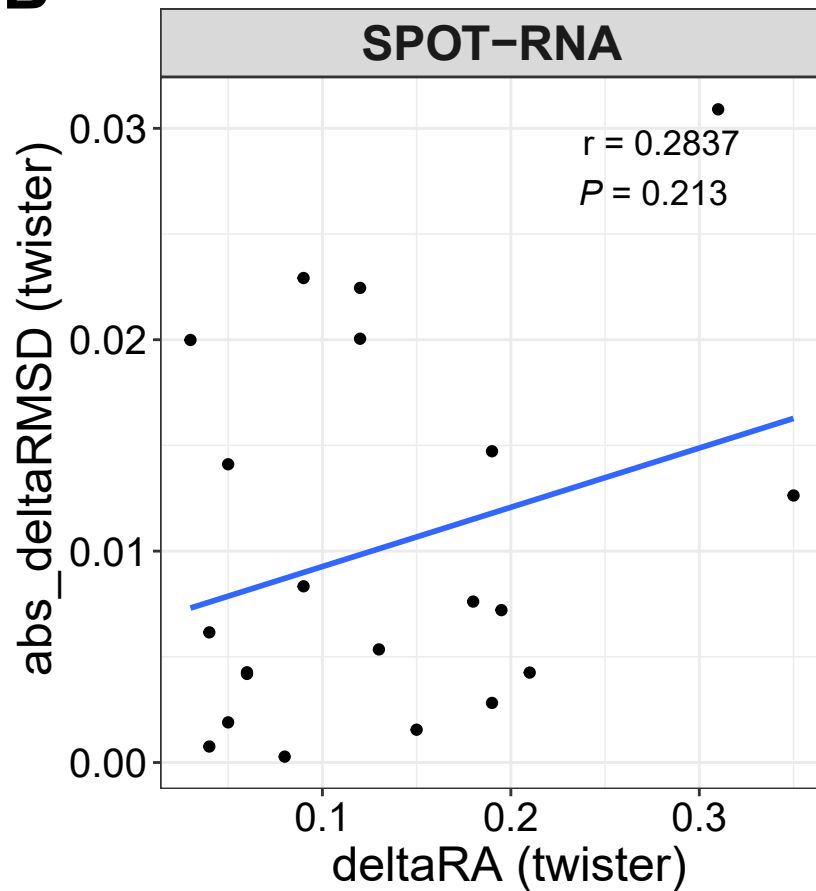

Supplement: qzae043_Supplementary_Data [file qzae043_supplementary_data.zip › Figure_S20.pdf]

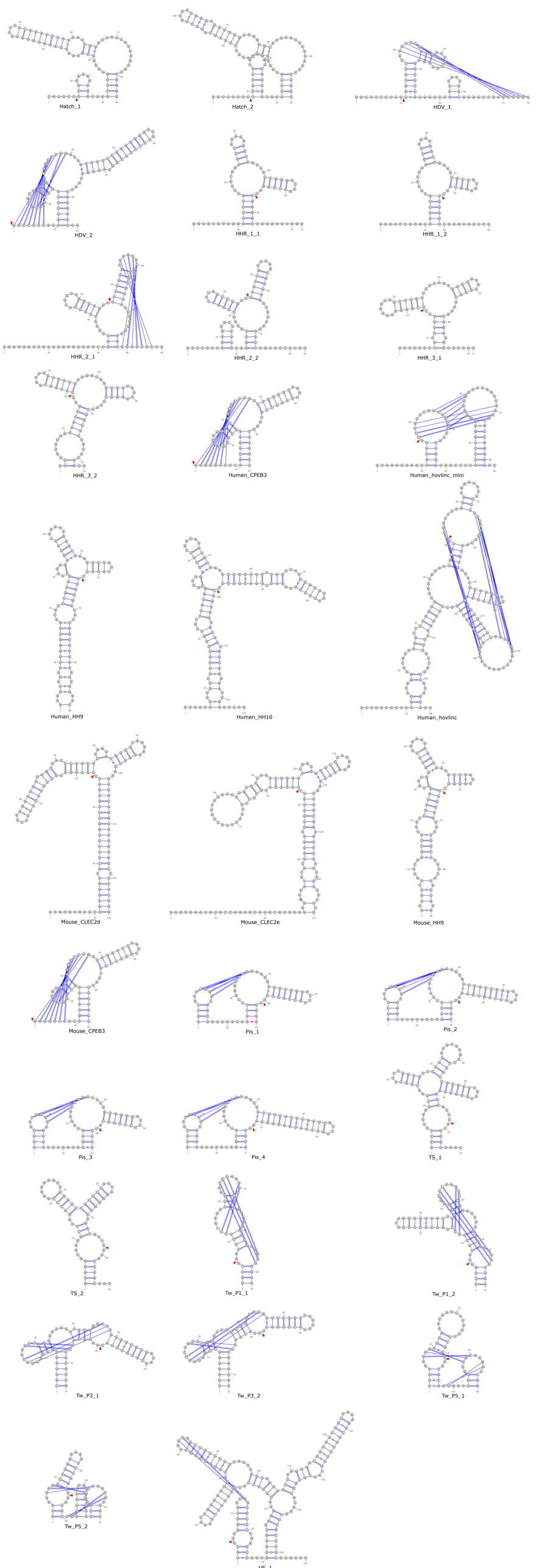

Supplement: qzae043_Supplementary_Data [file qzae043_supplementary_data.zip › Figure_S1.pdf]

MCC

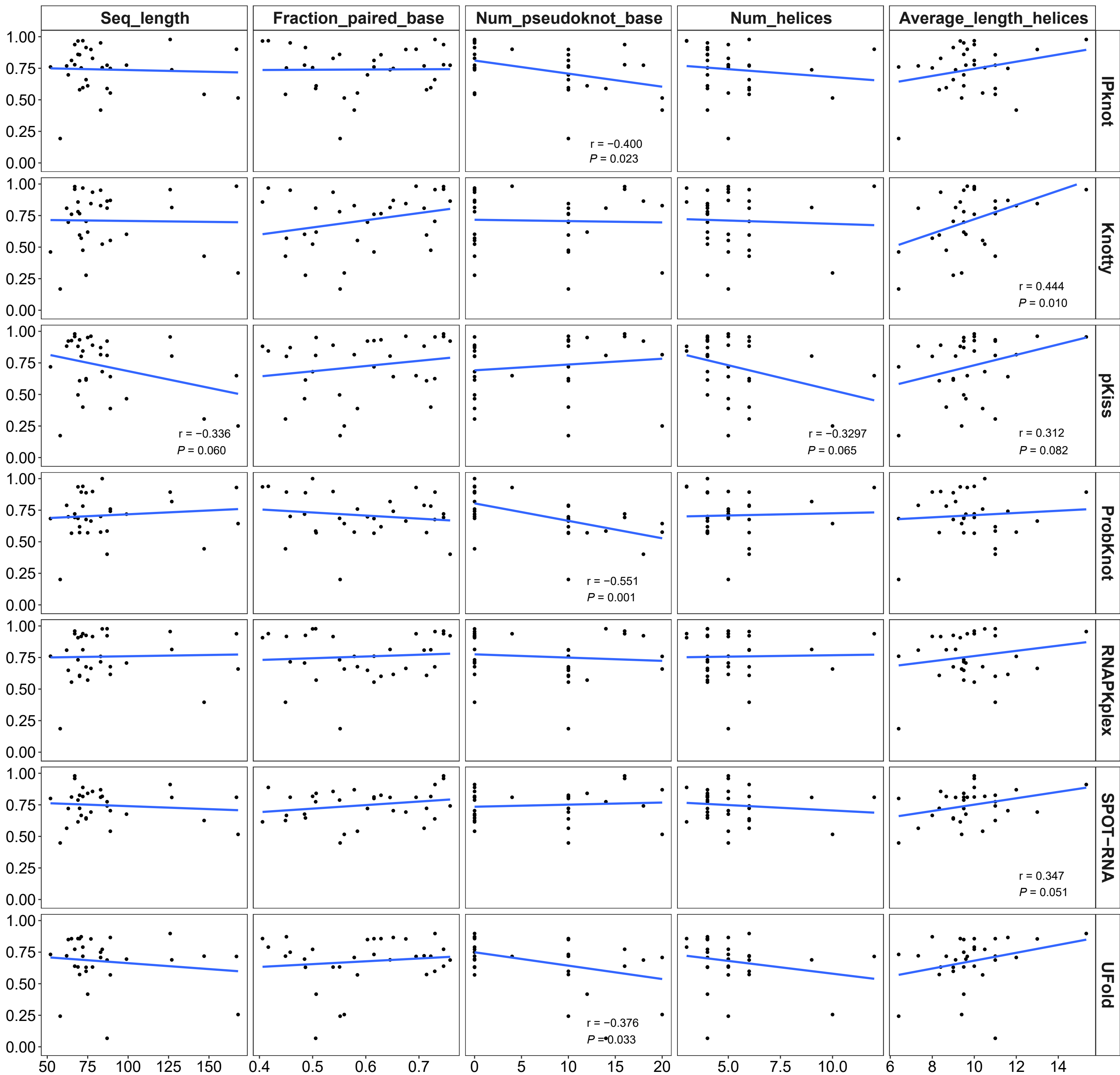

Supplement: qzae043_Supplementary_Data [file qzae043_supplementary_data.zip › Figure_S3.pdf]

A

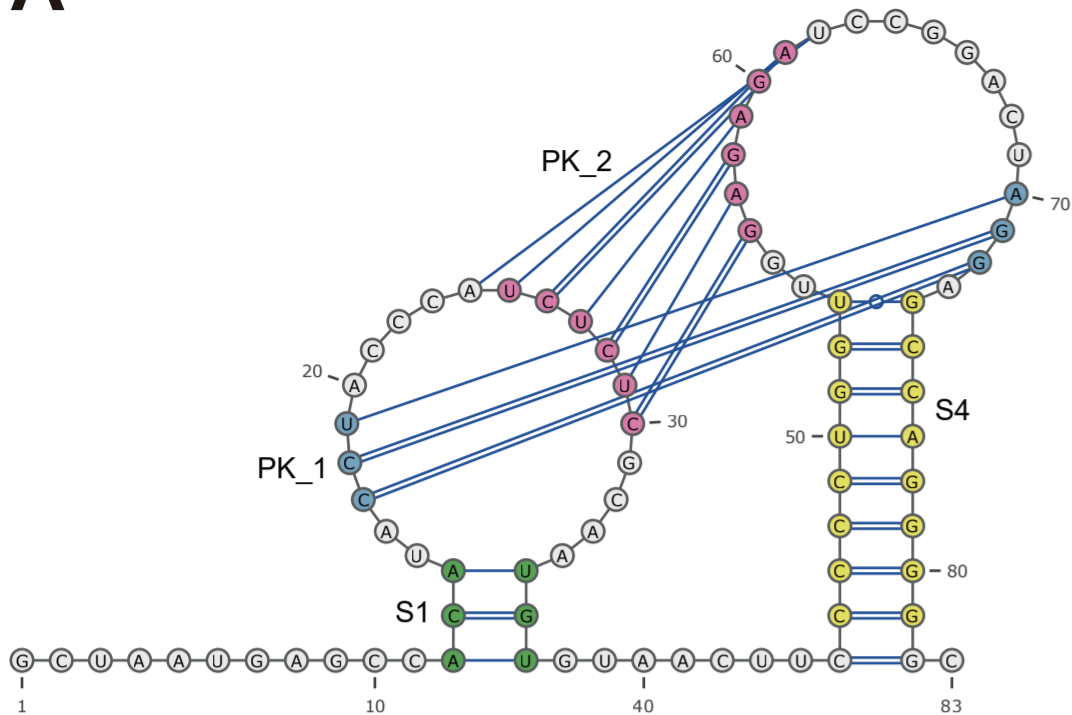

B

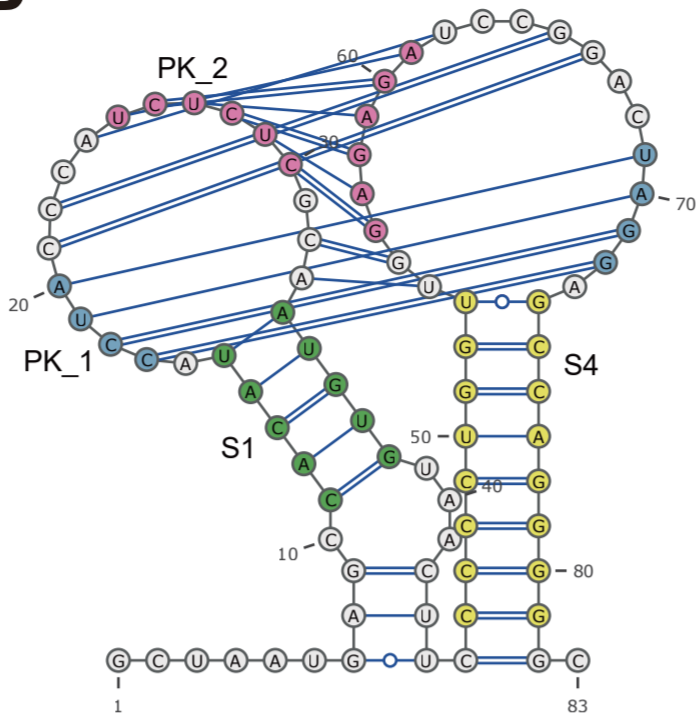

C

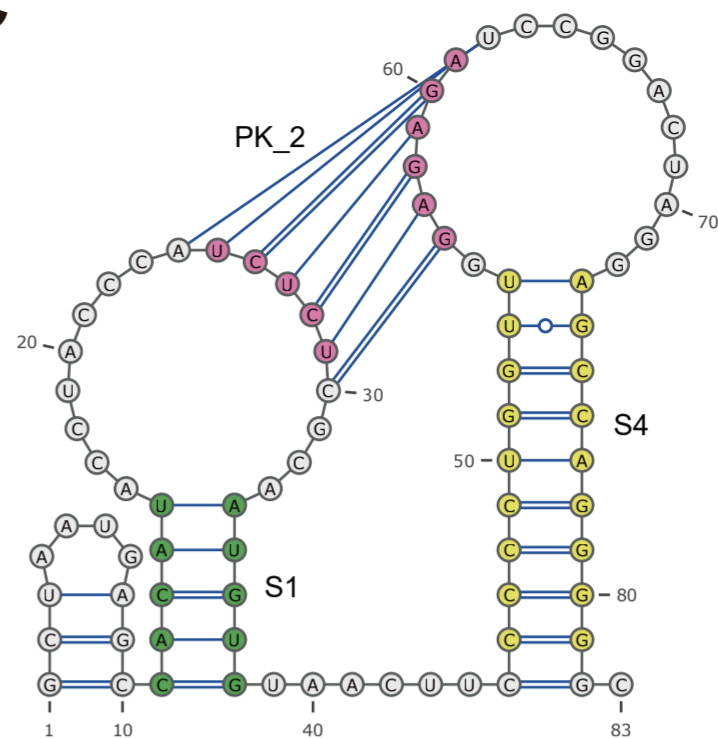

D

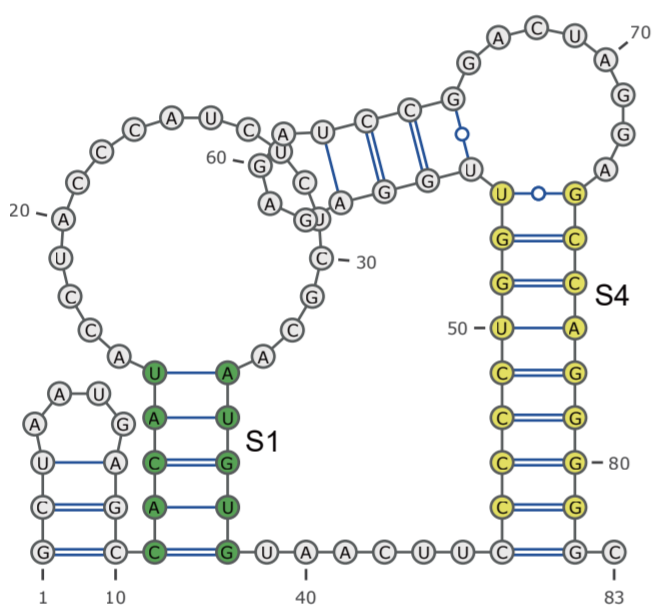

E

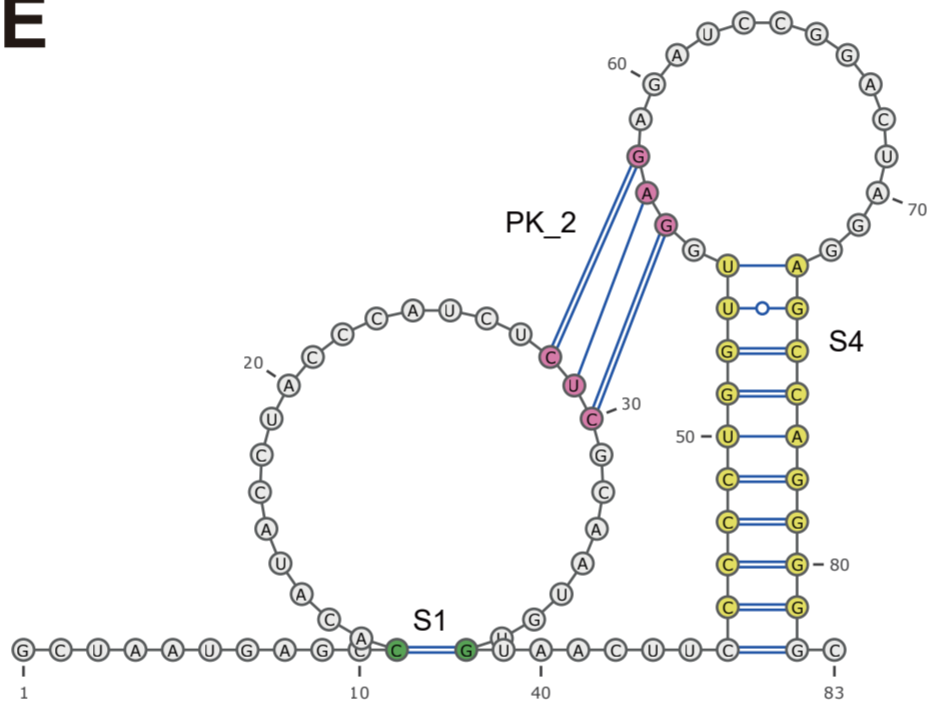

F

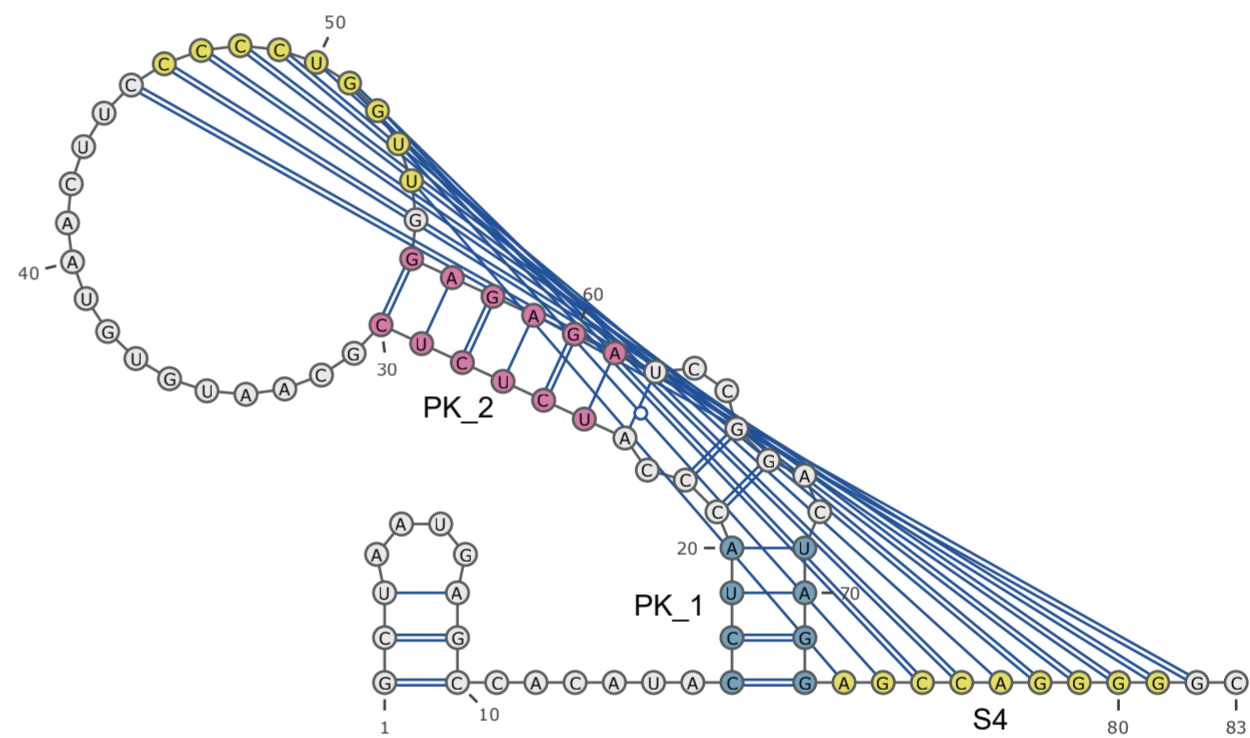

G

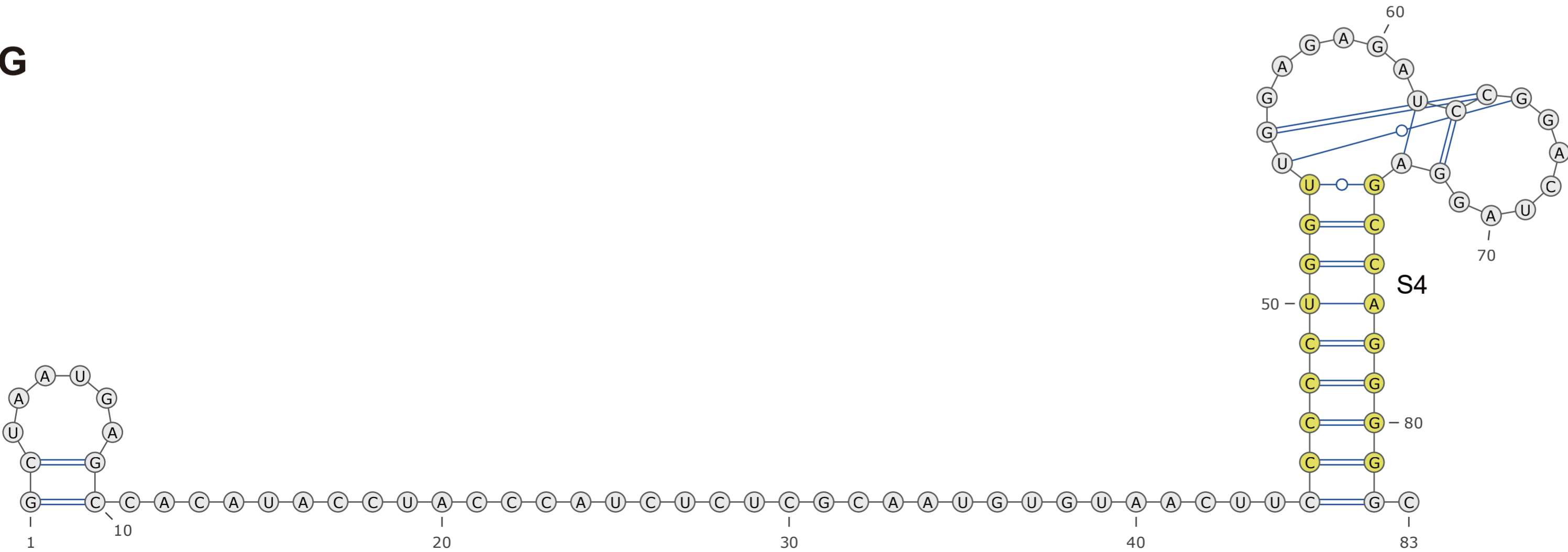

Supplement: qzae043_Supplementary_Data [file qzae043_supplementary_data.zip › Figure_S4.pdf]
